# Women's Health in Multiple Sclerosis: A Scoping Review

**DOI:** 10.3389/fneur.2021.812147

**Published:** 2022-01-31

**Authors:** Lindsay Ross, Huah Shin Ng, Julia O'Mahony, Maria Pia Amato, Jeffrey A. Cohen, Mary Pat Harnegie, Kerstin Hellwig, Mar Tintore, Sandra Vukusic, Ruth Ann Marrie

**Affiliations:** ^1^Department of Neurology, Mellen Center for Multiple Sclerosis Treatment and Research, Neurological Institute, Cleveland Clinic, Cleveland, OH, United States; ^2^Division of Neurology and the Djavad Mowafaghian Centre for Brain Health, Department of Medicine, University of British Columbia, Vancouver, BC, Canada; ^3^Department of Internal Medicine, Max Rady College of Medicine, Rady Faculty of Health Sciences, University of Manitoba, Winnipeg, MB, Canada; ^4^Department Neurofarba, Section of Neurosciences, University of Florence, Florence, Italy; ^5^Istituto di Ricovero e Cura a Carattere Scientifico (IRCCS), Fondazione Don Carlo Gnocchi, Florence, Italy; ^6^Cleveland Clinic, Cleveland, OH, United States; ^7^Department of Neurology, Ruhr University, Bochum, Germany; ^8^Multiple Sclerosis Centre of Catalonia, Department of Neurology-Neuroimmunology, Hospital Universitari Vall d'Hebron, Universitat Autonoma de Barcelona, Universitat de Vic - Universitat Central de Catalunya, Barcelona, Spain; ^9^Service de Neurologie A et Fondation Eugène Devic EDMUS pour la Sclérose en Plaques, Hôpital Neurologique Pierre Wertheimer, Hospices Civils de Lyon, Lyon, France; ^10^Centre des Neurosciences de Lyon, INSERM 1028 et CNRS UMR5292, Observatoire Français de la Sclérose en Plaques, Lyon, France; ^11^Université Claude Bernard Lyon 1, Villeurbanne, France; ^12^Department of Community Health Sciences, Max Rady College of Medicine, Rady Faculty of Health Sciences, University of Manitoba, Winnipeg, MB, Canada

**Keywords:** multiple sclerosis, scoping review, pregnancy, menopause, women's health

## Abstract

**Background:**

Women with multiple sclerosis (MS) may face challenges related to managing reproduction, pregnancy, and menopause while simultaneously managing their disease. The purpose of this scoping review was to map the literature broadly related to topics relevant to women's health in MS to inform the clinical and research communities about the existing types and sources of evidence and knowledge gaps. Apart from coverage of topics within the field of women's health, we were interested in potential gaps related to geographic and racial and ethnic diversity. We also aimed to understand the degree of inclusion of women with progressive MS in this research.

**Methods:**

We searched the EMBASE and Ovid Medline databases from 1980 until November 23, 2020. We included case-control and cohort studies, clinical trials and case series published in any language, conducted in women with MS, clinically isolated syndrome, or radiologically isolated syndrome, that addressed women's health. Two reviewers independently screened abstracts and full-text reports for study inclusion, and completed data extraction.

**Results:**

Of 112,106 citations screened, 1,041 underwent full-text review and 353 met the inclusion criteria. The number of studies regarding women's health has increased exponentially over time. Almost half of the studies were conducted (at least in part) in Europe, while 21.7% were conducted in North America; only one study was conducted in Africa. Most studies did not report the race or ethnicity of their participants (*n* = 308, 87.2%). Among the 353 studies, 509 topics were reported as some studies addressed more than one topic. Over one-third of these focused on pregnancy (*n* = 201, 37.2%), followed by fetal/neonatal outcomes (14.4%) and sexual dysfunction (10%). Among the 201 studies that focused on pregnancy, only 51 (25.4%) included participants with progressive MS.

**Conclusions:**

This review identifies important knowledge gaps related to women's health in MS and particularly the need for future studies to include participants with a broader range of races and ethnicities, with progressive MS, and living in Asia-Pacific and African regions.

## Introduction

Historically, there was a paucity of research on women's health concerns, severely limiting the information available to women and their healthcare providers (1). More recently, there has been a substantial shift, with growing support from governmental and non-governmental institutions for women's health research accompanied by organization structural changes and policy revisions. The result has been an influx of women's health research, which is starting to fill the previous void (2). One area where women's health research is of particular importance is within the context of chronic disease management.

Multiple sclerosis (MS) is a chronic disease of the central nervous system affecting nearly three million people worldwide (3). Two to three times as many women as men are affected by MS (4, 5). Women with MS may face challenges related to managing reproduction, pregnancy, and menopause while simultaneously managing their disease. For example, multiple physiologic changes occur during pregnancy, including hypercoagulability, insulin resistance, immunotolerance to the fetoplacental unit and increased plasma volume. Adaptation to these changes may be harder in the context of chronic disease (6). Also, numerous disease-modifying therapies have emerged for the management of MS, but most are not recommended for use during pregnancy. Thus, women with MS must balance management of their disease with the health of the fetus. While research surrounding the experience and management of women's health issues in MS has increased over time, women living with MS, clinicians, and researchers recognize that knowledge gaps remain.

We aimed to conduct a scoping review. Scoping reviews constitute a newer form of evidence synthesis which provides an overview of available research evidence, in contrast to systematic reviews which summarize evidence related to a focused question (7). The purpose of this scoping review was to map the literature broadly related to topics relevant to women's health in MS to inform the clinical and research communities about the existing types and sources of evidence and knowledge gaps. Apart from coverage of topics within the field of women's health, given the relative lack of diversity in clinical trials at large (8) as well within the MS field (9, 10), we were particularly interested in potential gaps related to geographic and racial and ethnic diversity. We also aimed to understand the degree of inclusion of women with progressive MS in this research (11). We expected that this effort would inform future research efforts and policy.

## Methods

We used the methods for scoping reviews delineated by Arksey and O'Malley, as updated by the Joanna Briggs Institute (7). We report this review according to the PRISMA Extension for Scoping Reviews (12).

### Identification of Relevant Literature

Lists of potential key words were developed by neurologists specializing in MS, with expertise in epidemiology, systematic reviews, and women's health in MS based on group discussion, and review of relevant systematic and scoping review search strategies in other chronic diseases. Subsequently, a medical librarian (MPH) with expertise in systematic reviews and scoping reviews developed the formal search strategy and searched the literature for records including the concepts of MS and women's health. The librarian created search strategies using a combination of keywords and controlled vocabulary in Ovid Medline and EMBASE for the period 1980 onward; 1980 was chosen to coincide with the 1983 Poser criteria for MS (13) and focus the review on contemporary research of women's health issues for persons with MS. The searches were completed on November 23, 2020 without language limits. Appendix I shows the full search strategies. We also manually searched the references lists of included articles. All database search results were imported into Endnote™ and de-duplicated before screening.

### Inclusion and Exclusion Criteria

We included studies that met the following criteria: (i) case-control, cohort, clinical trial, and case series (14); (ii) published in any language (studies that were not published in English were translated using Google Translate); (iii) conducted in women with MS, clinically isolated syndrome (CIS), or radiologically isolated syndrome (RIS); and (iv) addressed one of the following women's health topics of interest: birth control, pregnancy, menstruation, breastfeeding, fertility, assisted reproduction, fetal/neonatal outcomes, menopause, children's health/developmental milestones, gynecologic cancer or related screening activities/prevention, sexually transmitted infections, sexual dysfunction, gender identity or sexual orientation, and sex hormones. Studies that included MS and non-MS populations were included provided that they reported results separately in women with MS. Studies that included men and women with MS were included provided that they reported results separately in women. We excluded studies: (i) in animals or conducted *in-vitro*; (ii) study protocols, opinion documents, editorials, or commentaries; (iii) case reports; (iv) systematic reviews or meta-analyses; (v) limited to populations with neuromyelitis optica spectrum disorders (NMOSD) or myelin oligodendrocyte glycoprotein antibody disease (MOGAD); and (vi) limited to men with MS.

### Study Selection

We used a multistep process to select the studies and conduct the data extraction. Titles and abstracts were screened independently for inclusion by pairs of team members for relevance using EPPI-Reviewer software (15). Disagreements were resolved by consensus between the two members of the pair; if consensus could not be reached a third member was engaged. Subsequently, the selected articles underwent full-text review for relevance by two team members using a similar process. At each step in the process the reviewers used a standardized form in EPPI-Reviewer that delineated the *a priori* inclusion and exclusion criteria.

### Data Extraction

One reviewer extracted data from the selected articles using a data collection tool implemented in Eppi-Reviewer software, and the extraction was verified by a second reviewer. The data elements extracted included title, author (year), language, sample size, region where the study was performed, data source, study design, characteristics of the study population, diagnostic criteria used, and women's health topic (see Appendix II for data extraction tool). Three reviewers tested the data extraction tool on the first 30 articles, following which adjustments were made to clarify data element descriptors or add additional options (e.g., not reported) were added for specific data elements.

The focus of this scoping review was on what topics had been studied and who participated in those studies. Therefore, we did not extract information about study results or assess the quality of the included studies.

### Synthesis and Presentation of Results

We summarized the literature according to the data elements extracted using descriptive statistics.

## Results

### Results of Search

**Figure 1:**
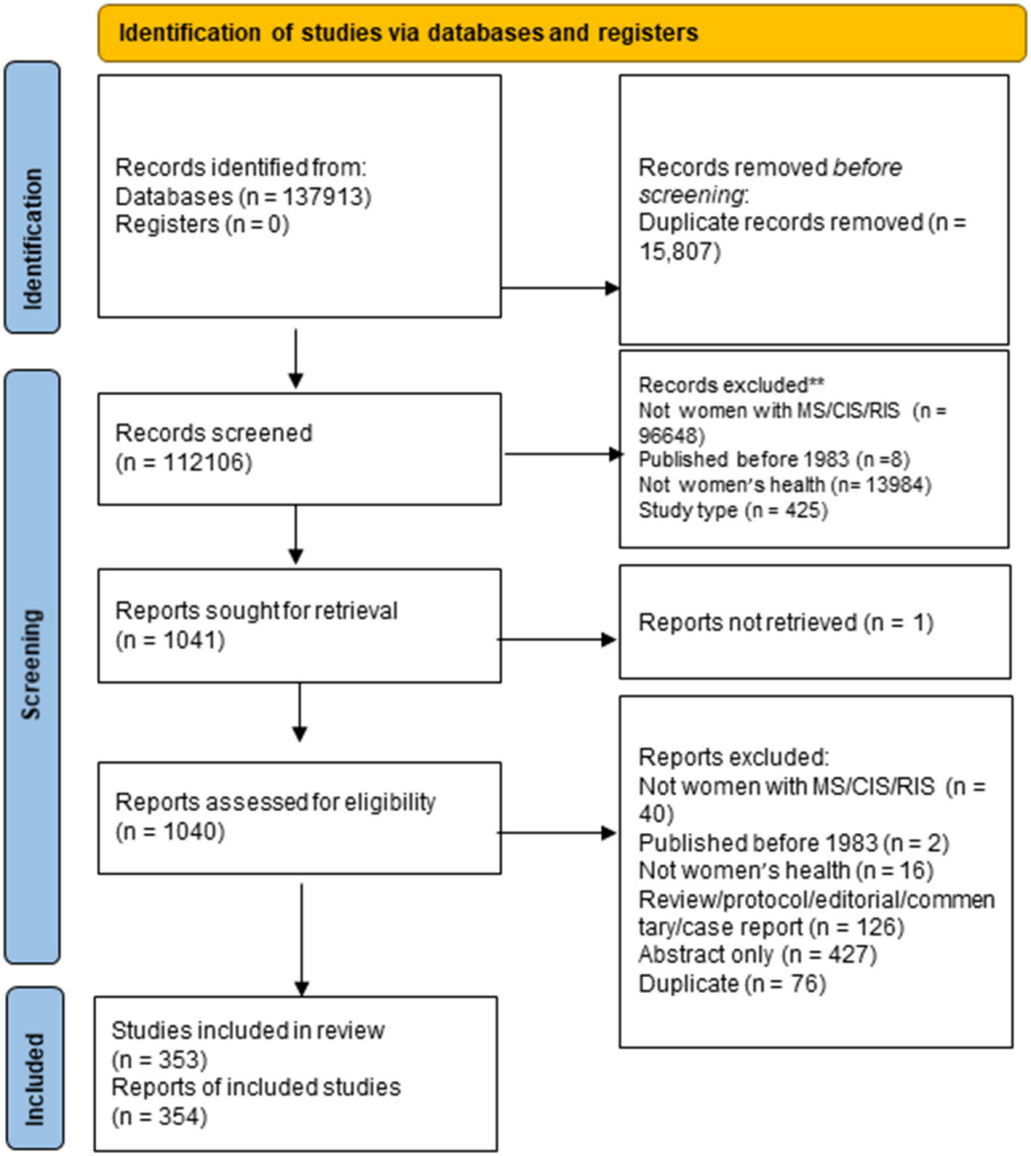
PRISMA flow diagram.

The searches identified a total of 137,913 citations, of which 15,807 were duplicates, resulting in 112,106 citations for review. Of these, 111,065 were excluded at the title/abstract review stage (Figure 1). Of 1,041 articles selected for full-text review, we excluded 685. We could not locate full-text articles for one. After full-text review, we retained 353 articles for data extraction.

### Study Characteristics

**Figure 2:**
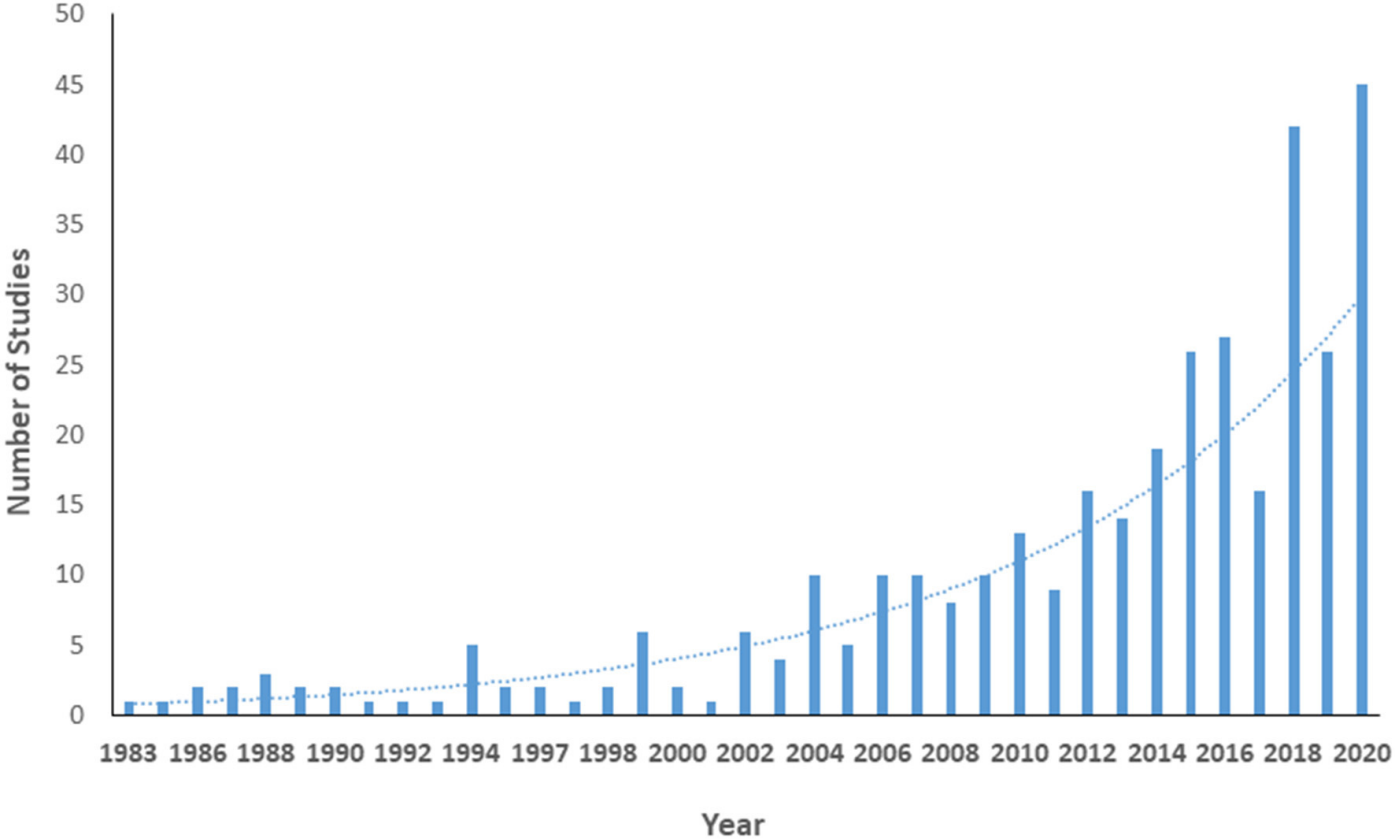
Number of publications regarding women's health in multiple sclerosis by year.

**Figure 3:**
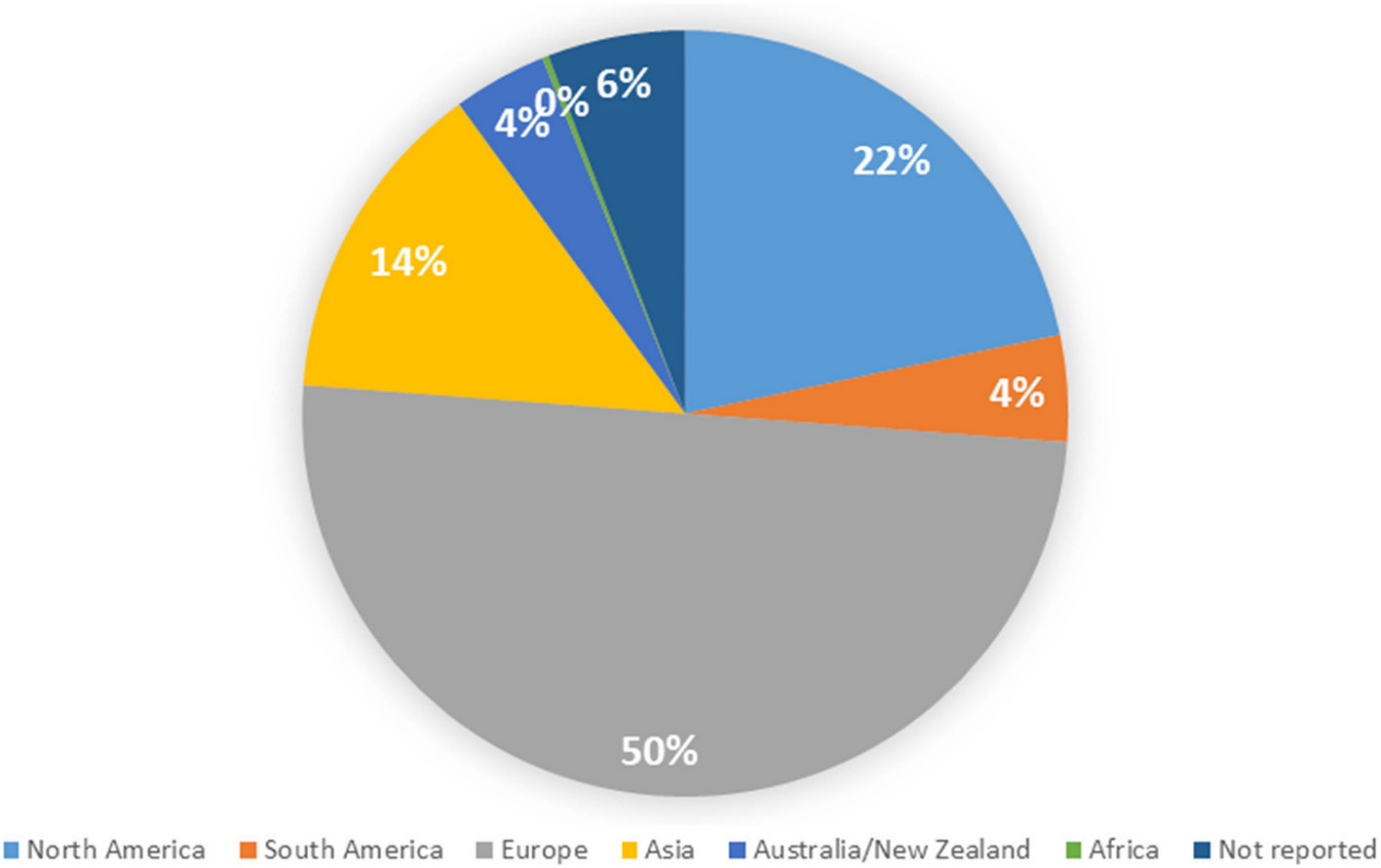
Distribution of included studies by geographic region.

Appendix III shows details of the included studies. Consistent with our search parameters, study publication years ranged from 1983 (*n* = 1) to 2020 (*n* = 45). The number of studies regarding women's health has increased exponentially over time (model *R*^2^ = 0.80, Figure 2). Nearly all studies were published in English (*n* = 350, 99.2%); the remainder were published in Spanish (2) or Turkish (1). Almost half of the studies were conducted (at least in part) in Europe, while 21.7% were conducted in North America; only one study was conducted in Africa (Figure 3).

**Figure 4:**
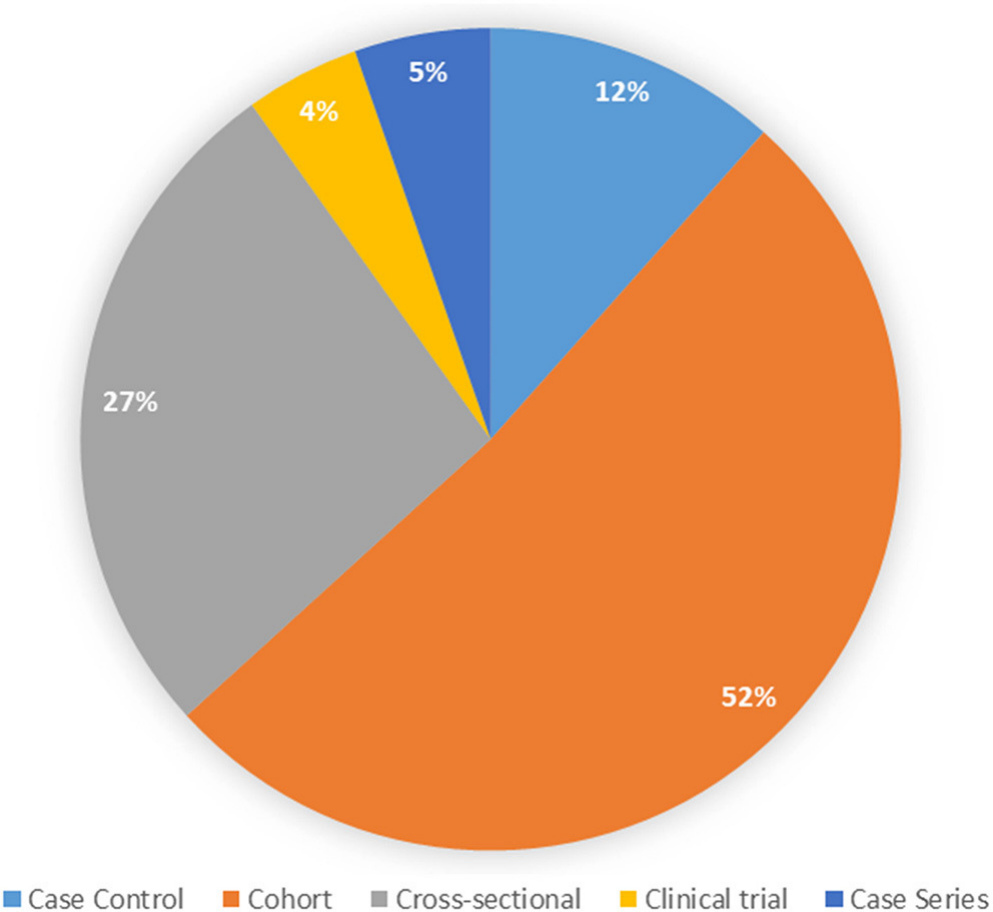
Types of study designs.

The most common design observed was a cohort study (51.4%), followed by cross-sectional studies (26.8%, Figure 4). Slightly more than half of the studies included a prospective component (*n* = 209, 57.3%); some studies included prospective and retrospective components. With respect to data sources used, the most common was primary data collection (*n* = 255, 66.2%), followed by use of a clinical registry (*n* = 63, 16.4%), medical records review (*n* = 40, 10.4%), and use of administrative data (*n* = 26, 6.7%); some studies used more than one data source.

### Study Populations

Overall, 278 (78%) publications for which data were extracted focused exclusively on women with MS. An additional 15 (4%) focused on children born to parents with MS, and 39 (11%) also included men or those with non-binary gender identities with MS. The remaining 22 (6%) articles studied MS as well as least one other disease including NMOSD, epilepsy, spinal cord injury, and headache.

Study sample sizes ranged from 2 to 96,937. The mean (SD) sample size was 466 (920) with a median of 130. Participants ranged in age from 12 to 81 years.

**Figure 5:**
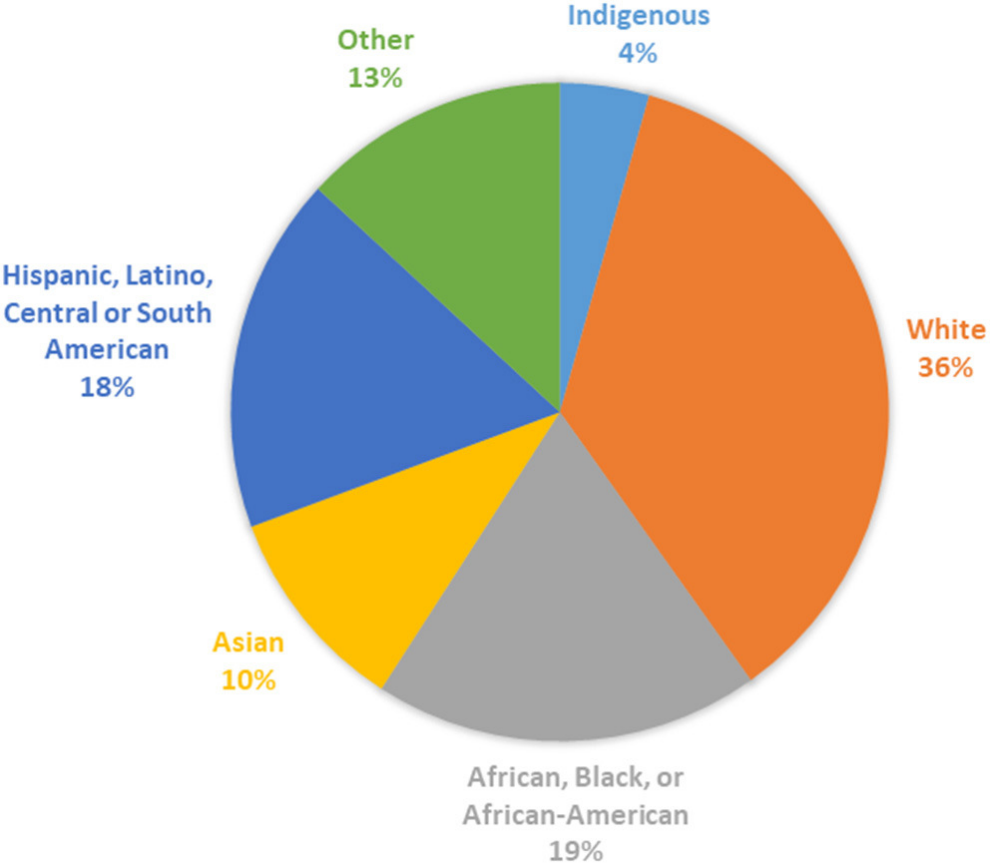
Racial and ethnic categories reported by study where reported (*n* = 45 studies).

Most studies did not report the race or ethnicity of their participants (*n* = 308, 87.2%); the races and ethnicities captured among reporting studies is shown in Figure 5. The proportion of studies that reported race and ethnicity was higher in North America (38.4%) than in all other regions (Australia/New Zealand: 20%, South America: 5.9%, Europe 5.7%, Asia 1.9%). Whereas, reporting of race and ethnicity appeared to demonstrate diversity at the study level, within North America, where reporting of race and ethnicity was most common, we found that 90% of participants enrolled were White. While numbers or percentages of White participants were given in all studies reporting race or ethnicity, reporting for all other groups was varied. Often race and ethnicity reporting included “other” or “unknown categories.” As a result, assessment of true participation from most racial and ethnic groups was difficult to accurately determine. Notably, there were 6 participants clearly identified as Native or Indigenous persons and 148 Asian persons in comparison to 29,812 clearly identified White participants.

Overall, 143 (39.7%) studies did not report the criteria used to establish the diagnosis of MS in study participants. Only a small number of the studies that did not report the diagnostic criteria used had exclusively employed administrative data sources (*n* = 10) where such information is typically unavailable.

Most studies reported the subtype of MS that were enrolled (*n* = 334, 94.6%). These studies frequently included participants with multiple subtypes of MS thus the number of subtypes reported was 543. At the study level, participants most often had relapsing remitting MS (RRMS) (*n* = 215, 39.6%), followed by secondary progressive MS (SPMS) (*n* = 88, 16.2%) and primary progressive MS (PPMS) (*n* = 75, 13.8%).

### Study Topics

**Figure 6:**
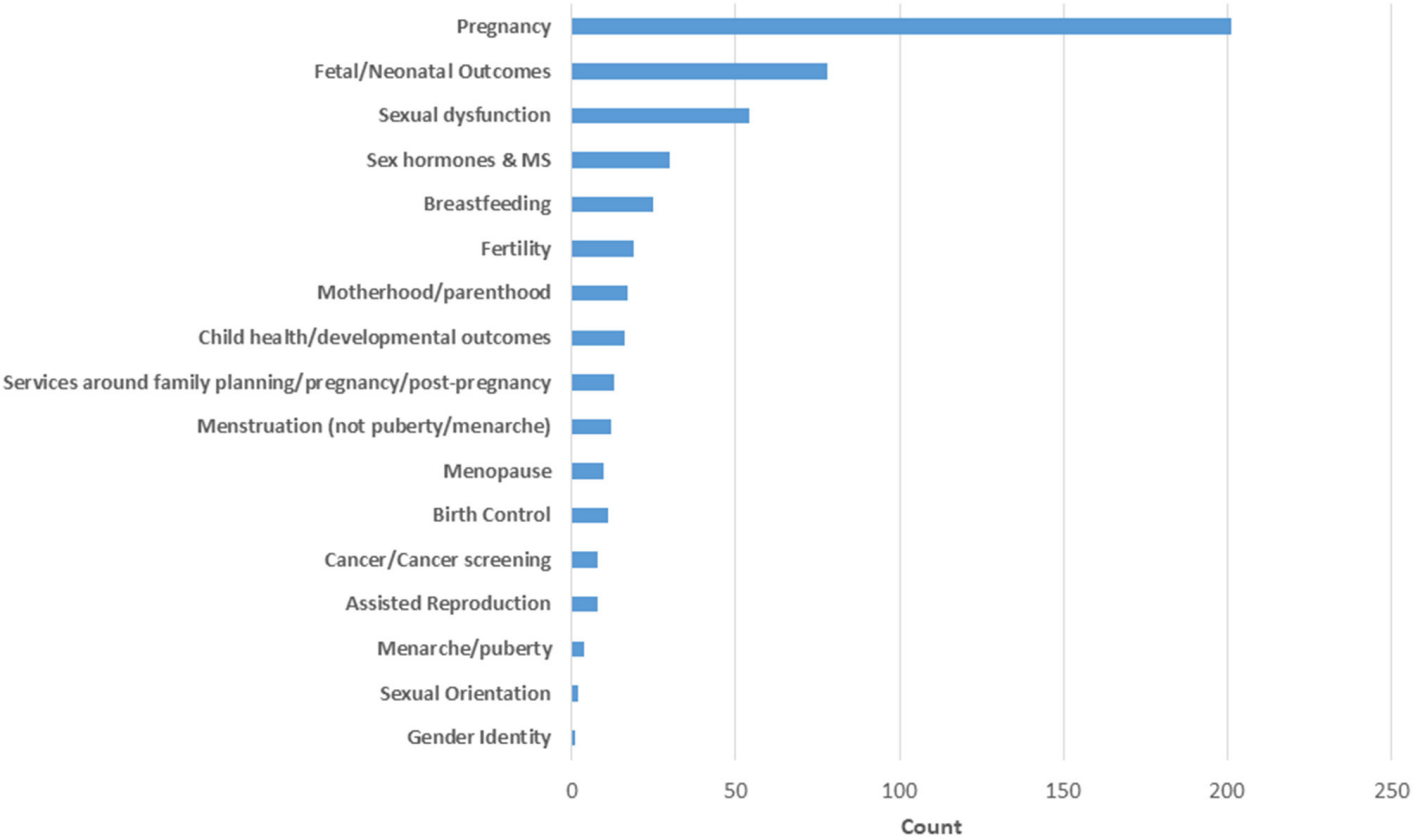
Frequency of topics studied.

Among the 353 studies, 509 topics were reported as some studies addressed more than one topic. Over one-third of these focused on pregnancy (*n* = 201, 37.2%) (Figure 6). Studies of pregnancy often focused on more than one aspect, the most common subtopics of interest being relapses (24.1%), pregnancy complications (23.1%), and disease-modifying therapy exposure (23.1%). Disability outcomes (14.2%) and mechanisms of pregnancy-related reductions in disease activity (8%) received less attention. The second most common topic was fetal/neonatal outcomes (14.4%) which is closely related to the topic of pregnancy, and sexual dysfunction was the third most common topic (10%). Most other topics, including menopause, birth control, assisted reproduction, cancer screening, and gender identity, received little attention (Figure 6).

Among studies that enrolled individuals with progressive MS, pregnancy, fetal/neonatal outcomes and sexual dysfunction were the most common topics, similar in overall frequency. Among the 201 studies that focused on pregnancy, only 51 (25.4%) studies included participants with progressive MS. Collectively, the studies focused on pregnancy that reported number of women and not simply number of pregnancies included 155,295 women, of whom only 1,316 (0.85%) were reported to be women with progressive MS. After excluding women for whom the subtype was not recorded, including from one large administrative data study (*n* = 96,937), 5.3% of women included in pregnancy studies had progressive MS.

**Figure 7:**
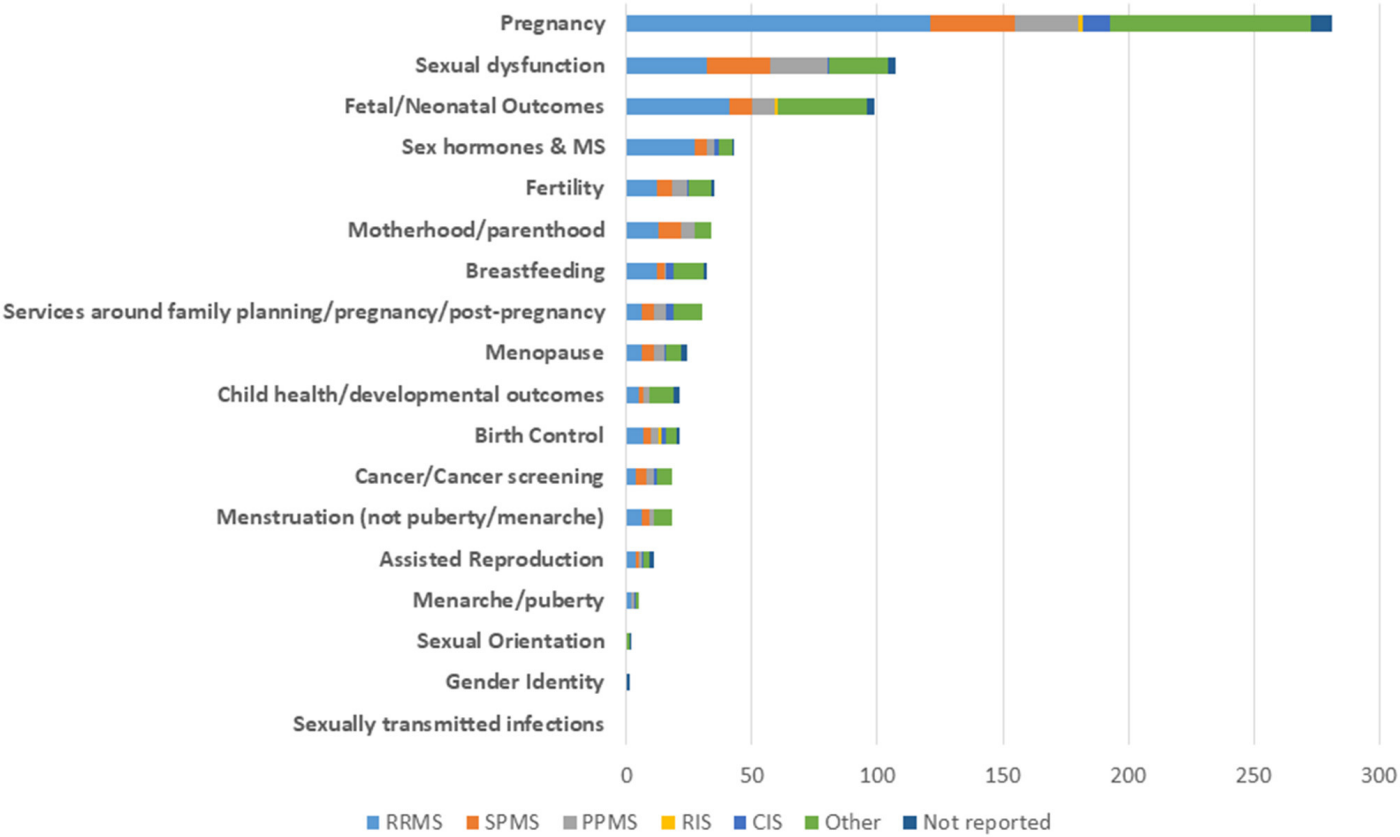
Frequency of study topics according to type of multiple sclerosis.

Ten studies assessed issues related to menopause. Of these, nine enrolled a total of 1,779 participants of whom 17% had SPMS, PPMS, or progressive relapsing MS (PRMS). The last study enrolled 148 participants and it was uncertain if they had RRMS or SPMS. Figure 7 details inclusion at the study level of participants with each disease subtype for all topic areas.

## Discussion

In this scoping review of women's health research in MS we reviewed 353 studies fulfilling our inclusion criteria. We found an increase in the focus on women's health research in MS over time but with variable coverage across different topics; by geographic location, racial, and ethnic group; and by MS subtype. Nonetheless, our findings also highlight important gaps.

We found limited coverage of women's health issues in MS by year until around the turn of the millennium. In 2004 for the first time the number of studies relating to women's health issues in MS reached 10; this rose sharply thereafter until 2020, where 45 studies on women's health issues in MS were identified in a single year. This trend is consistent with recent updates specifically focused on the women's health information available to treating clinicians and persons with MS. The American College of Obstetricians and Gynecologists (16), Multiple Sclerosis Society of Canada (17), and National Multiple Sclerosis Society (18) have all issued updates specifically focused on women's health in MS and MS Australia has become a sponsoring partner of a national Women's Health Week (19).

The current literature landscape is dominated by pregnancy-related studies with one-third of reviewed studies focusing on pregnancy and one in seven focusing on fetal/neonatal outcomes. Perhaps this observation is unsurprising given the pivotal social importance of pregnancy in a woman's life and the established intersection with the healthcare system for pregnancy (20). Additionally, the typical onset of MS during childbearing years (11), disproportionate likelihood to affect women (4, 5), and changes in disease course during and after pregnancy (21) lead to a wide range questions to researchers surrounding the topic. Correspondingly, we saw that all pregnancy subtopics of interest received attention with more than 10 studies touching on each subtopic. Despite this attention, gaps remain surrounding pregnancy for women with MS. Particularly, consistent with the lack of inclusion of pregnant women in clinical trials in general (22), pregnant women with MS were excluded from the recent clinical trials of new disease-modifying therapies (DMTs) (23, 24). Further, observational information surrounding use of DMTs approved in the period 2017 to date in pregnancy, including ocrelizumab, ofatumumab, cladribine, diroximel fumarate, siponimod, and other spingosine-1-phosphate receptor modulators (ozanimod, ponesimod) remains limited. The exclusion of pregnant and breastfeeding women from clinical trials, while customary in situations where there are prominent concerns for teratogenicity, continues to limit the type of evidence and timing of available information to clinicians and persons with MS surrounding pregnancy and breastfeeding.

Conversely, given that women with MS have a long life expectancy, menopause—a health topic that all women encounter if they live long enough—has received surprisingly little attention, with just 10 studies identified covering this large topic area. While menopause tends to hold less perceived social importance, 94% of women in the general population experience menopause symptoms, and 63% of those women feel that their symptoms would benefit from medical care (25). However only three studies were identified relating to menopause and symptoms in women with MS. Moreover, despite the growing recognition of the importance of hormonal influences in MS, the impact of menopause, use of hormone replacement therapies, as well as hormonal therapies unrelated to menopause on relapses, disability, and MRI outcomes in MS remains understudied (26, 27). There remains an opportunity to further the understanding of interactions of MS and menopause to help guide women with MS and provide additional insights about hormonal influences in MS at large.

Many other women's health topics also received little attention over the review period with nine topic areas having fewer than 15 relevant studies. While all these topics merit additional attention, we will briefly note three here. First, many women with MS are encouraged to use contraception because numerous DMTs and symptomatic agents potentially pose fetal harm. Additionally, 48% of reproductive age women worldwide use contraception (28). Yet, only 10 studies were identified evaluating birth control in women with MS. Second, more than 5 million children have been born following assisted reproduction (29), including up to 5% of all children in some countries (30). However, persons with MS and clinicians must make decisions about the growing availability of assisted reproduction with limited data. Third, there is a rising incidence of breast cancer globally (31), and cancers are of increasing concern in MS as cancer risks may increase during treatment with strong immunosuppressive therapies are used (32). However, relatively little attention has been focused on addressing disparities and barriers related to screening for breast and cervical cancers, which are recognized in the general population (33) as well as those related to physical impairments. It is important to understand more about how persons with MS are affected by breast and gynecological cancer as well as their participation and experience with screening.

In the last decade there have been changes reported in the demographics of persons with MS worldwide (34–36). There is known clinical variability in MS across racial and ethnic groups including differences in age of onset of disease, environmental and genetic risk factors, disease severity and progression, and health outcomes (37). Additionally, there is established variability in women's health issues by race and ethnicity including in age of pubertal onset, outcomes with assisted reproduction, perimenopausal symptoms, hormonal changes through menopause (38), and incidence and time to diagnosis and treatment of cervical cancer (39). Despite the importance of understanding the effects of race and ethnicity on health outcomes, the generalizability of the current literature across diverse racial and ethnic groups remains largely unknown since 87% of studies did not report these data. Most studies reporting racial and ethnic participant data were conducted in North America and it is unlikely that this generalizes globally given geographic differences in racial and ethnic distribution. Even within North America study findings cannot be well-extrapolated beyond the community self-identifying as White given that 9 out of 10 participants in reporting studies identified as White. This mirrors the inadequate representation of minority racial and ethnic groups reported in recent clinical trials of DMT (23, 40–42). Race and ethnicity are social constructs without biologic meaning, but they intersect with social determinants of health and inequities associated with health outcomes. It is imperative that the field reports the racial and ethnic characteristics of study participants, and addresses barriers to enrollment of underrepresented groups so that we can understand differences across these groups, clarify generalizability, and reduce health disparities.

Another threat to generalizability is the limited number of studies including participants from outside of North America and Europe; only one study included participants from Africa. Women's health issues vary by geographic region. For example, the incidence of breast cancer in Australia and New Zealand is 94 cases per 1,000,000 female population but is substantially lower, at 26 cases per 100,000 female population, in Middle Africa (43). Furthermore, the evaluation and treatment of MS differs globally. One illustration of these differences is that 82% of high income countries reported patients having access to natalizumab whereas only 10% of lower middle income and 0% of low income countries reported patients having access to natalizumab (44). The field needs further studies with participants outside of North America and Europe to help understand potential regional differences in women's health issues in MS, and to support the development of health care policies and services that meet the needs in each region.

Most studies focused on relapsing remitting MS. Studies that included participants with progressive MS were clustered predominantly in two topics, pregnancy and sexual dysfunction. Even among the studies that focused on menopause, which typically occurs around age 50 years, by which age when a higher proportion of women with MS will have progressive MS than at earlier ages, fewer than one in five participants enrolled had progressive MS. Additional focus on the women's health issues for patients with progressive MS is needed.

The field has also largely not examined women's health issues among women with MS who are lesbian, bisexual, and/or transgender. In the general population, lesbians are known to be less likely to receive cancer preventive services (45, 46) and transgender women are at higher risk of sexually transmitted infections (47). Given the potential additional risks of malignancy and infections with some DMTs (32), understanding how these and other women's health issues affect persons with MS who have a non-binary gender identities and those with non-heterosexual orientation is vital to adequately advise these individuals.

Our review has several limitations. First, although scoping reviews typically include a broader spectrum of evidence such as from electronic databases and gray literature (that is, information produced outside traditional publishing routes), we limited this search to electronic databases and peer-reviewed journal articles given the enormity of the search conducted. Next, we did not include single case reports which might be informative for issues such as adverse effects related to drug exposure in pregnancy, since our goal was to broadly identify research gaps. Articles published prior to 1980 were not reviewed, as our focus was to evaluate the scope of contemporary knowledge of women's health issues for persons with MS. Additionally, while animal and *in vitro* studies may provide important insights into mechanistic issues underlying women's health concerns these were not reviewed as we aimed to delineate the landscape of clinical knowledge as presently established in persons with MS. Finally, there are other issues relevant to women living with MS that we did not consider in our review such as domestic violence particularly in the context of women with physical or cognitive impairments.

Although the number of studies regarding women's health in MS has increased exponentially over time, greater than one-third of these studies focused on pregnancy. This review also identifies important knowledge gaps related to women's health in MS. Future studies are needed that focus more on understudied topics such as menopause, sex hormones and cancer screening. All studies addressing women's health should seek to include participants with a broader range of races and ethnicities, with progressive MS, and to clearly report these participant characteristics. Studies are also needed that include individuals with MS living in Asia-Pacific and African regions.

## Data Availability Statement

The original contributions presented in the study are included in the article/Supplementary Material, further inquiries can be directed to the corresponding author.

## Author Contributions

LR, HN, JO'M, MA, JC, KH, MT, SV, and RM conceived of the study, developed the protocol, and selected studies for review. MH developed the search strategies and conducted the search. LR, HN, JO'M, and RM extracted the study data. LR and RM drafted the manuscript. LR, HN, JO'M, MA, JC, KH, MT, SV, MH, and RM revised the manuscript and approved the final version. All authors contributed to the article and approved the submitted version.

## Funding

LR was supported by a Sylvia Lawry Fellowship Grant ID#: FP-190634172 from the National MS Society. RM was supported by the Waugh Family Chair in Multiple Sclerosis and by Research Manitoba. The International Advisory Committee on Clinical Trials in MS is sponsored by the European Committee for Treatments and Research in MS and the National Multiple Sclerosis Society.

## Conflict of Interest

LR received funding from the National MS Society Sylvia Lawry Physician Fellowship. HN receives funding from the Multiple Sclerosis Society of Canada's end MS Post-doctoral Fellowship, and the Michael Smith Foundation for Health Research Trainee Award. MA served on Scientific Advisory Boards for Biogen, Novartis, Roche, Merck, Sanofi Genzyme and Teva; received speaker honoraria from Biogen, Merck, Sanofi Genzyme, Roche, Novartis and Teva; received research grants for her Institution from Biogen, Merck, Sanofi Genzyme, Novartis and Roche. She is co-editor of the Multiple Sclerosis Journal. JC received personal compensation for consulting for Biogen, Bristol-Myers Squibb, Convelo, Genentech, Janssen, NervGen, Novartis, and PSI; speaking for H3 Communications; and serving as an Editor of *Multiple Sclerosis Journal*. KH received personal compensation as a speaker for Biogen, Bristol-Myers Squibb, Roche, Teva, Novartis, Bayer, Sanofi-Genzyme and Merck. She receives currently funding by the Innovationsfonds, the national MS society, Biogen, Teva, Novartis, Roche, Sanofi-Genzyme and Merck. MT has received compensation for consulting services, speaking honoraria and research support from Almirall, Bayer Schering Pharma, Biogen-Idec, Genzyme, Janssen, Merck-Serono, Novartis, Roche, Sanofi-Aventis, Viela Bio and Teva Pharmaceuticals. SV received consulting and lecture fees, travel grants and research support from Biogen, Celgène/BMS, Novartis, Merck, Roche, Sanofi Genzyme and Teva Pharma. RM receives research funding from CIHR, the MS Society of Canada, the National MS Society, the Consortium of MS Centers, the US Department of Defense, Research Manitoba and Crohn's and Colitis Canada. She was supported by the Waugh Family Chair in Multiple Sclerosis. She is a co-investigator on studies funded by Biogen Idec and Roche. The remaining authors declare that the research was conducted in the absence of any commercial or financial relationships that could be construed as a potential conflict of interest.

## Publisher's Note

All claims expressed in this article are solely those of the authors and do not necessarily represent those of their affiliated organizations, or those of the publisher, the editors and the reviewers. Any product that may be evaluated in this article, or claim that may be made by its manufacturer, is not guaranteed or endorsed by the publisher.

## Supplementary Material

Appendix I Search strategies. Women's health and multiple sclerosis (ovid medline)-1980-2021.

Appendix II Data extraction [standard].

Appendix III Characteristics of studies included in the scoping review.
