# Supplementary material for: Women's Health in Multiple Sclerosis: A Scoping Review
Source: Front Neurol. 2022 Jan 31;12:812147. doi: 10.3389/fneur.2021.812147 (PMC8841798; doi:10.3389/fneur.2021.812147)
Supplement: Appendix I — Search strategies. Women's health and multiple sclerosis (ovid medline)-1980-2021. [file Table_1.docx]

**Search Strategies**
 **Women’s Health and Multiple Sclerosis (Ovid Medline)-1980-2021**

1. multiple sclerosis.mp. or exp Multiple Sclerosis/ or MS.mp

2. ((disseminated or insular or multiplex or multiple) adj2 sclerosis).mp

| 3. optic neuritis.mp. or exp Optic Neuritis/ |  |
| --- | --- |
| 4. transverse myelitis.mp. or exp Myelitis, Transverse/ |  |
| 5. "clinically isolated syndrome".mp 6. "radiologically isolated syndrome".mp.  7. exp women's health/ or exp maternal health/  8. exp Reproductive Health/  9. exp Sexual Health/ 10. exp fertility/ or exp ovarian reserve/ or exp time-to-pregnancy/  11. exp reproductive physiological phenomena/ or exp cell lineage/ or exp climacteric/ or exp andropause/ or exp menopause/ or exp menopause, premature/ or exp perimenopause/ or exp postmenopause/ or exp premenopause/ or exp clutch size/ or exp estrous cycle/ or exp anestrus/ or exp diestrus/ or exp estrus/ or exp estrus synchronization/ or exp metestrus/ or exp proestrus/ or exp fertility/ or exp ovarian reserve/ or exp time-to-pregnancy/ or exp fetal viability/ or exp fetal weight/ or exp follicular atresia/ or exp gestational age/ or exp gestational weight gain/ or exp gravidity/ or exp labor presentation/ or exp breech presentation/ or lactation/ or exp milk ejection/ or exp lacteal elimination/ or exp litter size/ or exp maternal age/ or exp menstrual cycle/ or exp fertile period/ or exp follicular phase/ or exp luteal phase/ or exp menstruation/ or exp oviparity/ or exp ovoviviparity/ or exp parity/ or exp paternal age/ or exp peripartum period/ or exp postpartum period/ or exp lactation/ or exp pregnancy rate/ or exp pregnancy trimesters/ or exp pregnancy trimester, first/ or exp pregnancy trimester, second/ or exp pregnancy trimester, third/ or exp puberty/ or exp adrenarche/ or exp menarche/ or exp reproduction/ or exp coitus/ or exp ejaculation/ or exp "embryonic and fetal development"/ or exp embryonic development/ or exp embryo implantation/ or exp embryo implantation, delayed/ or exp twinning, embryonic/ or exp twinning, dizygotic/ or exp twinning, monozygotic/ or exp embryonic induction/ or exp fetal development/ or exp fetal movement/ or exp fetal organ maturity/ or exp organogenesis/ or exp neurogenesis/ or exp neuronal outgrowth/ or exp axon fasciculation/ or exp axon guidance/ or exp fertilization/ or exp ovum transport/ or exp self-fertilization/ or exp sperm capacitation/ or exp sperm-ovum interactions/ or exp acrosome reaction/ or exp sperm transport/ or exp gametogenesis/ or exp gametogenesis, plant/ or exp oogenesis/ or exp vitellogenesis/ or exp spermatogenesis/ or exp sperm maturation/ or exp insemination/ or exp insemination, artificial/ or exp insemination, artificial, heterologous/ or exp insemination, artificial, homologous/ or exp orgasm/ or exp oviposition/ or exp ovulation/ or exp anovulation/ or exp luteinization/ or exp corpus luteum maintenance/ or exp luteolysis/ or exp ovulation inhibition/ or exp superovulation/ or exp penile erection/ or exp pollination/ or exp pregnancy/ or exp labor, obstetric/ or exp cervical ripening/ or exp labor onset/ or exp labor stage, first/ or exp labor stage, second/ or exp labor stage, third/ or exp "trial of labor"/ or exp uterine contraction/ or exp maternal-fetal exchange/ or exp parturition/ or exp birth setting/ or exp home childbirth/ or exp natural childbirth/ or exp term birth/ or exp placentation/ or exp pregnancy in adolescence/ or exp pregnancy outcome/ or exp abortion, spontaneous/ or exp live birth/ or exp stillbirth/ or exp pregnancy, high-risk/ or exp pregnancy maintenance/ or exp pregnancy, multiple/ or exp pregnancy, quadruplet/ or exp pregnancy, quintuplet/ or exp pregnancy, triplet/ or exp pregnancy, twin/ or exp superfetation/ or exp pregnancy, unplanned/ or exp pregnancy, unwanted/ or exp prenatal nutritional physiological phenomena/ or exp pseudopregnancy/ or exp reproduction, asexual/ or exp parthenogenesis/ or exp apomixis/ or exp reproductive behavior/ or exp contraception behavior/  12. exp infertility/ or exp infertility, female/  13. sterility.mp. or Infertility/  14. (barren or childlessness).mp.  15. reproductive techniques/ or exp contraception/ or exp coitus interruptus/ or exp contraception, barrier/ or exp contraception, immunologic/ or exp contraception, postcoital/ or exp contraceptive effectiveness/ or exp hormonal contraception/ or exp long-acting reversible contraception/ or exp natural family planning methods/ or exp ovulation inhibition/ or exp sterilization, reproductive/ or exp fallopian tube patency tests/ or exp ovulation detection/ or exp ovulation prediction/ or exp reproductive techniques, assisted/ or exp donor conception/ or exp embryo transfer/ or exp single embryo transfer/ or exp fertility preservation/ or exp fertilization in vitro/ or exp mitochondrial replacement therapy/ or exp sperm injections, intracytoplasmic/ or exp gamete intrafallopian transfer/ or exp in vitro oocyte maturation techniques/ or exp insemination, artificial/ or exp insemination, artificial, heterologous/ or exp insemination, artificial, homologous/ or exp oocyte donation/ or exp oocyte retrieval/ or exp ovulation induction/ or exp superovulation/ or exp posthumous conception/ or exp sperm retrieval/ or exp zygote intrafallopian transfer/ or exp tocolysis/  16. hormonal stimulation.mp.  17. intrauterine insemination.mp.  18. birth control.mp.  19. tubal ligation.mp. or exp Sterilization, Tubal/  20. exp contraceptive devices, female/ or exp condoms, female/ or exp intrauterine devices/ or exp intrauterine devices, medicated/ or exp intrauterine devices, copper/ or exp contraceptive devices, male/ or exp condoms/  21. exp contraceptive agents, female/ or exp contraceptives, oral/ or exp contraceptives, oral, combined/ or exp ethinyl estradiol-norgestrel combination/ or exp contraceptives, oral, hormonal/ or exp algestone/ or exp chlormadinone acetate/ or exp desogestrel/ or exp dimethisterone/ or exp ethinyl estradiol/ or exp ethynodiol diacetate/ or exp gestrinone/ or exp lynestrenol/ or exp medroxyprogesterone/ or exp megestrol/ or exp mestranol/ or exp norethindrone/ or exp norethynodrel/ or exp norgestrel/ or exp contraceptives, oral, sequential/ or exp contraceptives, oral, synthetic/ or exp algestone acetophenide/ or exp levonorgestrel/ or exp mifepristone/ or exp norgestrienone/ or exp contraceptives, postcoital/ or exp contraceptives, postcoital, hormonal/ or exp contraceptives, postcoital, synthetic/ or exp centchroman/ or exp luteolytic agents/ or exp cloprostenol/ or exp triptorelin pamoate/ or exp menstruation-inducing agents/ or exp sperm immobilizing agents/ or exp spermatocidal agents/ or exp nonoxynol/ or exp octoxynol/ or exp contraceptive agents, hormonal/ or exp medroxyprogesterone acetate/ or exp norethindrone acetate/ or exp contraceptive agents, male/  22. exp pregnancy/ or exp gravidity/ or exp labor, obstetric/ or exp cervical ripening/ or exp labor onset/ or exp labor presentation/ or exp "trial of labor"/ or exp uterine contraction/ or exp maternal-fetal exchange/ or exp parity/ or exp parturition/ or exp birth setting/ or exp home childbirth/ or exp natural childbirth/ or exp term birth/ or exp placentation/ or exp pregnancy in adolescence/ or exp pregnancy outcome/ or exp abortion, spontaneous/ or exp live birth/ or exp stillbirth/ or exp pregnancy, high-risk/ or exp pregnancy maintenance/ or exp corpus luteum maintenance/ or exp pregnancy, multiple/ or exp pregnancy, unplanned/ or exp pregnancy, unwanted/ or exp prenatal nutritional physiological phenomena/ or exp pseudopregnancy/ 23. exp hypertension, pregnancy-induced/ or exp eclampsia/ or exp hellp syndrome/ or exp pre-eclampsia/  24. Prenatal Care/ or maternal-fetal health.mp.  25. (peripartum adj2 (relapse or exacerbation or attack)).mp.  26. pregnancy registry.mp.  27. exp Pregnancy Outcome/  28. abortion, spontaneous/ or abortion, threatened/ or fetal death/ or fetal resorption/ or stillbirth/ or exp obstetric labor, premature/ or exp premature birth/  29. exp delivery, obstetric/ or cesarean section/ or extraction, obstetrical/ or vacuum extraction, obstetrical/ or labor, induced/ or vaginal birth after cesarean/ 30. cesarean section/ or extraction, obstetrical/ or labor, induced/ or vaginal birth after cesarean/  31. Infant, Newborn/  32. exp infant, newborn/ or infant, low birth weight/ or infant, small for gestational age/ or infant, very low birth weight/ or infant, extremely low birth weight/ or infant, postmature/ or infant, premature/ or infant, extremely premature/  33. exp Fetal Death/  34. exp Teratogens/  35. exp Teratogenesis/  36. exp Congenital Abnormalities/ 37. Mood Disorders/ or exp Depression, Postpartum/ or exp Postpartum Period/ or exp Peripartum Period/ or peripartum mood disorders.mp. or exp Puerperal Disorders/  38. adolescent development/ or exp child development/ or exp language development/ or exp neurobehavioral manifestations/  39. "attention deficit and disruptive behavior disorders"/ or attention deficit disorder with hyperactivity/ or conduct disorder/ or child development disorders, pervasive/ or autism spectrum disorder/ or asperger syndrome/ or autistic disorder/ or motor skills disorders/  40. exp motor disorders/ or exp neurocognitive disorders/ or "attention deficit and disruptive behavior disorders"/ or child behavior disorders/ or child development disorders, pervasive/ or developmental disabilities/ or learning disabilities/ or motor skills disorders/  41. exp prenatal diagnosis/ or exp amniocentesis/ or exp chorionic villi sampling/ or exp fetoscopy/ or exp maternal serum screening tests/ or exp noninvasive prenatal testing/ or exp ultrasonography, prenatal/ or exp cervical length measurement/ or exp nuchal translucency measurement/  42. exp Genetic Counseling/ or reproductive counseling.mp.  43. exp Preconception Care/ or exp Family Planning Services 44. exp family leave/ or exp parental leave 45. exp Return to Work/  46. exp Employment/  47. Child Care/  48. breast-feeding.mp. or exp Breast Feeding/  49. lactation.mp. or exp Lactation Disorders/ or exp Lactation/  50. exp Sex Reassignment Surgery/ or exp Gender Identity/ or exp Transsexualism/ or exp Transgender Persons/ or transgender health.mp. or exp Gender Dysphoria/  51. Intersex Persons/  52. intergender.mp.  53. sexual inversion.mp. 54.(bigender or gender fluid or gender variant or transman or transwoman).mp. 55. exp "Sexual and Gender Minorities"/  56. (gender adj2 (nonconforming or diverse or questioning or queer)).mp.  57. third sex.mp.  58. dual gender.mp.  59. exp Homosexuality, Female/ or exp Homosexuality/ or lgbt.mp. or exp Bisexuality/  60. lesbian.mp.  61. exp menopause/ or menopause, premature/ or perimenopause/ or postmenopause/ or premenopause/  62. hormone replacement therapy.mp. or exp Hormone Replacement Therapy/ or hrt.mp.  63. exp Estrogen Replacement Therapy/ 64. (progesterone or progestin).mp.  65. antimullerian hormone.mp. or exp Anti-Mullerian Hormone 66. hot flashes.mp. or exp Hot Flashes/  67. exp Vasomotor System/ or vasomotor symptoms.mp.  68. exp Dyspareunia/ or vaginal dryness.mp.  69. exp Hysterectomy, Vaginal/ or exp Hysterectomy/ or hysterectomy.mp.  70. oophorectomy.mp. or exp Ovariectomy/  71. mammography.mp. or exp Mammography/  72. pap smears.mp. or Papanicolaou Test/  73. exp Vaginal Smears/ or exp Papillomavirus Infections/ or exp Papillomaviridae/ or exp "Early Detection of Cancer"/ or hpv testing.mp. or exp Cervical Intraepithelial Neoplasia/  74. exp Breast Neoplasms/di [Diagnosis] 75. exp uterine neoplasms/ or endometrial neoplasms/ or uterine cervical neoplasms/  76. exp Genital Neoplasms, Female/di, dg [Diagnosis, Diagnostic Imaging]  77. or/1-6  78. or/7-76  79. 77 and 78  80. limit 79 to english language 81. limit 80 to yr="1980 – 2021”  **Women’s Health and Multiple Sclerosis -Ovid Embase -1980-2021**   \| 1. multiple sclerosis.mp. or exp multiple sclerosis/ or MS.mp. \|  \| \| --- \| --- \| \| 2. ((disseminated or insular or multiplex or multiple) adj2 sclerosis).mp. \|  \| \| 3. optic neuritis.mp. or exp optic neuritis/ \|  \| \| 4. Myelitis, Transverse.mp. or exp myelitis/ \|  \| \| 5. "clinically isolated syndrome".mp. or exp demyelinating disease/ \|  \| \| 6. "radiologically isolated syndrome".mp. \|  \| \| 7. exp women's health/ or exp maternal health/ \|  \| \| 8. Reproductive Health.mp. or exp reproductive health/ \|  \| \| 9. Sexual Health.mp. or exp sexual health/ \|  \| \| 10. exp female fertility/ \|  \| \| 11. exp ovarian reserve/ \|  \| \| 12. time-to-pregnancy.mp. or exp time to pregnancy/ \|  \| \| 13. exp reproduction/ or acrosome reaction/ or androgenesis/ or breeding/ or childbirth/ or conception/ or cross fertilization/ or decidualization/ or egg laying/ or egg production/ or extra pair paternity/ or fertilization/ or gynogenesis/ or hatching/ or insemination/ or nucleic autogamy/ or oviparity/ or ovoviviparity/ or paedogenesis/ or parity/ or pregnancy/ or prenatal development/ or progeny/ or pseudopregnancy/ or puerperium/ or reproductive behavior/ or reproductive interference/ or self fertilization/ or spawning/ or sperm competition/ or spermatozoon migration/ or spermatozoon penetration/ or thelytoky/ or viviparity/ \|  \| \| 14. exp infertility/ or female infertility/ or male infertility/ or subfertility/ \|  \| \| 15. exp female sterility/ or sterility.mp. or exp cytoplasmic male sterility/ or exp male sterility/ \|  \| \| 16. barren.mp. 1. multiple sclerosis.mp. or exp multiple sclerosis/ or MS.mp.  2. ((disseminated or insular or multiplex or multiple) adj2 sclerosis).mp.  3. optic neuritis.mp. or exp optic neuritis/  4. Myelitis, Transverse.mp. or exp myelitis/  5. "clinically isolated syndrome".mp. or exp demyelinating disease/  6. "radiologically isolated syndrome".mp.  7. exp women's health/ or exp maternal health/  8. Reproductive Health.mp. or exp reproductive health/  9. Sexual Health.mp. or exp sexual health/  10. exp female fertility/  11. exp ovarian reserve/  12. time-to-pregnancy.mp. or exp time to pregnancy/  13. exp reproduction/ or acrosome reaction/ or androgenesis/ or breeding/ or childbirth/ or conception/ or cross fertilization/ or decidualization/ or egg laying/ or egg production/ or extra pair paternity/ or fertilization/ or gynogenesis/ or hatching/ or insemination/ or nucleic autogamy/ or oviparity/ or ovoviviparity/ or paedogenesis/ or parity/ or pregnancy/ or prenatal development/ or progeny/ or pseudopregnancy/ or puerperium/ or reproductive behavior/ or reproductive interference/ or self fertilization/ or spawning/ or sperm competition/ or spermatozoon migration/ or spermatozoon penetration/ or thelytoky/ or viviparity/  14. exp infertility/ or female infertility/ or male infertility/ or subfertility/  15. exp female sterility/ or sterility.mp. or exp cytoplasmic male sterility/ or exp male sterility/  16. barren.mp.  17. childlessness.mp. or exp childlessness/ 18. exp reproductive procedure/ or exp animal reproductive procedures/  19. exp contraception/ or barrier contraception/ or emergency contraception/ or hormonal contraception/ or long-acting reversible contraception/ or oral contraception/ or ovulation inhibition/ or reproductive sterilization/ or vagina contraception/  20. exp infertility therapy/ or exp artificial insemination/ or exp embryo disposition/ or exp fertility preservation/ or exp gamete intrafallopian transfer/ or exp in vitro fertilization/ or exp oocyte donation/ or exp sperm retrieval/ or exp zygote intrafallopian transfer/  21. exp hormonal regulation/  22. exp intrauterine insemination/ or exp artificial insemination/  23. exp birth control/ or exp contraception/ or exp family planning/ or exp induced abortion/ or exp involuntary fertility control/  24. exp uterine tube sterilization/ or exp female sterilization/  25. exp contraceptive device/  26. exp contraceptive agent/ or exp hormonal contraceptive agent/ or exp injectable contraceptive agent/ or exp luteolytic agent/ or exp male contraceptive agent/ or exp menstruation inducing agent/ or exp oral contraceptive agent/ or exp postcoitus contraceptive agent/ or exp spermicidal agent/  27. exp pregnancy/ or exp adolescent pregnancy/ or exp first trimester pregnancy/ or exp mother fetus relationship/ or exp multiple pregnancy/ or exp second trimester pregnancy/ or exp third trimester pregnancy/ or exp unplanned pregnancy/ or exp unwanted pregnancy/  28. parity/  29. exp birth/   \| 30. exp birth setting/ \|  \| \| --- \| --- \| \| 31. exp obstetric delivery/ or exp fetal version/ or exp home delivery/ or exp instrumental delivery/ or exp labor induction/ or exp labor management/ or exp labor support/ or exp natural childbirth/ or exp obstetric anesthesia/ or exp placental delivery/ or exp tocolysis/ or exp vaginal birth after cesarean/ or exp vaginal delivery/ or exp water birth/ \|  \| \| 32. exp maternal hypertension/ \|  \| \| 33. exp preeclampsia/ or exp "eclampsia and preeclampsia"/ \|  \| \| 34. exp obstetric procedure/ or exp intrapartum care/ or exp nurse midwifery/ or exp obstetric delivery/ or exp obstetric operation/ or exp perinatal care/ or exp postnatal care/ or exp prenatal care/ or exp prepregnancy care/ \|  \| \| 35. maternal-fetal health.mp. \|  \| \| 36. (peripartum adj2 (relapse or exacerbation or attack)).mp. [mp=title, abstract, heading word, drug trade name, original title, device manufacturer, drug manufacturer, device trade name, keyword, floating subheading word, candidate term word] \|  \| \| 37. pregnancy registry.mp. \|  \| \| 38. exp pregnancy outcome/ \|  \| \| 39. abortion/ or exp blighted ovum/ or exp fetus wastage/ or exp hormonal abortion/ or exp imminent abortion/ or exp incomplete abortion/ or exp missed abortion/ or exp recurrent abortion/ or exp second trimester abortion/ or exp septic abortion/ or exp spontaneous abortion/ or exp "spontaneous abortion of partner"/ \|  \| \| 40. exp prematurity/ or "immature and premature labor"/ or newborn disease/ \|  \| \| 41. exp fetus death/ or exp fetus resorption/ or exp stillbirth/ \|  \| \| 42. exp premature labor/ \|  \| \| 43. exp cesarean section/ \|  \| \| 44. exp vacuum extraction/ or exp cesarean section/ or obstetrical extraction.mp. or exp vaginal delivery/ \|  \| \| 45. exp infant/ or baby/ or high risk infant/ or hospitalized infant/ or newborn/ \|  \| \| 46. exp teratogenicity/   \|  \| \| \| --- \| --- \| \| 47. exp teratogenesis/ or exp chemical teratogenesis/ \| \|  \| \| 48. exp newborn disease/ or infant disease/ \| \|  \| \| 49. depression/ or exp perinatal depression/ \| \|  \| \| 50. exp postnatal depression/ \| \|  \| \| 51. exp pregnancy disorder/ or exp abortion/ or exp delayed implantation/ or exp ectopic pregnancy/ or exp embryopathy/ or exp fetomaternal transfusion/ or exp fetus disease/ or exp frigidity/ or exp high risk pregnancy/ or exp labor complication/ or exp morning sickness/ or exp multiple pregnancy/ or exp obstetric emergency/ or exp peripartum cardiomyopathy/ or exp placenta disorder/ or exp pregnancy complication/ or exp "pregnancy disorders of endocrine origin"/ or exp pregnancy toxemia/ or exp prolonged pregnancy/ or exp puerperal disorder/ or exp single umbilical artery/ or exp trophoblastic disease/ or exp umbilical malformation/ \| \|  \| \| 52. exp prenatal care/ or exp prenatal screening/ \| \|  \| \| 53. exp human development/ or adolescent development/ or language development/ or psychosocial development/ or self actualization/ or speech development/ \| \|  \| \| 54. exp child development/ \| \|  \| \| 55. exp behavior disorder/ or abnormal behavior/ or attention deficit disorder/ or communication disorder/ or disruptive behavior/ or impulse control disorder/ or psychomotor disorder/ or psychosocial disorder/ \| \|  \| \| 56. exp developmental disorder/ \| \|  \| \| 57. exp autism/ or exp asperger syndrome/ or exp childhood disintegrative disorder/ or exp "pervasive developmental disorder not otherwise specified"/ or exp rett syndrome/ \| \|  \| \| 58. exp motor dysfunction/ or exp ataxia/ or exp benign neonatal sleep myoclonus/ or exp contracture/ or exp coordination disorder/ or exp developmental coordination disorder/ or exp drop attack/ or exp experimental motor dysfunction/ or exp extrapyramidal symptom/ or exp gait disorder/ or exp hereditary motor sensory neuropathy/ or exp hyperactivity/ or exp hypoactivity/ or exp immobility/ or exp limited mobility/ or exp minimal brain dysfunction/ or exp motor retardation/ or exp muscle fatigue/ or exp muscle hypertonia/ or exp muscle hypotonia/ or exp muscle spasm/ or exp muscle weakness/ or exp periodic limb movement disorder/ or exp purine nucleoside phosphorylase deficiency/ or exp pyramidal sign/ or exp restlessness/ or exp rigor/ or exp tremor/ or exp unsteadiness/ or exp walking difficulty/ \| \|  \| \| 59. exp "disorders of higher cerebral function"/ \| \|  \| \| 60. exp prenatal diagnosis/ or exp amniocentesis/ or exp chorionic villi sampling/ or exp fetoscopy/ or exp maternal serum screening tests/ or exp noninvasive prenatal testing/ or exp ultrasonography, prenatal/ or exp cervical length measurement/ or exp nuchal translucency measurement/ \| \|  \| \| 61. exp Genetic Counseling/ or reproductive counseling.mp. \| \|  \| \| 62. exp prepregnancy care/ \| \|  \| \| 63. exp Family Planning Services/ \| \|  \| \| 64. exp family leave/ \| \| \| \|  \| \| 65. exp return to work/ \| \| \| \|  \| \| 66. exp child care/ \| \| \| \|  \| \| 67. infant feeding/ or exp breast feeding/ \| \| \| \|  \| \| 68. exp lactation inhibition/ or exp lactation/ or exp lactation disorder/ \| \| \| \|  \| \| 69. exp sex reassignment/ \| \| \| \|  \| \| 70. exp gender identity/ \| \| \| \|  \| \| 71. exp transgender/ or exp female to male transgender/ or exp male to female transgender/ \| \| \| \|  \| \| 72. exp gender dysphoria/ or transsexualism/ \| \| \| \|  \| \| 73. exp lgbtqia+ people/ or asexual people/ or intersex/ or lgbt people/ \| \| \| \|  \| \| 74. exp lgbt people/ or bisexual female/ or bisexual male/ or homosexual female/ or homosexual male/ or transgender/ \| \| \| \|  \| \| 75. intergender.mp. \| \| \| \|  \| \| 76. sexual inversion.mp. \| \| \| \|  \| \| 77. (bigender or gender fluid or gender variant or transman or transwoman).mp. \| \| \| \|  \| \|  \| \|  \| \|  \| \| 78. (gender adj2 (nonconforming or diverse or questioning or queer)).mp. \| \| \| \|  \| \| 79. (third sex or dual gender).mp. [mp=title, abstract, heading word, drug trade name, original title, device manufacturer, drug manufacturer, device trade name, keyword, floating subheading word, candidate term word] \| \| \| \|  \| \| 80. exp lesbianism/ \| \| \| \|  \| \| \| 81. exp sexual orientation/ or exp bisexuality/ or exp heterosexuality/ or exp homosexuality/ 82. exp "menopause and climacterium"/ or exp climacterium/ or exp early menopause/ or exp menopause/ or exp postmenopause/ or exp premenopause/. exp "menopause and climacterium"/ or exp climacterium/ or exp early menopause/ or exp menopause/ or exp postmenopause/ or exp premenopause/ \|  \| \| --- \| --- \| \|  \| \| 83. hormone replacement therapy.mp. or exp hormone substitution/ \|  \| \| 84. exp steroid therapy/ or androgen therapy/ or corticosteroid therapy/ or estrogen therapy/ \|  \| \| 85. (progesterone or progestin).mp. [mp=title, abstract, heading word, drug trade name, original title, device manufacturer, drug manufacturer, device trade name, keyword, floating subheading word, candidate term word] \|  \| \| 86. antimullerian hormone.mp. or exp Anti-Mullerian Hormone/ \|  \| \| 87. hot flashes.mp. or exp Hot Flashes/ \|  \| \| 88. exp Vasomotor System/ or vasomotor symptoms.mp. \|  \| \| 89. exp vasomotor disorder/ or exp hot flush/ or vasomotor symptoms.mp. or exp estradiol/ \|  \| \| 90. (Dyspareunia or vaginal dryness).mp. [mp=title, abstract, heading word, drug trade name, original title, device manufacturer, drug manufacturer, device trade name, keyword, floating subheading word, candidate term word] \|  \| \| 91. exp vaginal dryness/ \|  \| \| 92. exp hysterectomy/ \|  \| \| 93. exp ovariectomy/ \|  \| \| 94. breast examination/ or exp breast biopsy/ or exp breast self examination/ or exp mammography/ or exp nipple aspiration/ \|  \| \| 95. pap smears.mp. or exp Papanicolaou test/ \|  \| \| 96. female genital tract cytology/ \|  \| \| 97. exp papillomaviridae/ or exp papillomavirus infection. exp "menopause and climacterium"/ or exp climacterium/ or exp early menopause/ or exp menopause/ or exp postmenopause/ or exp premenopause/   \| 98. exp early cancer diagnosis/ \|  \| \| --- \| --- \| \| 99. hpv testing.mp. \|  \| \| 100. exp uterine cervix carcinoma in situ/ \|  \| \| 101. exp vagina smear/ \|  \| \| 102. exp breast tumor/di [Diagnosis] \|  \| \| 103. breast self examination/ or cancer screening/ \|  \| \| 104. or/1-6 \|  \| \| 105. or/7-106 \|  \| \| 106. limit 108 to yr="1980 -Current" \|  \| \|  \|  \| \|  \| \| \| \| \|  \| \|  \| \| \| \|  \| \|  \| \|  \| |  |
