# Supplementary material for: Women's Health in Multiple Sclerosis: A Scoping Review
Source: Front Neurol. 2022 Jan 31;12:812147. doi: 10.3389/fneur.2021.812147 (PMC8841798; doi:10.3389/fneur.2021.812147)
Supplement: Appendix II — Data extraction [standard]. [file Table_2.docx]

## Data Extraction [Standard]

- Publication Year
  - 1980
  - 1982
  - 1983
  - 1984
  - 1985
  - 1986
  - 1987
  - 1988
  - 1989
  - 1990
  - 1991
  - 1992
  - 1993
  - 1994
  - 1995
  - 1996
  - 1997
  - 1997
  - 1998
  - 1999
  - 2000
  - 2001
  - 2002
  - 2003
  - 2004
  - 2005
  - 2006
  - 2007
  - 2008
  - 2009
  - 2010
  - 2011
  - 2012
  - 2013
  - 2014
  - 2015
  - 2016
  - 2017
  - 2018
  - 2019
  - 2020
- Study language
  - English
  - French
  - Spanish
  - German
  - Italian
  - Polish
  - Greek
  - Russian
- Study region (all that apply)
  - North America
  - Central America/Caribeean
  - South America
  - Europe
  - Asia
  - Australia/New Zealand
  - Africa
  - Not reported
- Data Source
  - Administrative (health claims) data
  - Clinical registry
  - Primary data collection (not registry)
  - Not reported
  - Medical records review
- Study Design
  - Prospective or retrospective
    - Prospective
    - Retrospective
  - Type
    - Case Control
    - Cohort
    - Cross-sectional
    - Clinical trial
    - Case Series
  - Patient reported outcomes
    - Patient reported outcomes reported
    - Patient reported outcomes not reported
  - Number of women with RIS/CIS or MS enrolled
- Demyelinating Disease
  - RIS
  - CIS
  - RRMS
  - SPMS
  - PPMS
  - MS subtype unspecified
  - MS other subtype
  - Not reported
- Diagnostic criteria used
  - McDonald 2001
  - McDonald 2005
  - McDonald 2010
  - McDonald 2017
  - Poser
  - Schumacher
  - Other
  - Not reported
  - Self-reported
- Race/Ethnicity
  - Indigenous
  - White
  - African, Black, or African-American
  - Asian
  - Caribbean
  - Hispanic, Latino, Central or South American
  - Not reported
  - Other
- Age
  - Lowest age
  - Highest age
  - Mean age
  - Not reported
- Pregnant or not
  - Pregnant
  - Not pregnant
  - Not reported
- Disability level
  - Lowest EDSS
  - Highest EDSS
  - Not reported
  - Other disability measure
- Study Topic
  - Gender Identity
  - Sexual Orientation
  - Birth Control
  - Pregnancy
    - Pregnancy complications
    - MS relapses
    - Disability outcomes
    - MRI outcomes
    - DMT exposure
    - Anesthesia (generalized, regional, neuraxial)
    - Mechanisms
  - Breastfeeding
    - MS relapses
    - DMT exposure
    - MS disability outcomes
    - MRI outcomes
    - non-DMT drug exposure
  - Fertility
  - Assisted Reproduction
  - Fetal/Neonatal Outcomes
  - Menarche/puberty
  - Menstruation (not puberty/menarche)
  - Menopause
    - MS relapses
    - MS disability outcomes
    - MRI outcomes
    - Hormone replacement therapy
    - Symptoms
  - Child health/developmental outcomes
  - Cancer/Cancer screening
  - Sexually transmitted infections
  - Services around family planning/pregnancy/post-pregnancy
  - Sexual dysfunction
  - Motherhood/parenthood
  - Sex hormones & MS
