# Supplementary material for: Women's Health in Multiple Sclerosis: A Scoping Review
Source: Front Neurol. 2022 Jan 31;12:812147. doi: 10.3389/fneur.2021.812147 (PMC8841798; doi:10.3389/fneur.2021.812147)
Supplement: Appendix III — Characteristics of studies included in the scoping review. [file Table_3.docx]

| **Author**  **(Year)** | **Study region:**  ***Country/***  ***Countries*** | **Data Source(s)** | **Sample Size of Women with MS/CIS/RIS** | **Subtype(s) of Disease Included** | **Diagnostic criteria(s)**  **Accepted** | **Racial &**  **Ethnic Groups Included** | **Age of Participants (years)** | **Pregnancy Status of Participants**  **(n= number of pregnancies)** | **Level of Disability of Participants** | **Study**  **Topic [subtopic(s)]** | **Prospective and/or retrospective** | **Study**  **Type** | **Use of PROs** |
| --- | --- | --- | --- | --- | --- | --- | --- | --- | --- | --- | --- | --- | --- |
| *Achiron*[*^1^*](#_ENREF_1)  *(2020)* | Asia: *Israel* | Clinical registry | 2281 | RRMS (2281) | McDonald 2001, 2005, 2010 & Poser | Not reported | Median  Group 1: 29.9 (IQR 22.8-38.5)  Group 2: 29.4 (IQR 22.7-38.0) | Pregnant (n=1924) & Not pregnant | Median EDSS Group 1: 1.5 (IQR 1.0-2.5 )  Group 2: 1.5 (IQR 1.0-2.0) | Pregnancy  [Disability outcomes, DMT exposure] | Retrospective | Cohort | No |
| *Achiron*[*^2^*](#_ENREF_2)  *(1996)* | Not reported | Primary data collection | 9 | RRMS  (9) | Poser | Not reported | Mean 30 (SD ± 4.1, Range 22-35) | Pregnant (n=9) | Mean EDSS 3.4 (SD ± 3.4, Range 1.5-6.5) | Pregnancy  [MS relapses, DMT exposure] | Prospective | Case Series | No |
| *Achiron*[*^3^*](#_ENREF_3)  *(2004)* | Asia: *Israel* | Clinical registry & Medical records review | 108 | RRMS (108) | Poser | Not reported | Mean 28 (Range 26-38) | Pregnant (n=108) | Mean EDSS  Group 1: 1.8  Group 2: 1.6  Group 3: 1.7 | Pregnancy  [MS relapses, DMT exposure] | Retrospective | Cohort | No |
| *Jalkanen*[*^4^*](#_ENREF_4)  *(2010)* | Europe: *Finland* | Primary data collection &  Administr-ative data | 60 | RRMS (60) | McDonald 2001 & Poser | Not reported | Mean 30.5 (Range 23-42) | Pregnant (n=61) | Mean EDSS 1.31 | Pregnancy  [Pregnancy complications, MS relapses, Disability outcomes],  Fetal/  Neonatal Outcomes | Prospective | Cohort | No |
| *Airas*[*^5^*](#_ENREF_5)  *(2010)* | Europe: *Finland* | Primary data collection | 60 | MS subtype unspecified (60) | Not reported | Not reported | Mean 30.5 (Range 23-42) | Pregnant (n=61) & Not pregnant | Mean EDSS 1.33 (± SD 1.17) | Pregnancy, Breastfeedi-ng [MS relapses] | Prospective | Cohort | No |
| *Airas*[*^6^*](#_ENREF_6)  *(2007)* | Europe: *Finland* | Primary data collection | 60 | RRMS (60) | Not reported | White | Mean  Group 1: 29.5, Group 2: 35.7 | Pregnant (n=42) & Not pregnant | Mean EDSS  Group 1: 1.5  Group 2: 1.6 | Pregnancy  [Mechanism] | Prospective | Cohort | No |
| *Airas*[*^7^*](#_ENREF_7)  *(2007)* | Europe: *Finland* | Primary data collection | 33 | RRMS (33) | Not reported | Not reported | Mean 30.2 (±SD 3.8) | Pregnant (n=33) | Mean EDSS 1.5 (±SD 1.0) | Pregnancy  [MS relapses, Disability outcomes, Mechanism] | Prospective | Cohort | No |
| *Al-Shammri*[*^8^*](#_ENREF_8)  *(2004)* | Asia: *Kuwait* | Primary data collection | 8 | RRMS (8) | Poser | Not reported | Mean 30.3  (SD ±6.6, Range 21-37) | Pregnant (n=8) | EDSS Range 0-2 | Pregnancy  [Disability outcomes, DMT exposure, Mechanism] | Prospective | Case Series | Yes |
| *Alanazy*[*^9^*](#_ENREF_9)  *(2020)* | Asia: *Saudi Arabia* | Primary data collection | 120 | RRMS (53),  SPMS (11), PPMS/PRMS (6),  Subtype unknown (50) | Not reported | Not reported | Mean 29.9 | Pregnant (n=63) & Not pregnant | Not reported | Pregnancy, Services around family planning/  pregnancy/  post-pregnancy | Prospective | Cross-sectio-nal | Yes |
| *Alehashemi*[*^10^*](#_ENREF_10)  *(2019)* | Asia: *Iran* | Primary data collection | 64 | RRMS (60), SPMS (4) | McDonald 2010 | Not reported | Mean 35.25 | Not pregnant | EDSS Range  0-6 | Sexual dysfunction | Prospective | Case Control | Yes |
| *Alexander*[*^11^*](#_ENREF_11)  *(2018)* | North America: *United States* | Primary data collection | 20 | MS subtype unspecified (20) | Not reported | White &  African, Black, or African-American | Mean 46.7 | Not pregnant | Not reported | Sexual dysfunction | Prospective | Clinical trial | Yes |
| *Alping*[*^12^*](#_ENREF_12) *(2020)* | Europe: *Sweden* | Clinical registry & Administ-rative data | 7477 | RRMS (6302), SPMS (893), PPMS (179),  PRMS (100) | Not reported | Not reported | Mean  Group 1: 42.9  Group 2: 39.2  Group 3: 35.6 | Not pregnant | Mean EDSS 2.8 | Cancer/  Cancer screening | Retrospective | Cohort | No |
| *Alroughani*[*^13^*](#_ENREF_13)  *(2019)* | Asia: *Kuwait,*  *Lebanon* | Clinical registry | 132 | CIS (6),  RRMS (125), SPMS (1) | McDonald 2010 | Not reported | Mean 32.4 | Pregnant (n=164) | Mean EDSS 1.5 | Pregnancy  [MS relapses, DMT exposure] | Retrospective | Cohort | No |
| *Alroughani*[*^14^*](#_ENREF_14)  *(2018)* | Asia: *Kuwait* | Clinical registry | 87 | RRMS (87) | McDonald 2010 | Not reported | Mean 31.8 (± SD 5) | Pregnant (n=97) | Mean EDSS 1.6 (± SD 1.1) | Pregnancy | Prospective | Cohort | No |
| *Altintas*[*^15^*](#_ENREF_15)  *(2015)* | Asia: *Turkey* | Medical records review | 199 | RRMS (163), SPMS (18), PPMS (8),  PRMS (10) | McDonald 2001 | Not reported | Mean 45.03 (Range 23-73) | Pregnant  (n=558) | EDSS Range 0-8.5 | Pregnancy  [Pregnancy complicati-ons, MS relapses, Disability outcomes, DMT exposure], Fertility | Retrospective | Cohort | No |
| *Alwan*[*^16^*](#_ENREF_16)  *(2013)* | North America:  *United States, Canada* | Primary data collection | 4408 | MS subtype unspecified (4408) | Self-reported | White | Mean 49.6 (± SD 9.2) | Pregnant (n=1702) & Not pregnant | Not reported | Pregnancy  [Pregnancy complicati-ons, MS relapses, DMT exposure], Breastfeedi-ng  [MS relapses], Assisted Reproducti-on, Services around family planning/  pregnancy/  post-pregnancy | Retrospective | Cross-section-al | Yes |
| *Alwan*[*^17^*](#_ENREF_17)  *(2013)* | North America | Primary data collection | 590 | CIS, RRMS, SPMS, PPMS (not reported for full cohort) | Self-reported | Not reported | Mean  Group 1: 36.5 (± SD 6.5)  Group 2: 36.8 (± SD 6.2) | Pregnant (not reported) | EDSS Range 0-8.5 | Pregnancy, Services around family planning/  pregnancy/  post-pregnancy | Prospective | Cross-section-al | No |
| *Amato*[*^18^*](#_ENREF_18)  *(2010)* | Europe: *Italy* | Primary data collection | 388 | MS subtype unspecified (388) | McDonald 2001 | Not reported | Mean  Group 1: 32.6 Group 2: 31.7 | Pregnant  (n=396) | mean EDSS Group 1: 1.5  Group 2: 1.6 | Pregnancy  [Pregnancy complicati-ons, DMT exposure], Fetal/  Neonatal Outcomes | Prospective | Cohort | No |
| *Andersen*[*^19^*](#_ENREF_19)  *(2018)* | Europe: *Denmark* | Clinical registry & Primary data collection | 382 | MS subtype unspecified (382) | Time perspecti-ve diagnostic criteria of the Danish MS Registry | Not reported | Mean 29.7 (Range 16-46) | Pregnant  (n=424) | Not reported | Child health/  Developme-ntal outcomes | Retrospective | Cohort | Yes |
| *Andersen*[*^20^*](#_ENREF_20)  *(2018)* | Europe: *Denmark* | Clinical registry & Administrative data | 13 | MS subtype unspecified (13) | McDonald (year not specified) | Not reported | Mean 25.13 (Range 16-36) | Pregnant  (n=31) | Not reported | Pregnancy  [Pregnancy complicati-ons, DMT exposure],  Fetal/  Neonatal Outcomes | Prospective | Cohort | No |
| *Araz Altay*[*^21^*](#_ENREF_21) *(2020)* | Asia: *Turkey* | Primary data collection | 50 children/adolescents with parents with MS | RRMS, SPMS, PPMS  (not stratified by gender | McDonald 2017 | Not reported | Mean age of mothers 42.2 (± SD 6.1) | Not pregnant | Mean EDSS 2.9 | Child health/ developmental outcomes | Retrospective | Cross-section-al | No |
| *Azari-Barzandig*[*^22^*](#_ENREF_22)  *(2019)* | Asia: *Iran* | Primary data collection | 150 | RRMS (98), SPMS (10), PPMS (28),  PRMS (14) | Not reported | Not reported | Mean 36.64 (± SD 5.93, Range 18-45) | Not pregnant | Mean EDSS 7.39 (± SD 3.56) | Sexual dysfunction | Prospective | Cross-section-al | Yes |
| *Bader*[*^23^*](#_ENREF_23)  *(1988)* | North America: *United States* | Primary data collection & Medical records review | 20 | MS subtype unspecified (20) | Not reported | Not reported | Not reported | Pregnant  (n=32) | Not reported | Pregnancy  [MS relapses, Anesthesia] | Prospective & Retrospective | Cohort | No |
| *Bansil*[*^24^*](#_ENREF_24)  *(1999)* | North America: *United States* | Primary data collection | 30 | RRMS (20), MS other subtype (10) | Poser | Not reported | Mean  Group 1: 36.9 (± SD 5.4)  Group 2: 42.5 (± SD 9)  Group 3: 37.9 (± SD 7.4) | Not pregnant | Mean EDSS Group 1: 2.2 (± SD 1.7)  Group 2: 3.75 (± SD 1.8)  Group 3: 2.3 (± SD 1.7) | Sex hormones & MS | Prospective | Cohort | No |
| *Baroncini*[*^25^*](#_ENREF_25)  *(2019)* | Europe: *Italy* | Primary data collection | 148 | RRMS (141),  SPMS (7) | Not reported | Not reported | Mean 35.4 (Range 15-53) | Pregnant (n=109 parous women) & Not pregnant | EDSS Range 0-7.5 | Menopause  [MS relapses, Disability outcomes] | Retrospective | Cohort | No |
| *Bartnik*[*^26^*](#_ENREF_26)  *(2017)* | Europe: *Poland* | Primary data collection | 86 | RRMS (86) | Not reported | Not reported | Mean 32.03 (Range 20-51) | Not pregnant | EDSS Range 0-4 | Sexual dysfunction | Prospective | Cross-section-al | Yes |
| *Bartnik*[*^27^*](#_ENREF_27)  *(2018)* | Europe: *Poland* | Primary data collection | 218 | RRMS (133), SPMS (10), PPMS (7),  PRMS (27) | Self-reported | Not reported | Mean 34.6  (± SD 9.2,  Range 18-62) | Not pregnant | Mean EDSS 2.9 (±SD 2.0, Range 0-7.5) | Birth Control, Cancer/  Cancer screening, Services around family planning/  pregnancy/  post-pregnancy | Retrospective | Cross-section-al | No |
| *Becker*[*^28^*](#_ENREF_28)  *(1997)* | Not reported | Primary data collection | 2 | MS subtype unspecified (2) | Not reported | White | Not reported | Pregnant (not reported) | Not reported | Birth Control, Services around family planning/  pregnancy/  post-pregnancy | Prospective | Cross-section-al | No |
| *Benoit*[*^29^*](#_ENREF_29)  *(2016)* | Europe: *France, Italy* | Clinical registry | 93 | CIS (12), RRMS (81) | McDonald 2005 & Poser | Not reported | Mean 32.1  (± SD 4.1) | Pregnant (n= at least 186) & Not pregnant | Mean DSS 1.9 (±SD 1.5) | Pregnancy  [MS relapses, Disability outcomes, Anesthesia],Breastfeedi-ng [MS relapses, Disability outcomes] | Prospective & Retrospective | Cohort | No |
| *Berenguer-Ruiz*[*^30^*](#_ENREF_30)  *(2019)* | Europe: *Spain* | Primary data collection | 66 | RRMS (66) | McDonald 2010 | Not reported | Mean 32.1 | Pregnant  (n=65) | Mean EDSS 1.2 | Pregnancy  [Pregnancy complications, MS relapses, Disability outcomes, DMT exposure],  Fetal/  Neonatal Outcomes | Prospective | Cohort | No |
| *Bernardi*[*^31^*](#_ENREF_31)  *(1991)* | Europe: *Italy* | Primary data collection | 52 | RRMS (52) | Schumac-her &  McAlpine | Not reported | Mean  Group 1: 36.1  Group 2: 38.9 | Pregnant (n=66) | Not reported | Pregnancy  [MS relapses] | Retrospective | Case Control | No |
| *Birk*[*^32^*](#_ENREF_32)  *(1990)* | North America: *United States* | Primary data collection | 8 | RRMS (6), SPMS (2), PPMS (0) | Schumac-her | White & Not reported | Mean 31.25 (Range 25-39) | Pregnant (n=8) | EDSS Range 1- 6 | Pregnancy  [Pregnancy complicati-ons, MS relapses, Disability outcomes, Mechanisms] | Prospective | Cohort | No |
| *Blackmore*[*^33^*](#_ENREF_33)  *(2011)* | North America: *United States* | Primary data collection | 62 | MS subtype unspecified (62) | Diagnosis confirmed by treating neurologi-st | White, African, Black, or African-American, Asian, Hispanic, Latino, Central or South American, Native American | Mean 46.9  (± SD 9.72) | Not pregnant | Not reported | Sexual dysfunction | Prospective | Cohort | Yes |
| *Borello-France*[*^34^*](#_ENREF_34)  *(2004)* | North America | Primary data collection | 122 | Not reported (122) | Not reported | White, African, Black, or African-American, Other | Mean 48.31  (± SD 10.47) | Not pregnant | Not reported | Sexual dysfunction | Prospective | Case Series | Yes |
| *Boskovic*[*^35^*](#_ENREF_35)  *(2005)* | North America: *Canada* | Primary data collection | 28 | MS subtype unspecified (28) | Self-reported | Not reported | Mean Group 1: 35.6 (± SD 4.5)  Group 2: 30.2 (± SD 4.8) | Pregnant (n=23) | Not reported | Pregnancy  [Pregnancy complicati-ons, DMT exposure] | Prospective & Retrospective | Cohort | No |
| *Bove*[*^36^*](#_ENREF_36)  *(2015)* | North America: *United States* | Primary data collection | 432 | RRMS (251), SPMS (85), PPMS (24), PRMS (6), Not reported (34) | Self-reported | White & Hispanic, Latino, Central or South American | Mean  Group 1: 40.3  Group 2: 57.4  Group 3: 51.7 | Not pregnant | MS rating score  Group 1: 8.9  (±SD 5.5)  Group 2: 9.6  (±SD 5.1)  Group 3: 13.1  (±SD 5.4) | Menopause  [Disability outcomes] | Prospective | Cohort | Yes |
| *Bove*[*^37^*](#_ENREF_37)  *(2018)* | North America: *United States* | Primary data collection | 162 | RIS (1), CIS (50), RRMS (111) | McDonald 2005 | Not reported | Mean  Group 1: 31.4 (± SD 7.2)  Group 2: 40.3 (±SD 7.8)  Group 3: 37.9 (± SD 10.6) | Not pregnant | Median EDSS 1.0 | Birth Control | Prospective | Cohort | No |
| *Bove*[*^38^*](#_ENREF_38)  *(2020)* | North America: *United States,* South America: *Argentina,* Europe: *France, Germany* | Primary data collection | 12 | CIS (2), RRMS (10) | Not reported | Not reported | Mean 35.1  (± SD 3.6, Range 26-41) | Pregnant (n=9 ART successes) | Not reported | Assisted Reproducti-on | Prospective | Case Series | No |
| *Bove*[*^39^*](#_ENREF_39)  *(2016)* | Not reported | Primary data collection | 127 | RRMS (72), SPMS (36), PPMS (15), PRMS (5) | Not reported | White & Hispanic, Latino, Central or South American | Mean 54.3 | Not reported | Not reported | Menopause  [Disability outcomes, Hormone replacement therapy, Symptoms] | Retrospective | Cross-section-al | Yes |
| *Bove*[*^40^*](#_ENREF_40)  *(2016)* | North America: *United States* | Primary data collection | 724 | CIS (320, RRMS (527), SPMS (103), PPMS (21), PRMS (7) | McDonald 2005 | White, Hispanic, Latino, Central or South American, Other | Mean  Group 1: 40  (± SD 7.6)  Group 2: 58.4  (±SD 7.1)  Group 3: 56.9  (± SD 8.6)  Group 4: 53.4  (± SD 8.6) | Not pregnant | Mean EDSS  Group 1: 1.56  (± SD 1.57)  Group 2: 2.77  (± SD 2.44) Group 3: 3.01  (± SD 2.3) Group 4: 4.88  (± SD 2.13) | Menopause  [Disability outcomes, Hormone replacement therapy] | Retrospective | Cross-section-al | Yes |
| *Bove*[*^41^*](#_ENREF_41)  *(2016)* | North America: *United States* | Primary data collection | 95 | Not reported (95) | Not reported | White & Other | Mean  Group 1: 56.8  Group 2: 56.4 | Not pregnant | Not reported | Menopause  [Hormone replacement therapy] | Prospective | Cohort | Yes |
| *Boz*[*^42^*](#_ENREF_42)  *(2018)* | Asia: *Turkey* | Primary data collection | 16 | MS subtype unspecified (16) | Physician reported | Not reported | Mean 29.82  (± SD 4.6) | Not pregnant | Mean EDSS 1.5  (± SD 0.7) | Breastfeedi-ng  [non-DMT drug exposure],  Fetal/  Neonatal Outcomes,  Child health/ developme-ntal outcomes | Prospective | Cohort | No |
| *Brandt-Wouters*[*^43^*](#_ENREF_43)  *(2016)* | Europe: *Netherla-nds* | Medical records review | 31 | RRMS (31) | McDonald 2010 | Not reported | Mean 31 | Pregnant  (n=42) | Not reported | Pregnancy  [MS relapses, DMT exposure] | Retrospective | Cohort | No |
| *Bsteh*[*^44^*](#_ENREF_44)  *(2018)* | Europe: *Austria* | Clinical registry | 239 | RRMS (239) | McDonald 2005 & 2010 | Not reported | Mean 30.7 | Pregnant  (n=387) | EDSS Range 0-5 | Pregnancy  [Pregnancy complicati-ons, MS relapses, Disability outcomes, DMT exposure],  Fetal/  Neonatal Outcomes | Retrospective | Cohort | No |
| *Burkill*[*^45^*](#_ENREF_45)  *(2019)* | Europe: *Sweden, Finland* | Administ-rative data | 1131 | Not reported (1131) | ICD-10 code & Not reported | Not reported | Mean  Group 1: 31.3  Group 2: 32.3  Group 3: 30.0  Group 4: 30.6 | Pregnant  (n=1809) | Not reported | Pregnancy  [DMT exposure],  Fetal/  Neonatal Outcomes | Retrospective | Cohort | No |
| *Cardenas-Robledo*[*^46^*](#_ENREF_46)  *(2019)* | South America: *Colombia* | Primary data collection | 140 | CIS (9),  RRMS (121), SPMS (7), PPMS (3) | McDonald 2010 | Not reported | Mean 41 (Range 18-68) | Not pregnant | Not reported | Birth Control | Retrospective | Cross-section-al | No |
| *Contentti*[*^47^*](#_ENREF_47)  *(2019)* | South America: *Argentina* | Primary data collection | 137 | RRMS (112), SPMS (18), PPMS (9) | McDonald 2010 | Not reported | Mean 38.8  (± SD 9.2) | Not pregnant | Self-reported disability according to Kobelt et al, mean 2.4  (± SD 1.9) | Sexual dysfunction | Retrospective | Cross-section-al | Yes |
| *Carvalho*[*^48^*](#_ENREF_48)  *(2014)* | Europe: *Portugal* | Primary data collection & Medical records review | 100 | RRMS (90), SPMS (8), PPMS (2) | McDonald 2005 | Not reported | Mean 40  (Range 35-45) | Pregnant (n=43) &  Not pregnant | Not reported | Pregnancy  [MS relapses],  Motherhood/parenthood | Retrospective | Cohort | Yes |
| *Celik*[*^49^*](#_ENREF_49)  *(2013)* | Asia: *Turkey* | Primary data collection | 44 | CIS*,* RRMS,  SPMS,  PPMS  (not stratified by gender) | McDonald 2005 | Not reported | Mean 37.4 (Range 21-56)  *Combined for all genders or sexes | Not pregnant | EDSS Range 0-7.5 | Sexual dysfunction | Prospective | Cross-section-al | Yes |
| *Chakravarty*[*^50^*](#_ENREF_50)  *(2011)* | North America: *United States,* Europe: *United Kingdom,*  *Sweden, France, Switzerla-nd, Netherla-nds, Germany, Norway*,  Australia/New Zealand: *Australia* | Clinical registry | 2 | MS subtype unspecified (2) | Not reported | Not reported | Not reported | Pregnant (n=3) | Not reported | Pregnancy  [Pregnancy complicati-ons],  Fetal/  Neonatal Outcomes | Retrospective | Cohort | No |
| *Chen*[*^51^*](#_ENREF_51)  *(2020)* | North America: *United States* | Medical records review | 46 | CIS (2), RRMS (44) | Not reported | Not reported | Mean 35.6 | Not pregnant | Median EDSS 1.5 | Sex hormones & MS | Retrospective | Cohort | No |
| *Chen*[*^52^*](#_ENREF_52)  *(2009)* | Asia: *Taiwan* | Administ-rative data | 174 | MS subtype unspecified (174) | ICD-9 code | Not reported | Not reported | Pregnant (n=174) | Not reported | Pregnancy  [Pregnancy complicati-ons] | Retrospective | Cohort | No |
| *Christopherson*[*^53^*](#_ENREF_53)  *(2006)* | North America: *Canada* | Primary data collection | 62 | RRMS (31), SPMS (9), PPMS (16), MS subtype other (6) | Registered at an MS clinic | Not reported | Mean 44 (Range 24-57) | Not pregnant | Mean EDSS Group 1: 3.5  (± SD 0.5)  Group 2:  3.7  (± SD 0.5) | Sexual dysfunction | Prospective | Clinical trial | Yes |
| *Cil*[*^54^*](#_ENREF_54)  *(2009)* | Asia: *Turkey* | Primary data collection | 22 | RRMS (20), SPMS (1), PPMS (1) | Not reported | Not reported | Mean 33.21  (Range 23-41) | Pregnant  (n=57) | Not reported | Fertility, Sex hormones & MS | Prospective | Case Control | No |
| *Ciplea*[*^55^*](#_ENREF_55)  *(2020)* | Europe: *Germany* | Clinical registry & Primary data collection | 21 | MS subtype unspecified (21) | Not reported | Not reported | Mean 34.2  *Included MS and NMOSD patients | Pregnant (n=21) | EDSS Range 0-6 | Pregnancy  [Pregnancy complicati-ons, DMT exposure], Breastfeedi-ng [DMT exposure], Fetal/  Neonatal Outcomes | Prospective | Cohort | No |
| *Ciplea*[*^56^*](#_ENREF_56)  *(2020)* | Europe: *Germany* | Clinical registry | 69 | MS subtype unspecified (69) | Not reported | Not reported | Mean 33.5 | Pregnant  (n=73) | Not reported | Pregnancy  [DMT exposure], Breastfeedi-ng, Fetal/  Neonatal Outcomes | Prospective | Cohort | No |
| *Cocco*[*^57^*](#_ENREF_57)  *(2008)* | Europe: *Italy* | Primary data collection | 189 | RRMS (107),  SPMS (77), PPMS (4) | McDonald 2001 & 2005 | Not reported | Mean 35 (Range 14-45) | Pregnant (n=10) & Not pregnant | Not reported | Fertility | Retrospective | Cohort | No |
| *Confavreux*[*^58^*](#_ENREF_58)  *(1998)* | Europe: *France* | Primary data collection | 254 | RRMS (246), SPMS (8) | Poser | Not reported | Mean  Group 1: 30  (± SD 4)  Group 2: 30  (± SD 3)  Group 3: 32  (± SD 4) | Pregnant (n=269) | Mean EDSS Group 1: 1.3 (± SD 1.4)  Group 2: 1 (± SD 1.1)  Group 3: 2.1 (± SD 1.6) | Pregnancy  [MS relapses, Disability outcomes, Anesthesia], Breastfeedi-ng [MS relapses, Disability outcomes] | Prospective | Cohort | No |
| *Correale*[*^59^*](#_ENREF_59)  *(2012)* | South America: *Argentina* | Primary data collection | 16 | RRMS (16) | McDonald 2001 | Not reported | Not reported | Pregnant  (n=7) & Not pregnant | Not reported | Assisted Reproducti-on | Prospective | Case Series | No |
| *Coyle*[*^60^*](#_ENREF_60)  *(2014)* | North America: *United States* | Clinical registry | 96 | MS subtype unspecified (96) | Not reported | White, African, Black, or African-American,Hispanic, Latino, Central or South American, Other, Not reported | Mean 30.9 (Range 19-44) | Pregnant  (n=96) | Not reported | Pregnancy  [Pregnancy complicati-ons, DMT exposure], Fetal/  Neonatal Outcomes | Prospective | Cohort | No |
| *Cuello*[*^61^*](#_ENREF_61)  *(2017)* | Europe: *Spain* | Primary data collection | 54 | RRMS (48), PPMS (3),  PRMS (3) | McDonald 2005 & 2010 | Not reported | Mean 32.7 | Pregnant (n=54) | EDSS Range 0-6 | Fertility | Retrospective | Case Control | No |
| *Cuello*[*^62^*](#_ENREF_62)  *(2019)* | Europe: *Spain* | Primary data collection | 39 | RRMS (39) | McDonald 2005 & 2010 | Not reported | Median 33 (IQR 31-36) | Pregnant  (n=39) | Median EDSS 1  (IQR 0-1) | Pregnancy  [Mechanism] | Prospective | Cohort | No |
| *Cuello*[*^63^*](#_ENREF_63)  *(2017)* | Europe: *Spain* | Primary data collection | 35 | RRMS (32), SPMS (2), PPMS (1) | McDonald 2010 | Not reported | Mean 32.3 (Range 20-40) | Pregnant  (n=40) | EDSS Range 0-5 | Pregnancy  [Pregnancy complicati-ons, MS relapses, Disability outcomes] | Prospective | Cohort | No |
| *Cuello*[*^64^*](#_ENREF_64)  *(2020)* | Europe: *Spain* | Primary data collection | 53 | RRMS (53) | McDonald  2005 & 2010 | Not reported | Median  Group 1: 32  (IQR 30-34)  Group 2: 35  (IQR 32-37) | Pregnant (n=53) | Median EDSS  Group 1: 0.5 (IQR 0-1.5); Group 2: 1 (IQR 0-1) | Pregnancy  [MS relapses, Anesthesia] Sex hormones & MS  [Mechanism] | Prospective | Cohort | No |
| *Cuello*[*^65^*](#_ENREF_65)  *(2020)* | Europe: *Spain* | Primary data collection | 58 | RRMS (54), SPMS (1), PPMS (3) | McDonald 2017 | Not reported | Mean  Group 1: 32  Group 2: 33  (Range overall 20-41) | Pregnant  (n=68) | Mean EDSS 1  (Range 0-6) | Pregnancy  [Pregnancy complicati-ons, MS relapses, DMT] | Prospective | Case Control | No |
| *D'Hooghe*[*^66^*](#_ENREF_66)  *(2012)* | Europe: *Belgium* | Primary data collection | 973 | Relapsing onset MS (675), PPMS (298) | Diagnosed by a neurologi-st | Not reported | Mean  Group 1: 49.6  Group 2: 58.2 | Not pregnant | Percentage of group with EDSS ≥ 6.0,  Group 1: 34%  Group 2: 78.8% | Birth Control, Pregnancy  [Disability outcomes], Menarche/  puberty | Retrospective | Cross-section-al | No |
| *D'Hooghe*[*^67^*](#_ENREF_67)  *(2010)* | Europe: *Belgium, Netherla-nds* | Medical records review | 330 | MS subtype unspecified (330) | Poser | Not reported | Mean age Group 1: 28.2  Group 2: 37.8  Group 3: 22.2 Group 4: 28.2 Range overall (16-43) | Pregnant (250 parous women) & Not pregnant | Mean EDSS 5.6 | Pregnancy  [Disability outcomes] | Retrospective | Cross-section-al | No |
| *Dahl*[*^68^*](#_ENREF_68)  *(2008)* | Europe: *Norway* | Clinical registry & Administrative data | 1368 | RRMS, MS other subtype  (number not reported by participant) | McDonald 2001 | Not reported | Mean 33.3 | Pregnant  (n=2773) | Not reported | Pregnancy  [Pregnancy complicati-ons], Fetal/  Neonatal Outcomes | Retrospective | Cohort | No |
| *Dahl*[*^69^*](#_ENREF_69)  *(2006)* | Europe: *Norway* | Clinical registry | Not reported | MS subtype unspecified | Not reported | Not reported | Mean 30.2 | Pregnant  (n=449) | Not reported | Pregnancy  [Pregnancy complicati-ons], Fetal/  Neonatal Outcomes | Retrospective | Cohort | No |
| *Dahl*[*^70^*](#_ENREF_70)  *(2005)* | Europe: *Norway* | Administ-rative data | 461 | MS subtype unspecified (461) | Not reported | Not reported | Mean 30  (±SD 4.6) | Pregnant (n=649) | Not reported | Pregnancy  [Pregnancy complicati-ons], Fetal/  Neonatal Outcomes | Retrospective | Case Control |  |
| *Dahl*[*^71^*](#_ENREF_71)  *(2007)* | Europe: *Norway* | Administrative data | Not reported | MS subtype unspecified | Not reported | Not reported | Not reported | Pregnant (n=250 births) | Not reported | Pregnancy  [Pregnancy complicati-ons], Fetal/  Neonatal Outcomes | Retrospective | Cross-section-al | No |
| *Darija*[*^72^*](#_ENREF_72)  *(2015)* | Europe: *Serbia* | Primary data collection | 66 | RRMS, SPMS, PPMS  Not stratified by gender | McDonald 2001 | Not reported | Mean age 41.5 (± SD 8.5, Range 20-61)  *Combined for all genders or sexes | Not pregnant | Mean EDSS 4.2 (± SD 1.6, Range 1-7.5)  *Combined for all genders or sexes | Sexual dysfunction | Prospective | Cohort | Yes |
| *de Andres*[*^73^*](#_ENREF_73)  *(2017)* | Europe: *Spain* | Primary data collection | 60 | MS subtype unspecified (60) | McDonald 2010 | Not reported | Mean  Group 1: 34  (Range 31-36)  Group 2: 39  (Range 28-51) | Pregnant (n=30) & Not pregnant | Median EDSS  Group 1: 0  (IQR 0-1)  Group 2: 1  (IQR 0-1.6) | Pregnancy  [Mechanism] | Prospective | Case Control | No |
| *De Giglio*[*^74^*](#_ENREF_74)  *(2015)* | Europe: *Italy* | Not reported | 4 | RRMS (4) | Not reported | Not reported | Age range  33-38 | Pregnant (n=4) | EDSS Range 2-8 | Pregnancy  [MS relapses, DMT exposure] | Retrospective | Case Series | No |
| *De Giglio*[*^75^*](#_ENREF_75)  *(2015)* | Europe: *Italy* | Primary data collection | 106 | RRMS (106) | McDonald 2001 | Not reported | Mean 30  (± SD 6) | Not pregnant | Median EDSS 1.5  (Range 0-4) | Sex hormones & MS | Prospective | Cross-section-al |  |
| *De Giglio*[*^76^*](#_ENREF_76)  *(2017)* | Europe: *Italy* | Primary data collection | 142 | RRMS (142) | McDonald 2001 | Not reported | Mean  Group 1: 30.4  (± SD 7)  Group 2: 29.1 (± SD 6.4)  Group 3: 30.6  (± SD 5.9) | Not pregnant | Median EDSS  Group 1: 1.7 (±SD 0.7) Group 2: 1.8 (± SD 0.9) Group 3: 1.6 (± SD 1.0) | Sex hormones & MS | Prospective | Clinical trial | No |
| *De las Heras*[*^77^*](#_ENREF_77)  *(2007)* | Europe: *Spain* | Medical records review | 74 | RRMS (68), SPMS (6) | Poser | Not reported | Mean 30.5  (± SD 4.2) | Pregnant (n=88) | Median EDSS  Group 1: 1.5 Group 2: 4.5 | Pregnancy  [Pregnancy complicati-ons, DMT exposure],  Fetal/  Neonatal Outcomes | Retrospective | Cohort | No |
| *de Seze*[*^78^*](#_ENREF_78)  *(2004)* | Europe: *France* | Primary data collection | 42 | MS subtype unspecified (42) | Not reported | Not reported | Mean  Group 1: 28.6  Group 2: 27.9 | Pregnant (n=42) | Mean EDSS  Group 1: 2.7  Group 2: 2.5 | Pregnancy  [MS relapses, DMT exposure] | Prospective | Cohort | No |
| *Deatrick*[*^79^*](#_ENREF_79)  *(1998)* | North America: *United States* | Primary data collection | 35 | RRMS (35) | Not reported | White & African, Black, or African-American | Mean 37 (Range 29-46) | Pregnant (n=35) | EDSS Range 1-6.5 | Motherhood/parenthood | Prospective | Cross-section-al | Yes |
| *Dehghan-Nayeri*[*^80^*](#_ENREF_80)  *(2017)* | Asia:  *Iran* | Primary data collection | 207 | RRMS (128), SPMS (41), PPMS (13) | Definite MS diagnosis by a neurologi-st | Not reported | Mean 36.9  (± SD 8.1, Range 22-58) | Not pregnant | EDSS split into categories with some patients in all groups | Sexual dysfunction | Retrospective | Cross-section-al | Yes |
| *Demirkiran*[*^81^*](#_ENREF_81)  *(2006)* | Asia: *Turkey* | Primary data collection | 51 | RRMS (35), SPMS (9), PPMS (7) | Poser | Not reported | Mean  Group 1: 37.9  Group 2: 31.6  (Overall range 20-53) | Not pregnant | EDSS Range 0-7.5 | Sexual dysfunction | Retrospective | Cross-section-al | Yes |
| *Demortiere*[*^82^*](#_ENREF_82)  *(2020)* | Europe: *France* | Primary data collection | 39 | RRMS (39) | Not reported | Not reported | Age Range 28.6-35.2 | Pregnant  (n=46) | EDSS Range 1-3 | Pregnancy  [Pregnancy complications, MS relapses, Disability outcomes, DMT exposure], Fetal/  Neonatal Outcomes | Prospective | Cohort | No |
| *Diareme*[*^83^*](#_ENREF_83)  *(2006)* | Europe: *Greece* | Primary data collection | 40 | MS subtype unspecified (40) | Not reported | Not reported | Mean 40.02  (± SD 4.07) | Not pregnant | Not reported | Child health/developmental outcomes, Motherhood/parenthood | Prospective | Case Control | Yes |
| *Dobos*[*^84^*](#_ENREF_84)  *(2015)* | North America: *United States* | Clinical registry | 67 | CIS (3), RRMS (2), SPMS (54), PPMS (8) | Not reported | Not reported | Mean 56.8 | Not pregnant | EDSS Range 7-9.5 | Cancer/  Cancer screening | Retrospective | Cohort | No |
| *Doosti*[*^85^*](#_ENREF_85)  *(2018)* | Asia: *Iran* | Primary data collection | 86 | RRMS (43), SPMS (43) | McDonald 2010 | Not reported | Mean  Group 1: 38.84  Group 2: 39.21 | Not pregnant | Mean EDSS: Group 1: 1.13  Group 2: 3.89 | Cancer/  Cancer screening | Prospective | Cohort | No |
| *Dugue*[*^86^*](#_ENREF_86)  *(2014)* | Europe: *Denmark* | Administ-rative data | 14,403 | MS subtype unspecified (14403) | ICD-9 or 10 Code | Not reported | Not reported | Not pregnant | Not reported | Cancer/  Cancer screening | Retrospective | Cohort | No |
| *Durufle*[*^87^*](#_ENREF_87)  *(2006)* | Europe: *France* | Clinical registry & Medical records review | 368 | RRMS (175),  SPMS (126), PPMS (67) | Poser | Not reported | Mean 42.9 | Pregnant (273 parous women) & Not pregnant | Mean EDSS 4.4 | Pregnancy  [Pregnancy complicati-ons] | Retrospective | Cohort | No |
| *Durufle*[*^88^*](#_ENREF_88)  *(2006)* | Europe: *France* | Medical records review | 102 | RRMS (28), MS other subtype (74) | Poser | Not reported | Mean 44.7 | Pregnant (n=71 parous women) & Not pregnant | Mean EDSS 5.4 | Pregnancy  [Pregnancy complicati-ons] | Retrospective | Cohort | Yes |
| *Ebrahimi*[*^89^*](#_ENREF_89)  *(2015)* | Europe: *Germany,* North America: *Canada* | Clinical registry & Primary data collection | 179 | RRMS (179) | Not reported | Not reported | Mean  Group 1: 30.5  Group 2: 33.9 | Pregnant (n=102) | Not reported | Pregnancy  [Pregnancy complicati-ons, MS relapses, DMT exposure], Fetal/  Neonatal Outcomes | Prospective | Case Control | No |
| *Eftekhari*[*^90^*](#_ENREF_90)  *(2014)* | Asia: *Iran* | Primary data collection | 24 | RRMS (24) | Not reported | Not reported | Mean  Group 1: 35.08  Group 2: 33.75 | Not pregnant | Mean EDSS  Group 1: 2.87  Group 2: 2.79 | Sex hormones & MS | Prospective | Clinical trial | No |
| *Egerod*[*^91^*](#_ENREF_91)  *(2018)* | Europe: *Denmark* | Primary data collection | 5 | MS subtype unspecified (5) | Not reported | Not reported | Mean 49  (Range 33-57) | Not pregnant | Not reported | Sexual dysfunction | Prospective | Cross-section-al | Yes |
| *Ehrlich*[*^92^*](#_ENREF_92)  *(2007)* | Not reported | Primary data collection | 61 | RRMS (61) | Poser | Not reported | Not reported | Pregnant (n=61) | EDSS Range 0-7.5 | Pregnancy  [Mechanism] | Prospective | Cohort | No |
| *Etemadifar*[*^93^*](#_ENREF_93)  *(2015)* | Asia: *Iran* | Primary data collection | 15 | MS subtype unspecified (15) | McDonald 2010 | Not reported | Mean  Group 1: 27.7  (± SD 2.4)  Group 2: 30.0  (± SD 3.9) | Pregnant (n=15) | Mean EDSS Group 1: 1.2 (± SD 0.3) Group 2: 1.3 (± SD 0.4*)* | Pregnancy  [MS relapses , Disability outcomes] | Prospective | Clinical trial | No |
| *Falaschi*[*^94^*](#_ENREF_94)  *(2001)* | Europe: *Italy* | Primary data collection | 76 | MS subtype unspecified (76) | Poser | Not reported | Mean 34.9  (± SD 0.9) | Not pregnant | Not reported | Birth Control, Menarche/  Puberty, Menstruati-on | Retrospective | Cross-section-al | No |
| *Fares*[*^95^*](#_ENREF_95)  *(2016)* | Asia | Primary data collection | 29 | RRMS (29) | McDonald 2010 | Not reported | Mean 23 (Range 16-34) | Pregnant (n=64) | Not reported | Pregnancy  [Pregnancy complicati-ons, MS relapses,  DMT exposure, Anesthesia], Breastfeedi-ng [MS relapses],  Fetal/  Neonatal Outcomes | Prospective | Cohort | No |
| *Fernandez*[*^96^*](#_ENREF_96)  *(2009)* | South America: *Argentina* | Primary data collection | 81 | RRMS (72), SPMS (7), PPMS (1) | McDonald 2001 | Not reported | Mean 39 (Range 22-72) | Pregnant (n=141) | Not reported | Pregnancy  [Pregnancy complicati-ons, MS relapses, DMT exposure], Breastfeedi-ng [MS relapses], Fetal/  Neonatal Outcomes | Retrospective | Cross-section-al | No |
| *Ferraro*[*^97^*](#_ENREF_97)  *(2017)* | Europe: *Italy* | Primary data collection | 303 | Not reported (303) | Not reported | Not reported | Mean 52 | Not pregnant | Not reported | Pregnancy  [Pregnancy complicati-ons], Fertility, Assisted Reproducti-on | Prospective | Case Control | No |
| *Finkelsztejn*[*^98^*](#_ENREF_98)  *(2011)* | South America: *Brazil* | Clinical registry & Medical records review | 128 | MS subtype unspecified (128) | McDonald 2001 | Not reported | Mean 29.8 (Range 16-42) | Pregnant  (n= 142) | Mean EDSS 1.5 | Pregnancy  [Pregnancy complicati-ons, MS relapses, DMT exposure], Breastfeedi-ng, Fetal/  Neonatal Outcomes | Retrospective | Cohort | No |
| *Firdolas*[*^99^*](#_ENREF_99)  *(2013)* | Asia: *Turkey* | Primary data collection | 23 | RRMS (17), SPMS (6) | Poser | Not reported | Mean  Group 1: 31.9  Group 2: 34.3 | Not pregnant | Mean EDSS 3.02 | Sexual dysfunction | Prospective | Cross-section-al | Yes |
| *Fong*[*^100^*](#_ENREF_100)  *(2018)* | North America: *United States* | Administ-rative data | 1185 | MS subtype unspecified (1185) | ICD-9 code | White, African, Black, or African-American, Asian, Hispanic, Latino, Central or South American, Other | Not reported | Pregnant (n=1185) | Not reported | Pregnancy  [Pregnancy complicati-ons] | Retrospective | Cohort | No |
| *Foroughipour*[*^101^*](#_ENREF_101)  *(2012)* | Asia: *Iran* | Primary data collection | 16 | RRMS (16) | Poser | Not reported | Mean 27.2 | Not pregnant | EDSS Range 1-5.5 | Sex hormones & MS | Retrospective | Cohort | No |
| *Fragala*[*^102^*](#_ENREF_102)  *(2014)* | Europe: *Italy* | Primary data collection | 75 | RRMS, SPMS, PPMS  (not stratified by gender) | McDonald revised criteria (year not stated) | Not reported | Median 46 (IQR 41.5-53.5) *Combined for all genders or sexes | Not pregnant | Median EDSS 4.5 (IQR 2.9-6.0)  *Combined for all genders or sexes | Sexual dysfunction | Prospective | Cross-section-al | Yes |
| *Fragoso*[*^103^*](#_ENREF_103)  *(2013)* | South America: *Brazil* | Clinical registry | 180 | RRMS (167), SPMS (10), PPMS (3) | Not reported | Not reported | Mean 30.1 (Range 18- 45) | Pregnant  (not reported) | Not reported | Pregnancy  [Pregnancy complicati-ons, DMT exposure], Fetal/  Neonatal Outcomes, Child health/  Developme-ntal outcomes | Retrospective | Cohort | No |
| *Fragoso*[*^104^*](#_ENREF_104)  *(2010)* | South America | Primary data collection | 11 | RRMS (11) | McDonald 2001 | Hispanic, Latino, Central or South American | Age Range 17-35 | Pregnant (n=11) | EDSS Range 0- 4.5 | Pregnancy  [Pregnancy complicati-ons, MS relapses,  Disability outcomes, DMT exposure], Fetal/  Neonatal Outcomes | Retrospective | Case Series | No |
| *Fragoso*[*^105^*](#_ENREF_105)  *(2015)* | South America: *Brazil* | Medical records review | 134 | RRMS (134) | Not reported | Not reported | Mean  Group 1: 31.0  Group 2: 29.4 | Pregnant  (n=134) | Not reported | Pregnancy  [MS relapses, DMT exposure] | Retrospective | Case Control | No |
| *Fragoso*[*^106^*](#_ENREF_106)  *(2013)* | North America: *Mexico*, South America: *Brazil, Argentina,* Europe: *United Kingdom* | Medical records review | 132 | RRMS, SPMS, PPMS  (not reported per 132 patients) | McDonald 2010 | Not reported | Mean  Group 1: 34.8  Group 2: 34.7  Group 3: 32.0 | Pregnant  (n=152) | EDSS Range 0-5 | Pregnancy  [Pregnancy complicati-ons, Disability outcomes, DMT exposure], Fetal/  Neonatal Outcomes | Retrospective | Cohort | No |
| *Fragoso*[*^107^*](#_ENREF_107)  *(2009)* | South America: *Brazil* | Clinical registry | 47 | MS subtype unspecified (47) | Not reported | Not reported | Mean 28.4 | Pregnant  (n=49) | Mean EDSS 1.36 | Pregnancy  [Pregnancy complicati-ons, MS relapses, DMT exposure], Fetal/  Neonatal Outcomes | Retrospective | Cohort | No |
| *Frau*[*^108^*](#_ENREF_108)  *(2018)* | Europe: *Italy* | Primary data collection | 158 | RRMS (158) | McDonald 2001 & 2005,  Poser | Not reported | Mean 31  *Combined for all genders or sexes | Pregnant (n=137) | Mean EDSS 4 (± SD 3.5)  *Combined for all genders or sexes | Pregnancy  [Pregnancy complicati-ons, DMT exposure], Fetal/  Neonatal Outcomes | Prospective | Cohort | No |
| *Friend*[*^109^*](#_ENREF_109)  *(2016)* | North America, Europe | Primary data collection | 362 | MS subtype unspecified (362) | Not reported | White, African, Black, or African-American, Asian, Hispanic, Latino, Central or South American, Other | Age Range ≤20-50 | Pregnant  (n=363) | Not reported | Pregnancy  [DMT exposure], Fetal/  Neonatal Outcomes | Prospective | Case Series | No |
| *Frith*[*^110^*](#_ENREF_110)  *(1988)* | Australia/New Zealand: *Australia* | Primary data collection & Medical records review | 52 | MS subtype unspecified (52) | Schumac-her | Not reported | Not reported | Pregnant  (n=101) | Not reported | Pregnancy  [MS relapses] | Retrospective | Cohort | No |
| *Frith*[*^111^*](#_ENREF_111)  *(1987)* | Australia/New Zealand | Primary data collection | 407 | MS subtype unspecified (407) | Schumac-her | Not reported | Not reported | Pregnant  (n=52) | Not reported | Pregnancy  [MS relapses] | Prospective | Case Control | No |
| *Gagliardi*[*^112^*](#_ENREF_112)  *(2003)* | North America: *United States* | Primary data collection | 5 | Not reported (5) | Not reported | Not reported | Age Range  47-71  *Combined for all genders or sexes | Not pregnant | Not reported | Sexual dysfunction | Prospective | Cross-section-al | Yes |
| *Gava*[*^113^*](#_ENREF_113)  *(2014)* | Europe: *Italy* | Medical records review & Primary data collection | 183 | RRMS (183) | McDonald 2010 | Not reported | Mean  Group 1: 40.8  Group 2: 47.5 | Not pregnant | Mean EDSS  Group 1: 2.4  Group 2: 3.4 | Birth Control, Sex hormones & MS | Retrospective | Cross-section-al | No |
| *Gava*[*^114^*](#_ENREF_114)  *(2019)* | Europe: *Italy* | Primary data collection | 153 | RRMS (105), SPMS (27), PPMS (21) | McDonald 2010 | White | Mean 47.3  (± SD 10.5) | Not pregnant | Mean EDSS 3.1  (± SD 2.2) | Sexual dysfunction | Prospective | Cross-section-al | Yes |
| *Gava*[*^115^*](#_ENREF_115)  *(2014)* | Europe: *Italy* | Primary data collection | 174 | RRMS (141), SPMS (33) | McDonald 2010 | Not reported | Mean  Group 1: 40.8 (± SD 9.5)  Group 2: 47.5 (± SD 13.0*)* | Not pregnant | Mean EDSS Group 1: 2.4 (± SD 1.6)  Group 2: 3.4 (± SD 2.2) | Birth Control | Retrospective | Cohort | No |
| *Geissbuhler*[*^116^*](#_ENREF_116)  *(2020)* | North America, Europe, Asia,  Not reported | Clinical registry & Primary data collection | 831 | MS subtype unspecified (831) | Not reported | White, African, Black, or African-American, Asian, Hispanic, Latino, Central or South American, Not reported, Other | Mean 31 (Range 17-47) | Pregnant  (n=831) | Not reported | Pregnancy  [Pregnancy complicati-ons, DMT exposure], Fetal/  Neonatal Outcomes | Prospective | Cohort | No |
| *Geissbuhler*[*^117^*](#_ENREF_117)  *(2018)* | Not reported | Primary data collection&  Clinical Registry | Not reported | MS subtype unspecified | Not reported | Not reported | Mean  Group 1: 31.7  Group 2: 31.6 | Pregnant  (n=1246) | Not reported | Pregnancy  [Pregnancy complicati-ons, DMT exposure],  Fetal/  Neonatal Outcomes | Prospective & Retrospective | Cohort | No |
| *Ghafoori*[*^118^*](#_ENREF_118)  *(2020)* | Asia: *Iran* | Primary data collection | 25 | MS subtype unspecified (25) | Not reported | Not reported | Mean 31.7  (Range 21- 47) | Pregnant (n=22 children) & Not Pregnant | EDSS Range 1.5-8 | Pregnancy, Fertility, Services around family planning/  pregnancy/  post-pregnancy,  Motherhood/parenthood | Prospective | Cross-section-al | Yes |
| *Gharesi-Fard*[*^119^*](#_ENREF_119)  *(2017)* | Asia: *Iran* | Primary data collection | 20 | RRMS (20) | McDonald 2001 | Not reported | Mean 31  (± SD 8.2) | Not pregnant | Mean EDSS: 1.23 | Sex hormones & MS, Pregnancy  [Mechanism] | Prospective | Case Control | No |
| *Ghiasian*[*^120^*](#_ENREF_120)  *(2020)* | Asia: *Iran* | Primary data collection | 97 | RRMS (97) | McDonald 2010 | Not reported | Mean  Group 1:  25.72  (± SD 3.78)  Group 2: 27.22  (± 6.67) | Pregnant (n=30) & Not pregnant | Mean EDSS Group 1: 0.4 (± SD 0.58)  Group 2: 0.32  (± SD 0.72) | Pregnancy  [MS relapses, Disability outcomes],  Breastfeedi-ng [MS relapses, Disability outcomes] | Prospective | Cohort | No |
| *Giannantoni*[*^121^*](#_ENREF_121)  *(2015)* | Europe | Primary data collection | 31 | RRMS (23), SPMS (4), PPMS (4) | McDonald 2001 | Not reported | Mean 44.5  (± SD 13.7) | Not pregnant | Mean EDSS 4.4 (±SD 2.6) | Sexual dysfunction | Prospective | Cohort | Yes |
| *Giannini*[*^122^*](#_ENREF_122)  *(2012)* | Europe: *Italy* | Primary data collection | 415 | MS subtype unspecified (415) | McDonald 2001 | Not reported | Mean 31.8 | Pregnant  (n=423) | Mean EDSS Group 1: 2  Group 2: 1.3 | Pregnancy  [Pregnancy complicati-ons], DMT exposure, Fetal/  Neonatal Outcomes | Prospective | Cohort | No |
| *Gilli*[*^123^*](#_ENREF_123)  *(2010)* | Europe *Italy* | Primary data collection | 32 | RRMS (32) | McDonald 2005 | Not reported | Mean 33.1  (± SD 41.5) | Pregnant (n=32) | Not reported | Pregnancy  [Mechanism] | Prospective | Cohort | No |
| *Gilmore*[*^124^*](#_ENREF_124)  *(2004)* | North America: *United States* | Primary data collection | 4 | RRMS (4) | Poser | Not reported | Mean 33  (± SD 1.4) | Pregnant (n=4) | Not reported | Pregnancy  [Mechanism] | Prospective | Cohort | No |
| *Giovannoni*[*^125^*](#_ENREF_125)  *(2020)* | Not reported | Clinical registry,  Primary data collection | 62 | MS subtype unspecified (62) | McDonald 2005 | Not reported | Mean  Group 1: 37.6 (±SD 9.8)  Group 2: 37.7 (±SD 10.1) | Pregnant  (n=70) | Not reported | Pregnancy  [Pregnancy complicati-ons, DMT exposure],  Fetal/  Neonatal Outcomes | Prospective | Cohort | No |
| *Gold*[*^126^*](#_ENREF_126)  *(2015)* | Not reported | Primary data collection | 63 | Not reported (63) | Not reported | Not reported | Not reported | Pregnant (n=45) | Not reported | Pregnancy  [Pregnancy complicati-ons] | Prospective | Cohort | No |
| *Gold*[*^127^*](#_ENREF_127)  *(2016)* | Not reported | Primary data collection | 40 | RRMS (40) | McDonald 2005 | Not reported | Age Range  22-38 | Pregnant  (n=45) | Not reported | Pregnancy, Fetal/  Neonatal Outcomes | Prospective | Cohort | No |
| *Goldacre*[*^128^*](#_ENREF_128)  *(2017)* | Europe: *United Kingdom* | Administ-rative data | 98 | MS subtype unspecified (98) | ICD-10 code | Not reported | Age Range  14-49 | Pregnant (n=181) | Not reported | Pregnancy  [Pregnancy complicati-ons], Fetal/  Neonatal Outcomes | Retrospective | Case Control | No |
| *Graves*[*^129^*](#_ENREF_129)  *(2018)* | North America: *United States* | Primary data collection | 415 | CIS/ MS subtype unspecified (415) | McDonald 2005 & 2010 | Not reported | Mean 42.6 (Range 23-63) | Not pregnant | Median EDSS 1.5  (Range  0- 6.5) | Menopause, Sex hormones & MS | Prospective | Case Control | No |
| *Graziano*[*^130^*](#_ENREF_130)  *(2020)* | Europe: *Italy* | Primary data collection | 74 | RRMS (74) | Not reported | Not reported | Mean 37.7  (Range 19-57) | Not pregnant | EDSS Range 0-4 | Motherhood/parenthood | Prospective | Cross-section-al | Yes |
| *Grinsted*[*^131^*](#_ENREF_131)  *(1989)* | Europe: *Denmark* | Primary data collection | 14 | RRMS (5), MS other subtype (9) | Schumac-her | Not reported | Mean 36.4 (Range 24-45) | Not pregnant | DSS Range 2-7 | Menstruati-on, Sex hormones & MS | Prospective | Case Control | No |
| *Gulick*[*^132^*](#_ENREF_132)  *(2007)* | North America | Primary data collection | 172 | RRMS (165), MS other subtype (7) | Not reported | White, Hispanic, Latino, Central or South American, Indigen-ous,  African, Black, or African-American, Asian | Mean 32.66 | Pregnant (not reported) | ADL scale for persons with MS Mean 65.21 (±SD 8.67) | Motherhood/parenthood | Prospective | Cohort | Yes |
| *Gulick*[*^133^*](#_ENREF_133)  *(2003)* | North America | Primary data collection | 174 | MS subtype unspecified (174) | Not reported | White, African, Black, or African-American, Hispanic, Latino, Central or South American,Indigen-ous,  Asian | Mean 32.68 | Pregnant (not reported) | Not reported | Motherhood/parenthood | Prospective | Cohort | Yes |
| *Gulick*[*^134^*](#_ENREF_134)  *(2004)* | North America | Primary data collection | 175 | RRMS (159), MS subtype unspecified (16) | Not reported | White, Not reported | Mean  Group 1: 33.1  (± SD 4)  Group 2: 30.7  (± SD 4.6) | Pregnant (not reported) | Not reported | Child health/  Developme-ntal outcomes | Prospective | Cohort | No |
| *Gulick*[*^135^*](#_ENREF_135)  *(2004)* | North America | Primary data collection | 174 | RRMS (166), SPMS/ PPMS (8) | Not reported | White, African, Black, or African-American, Asian, Hispanic, Latino, Central or South American, Indigen-ous | Mean 32.7 | Pregnant (not reported) | Not reported | Motherhood/parenthood | Prospective | Cohort | Yes |
| *Guven*[*^136^*](#_ENREF_136)  *(2019)* | Asia: *Turkey* | Primary data collection | 47 | MS subtype unspecified (47) | McDonald 2010 | Not reported | Mean 31.1  (± SD 7.12) | Not pregnant | Mean EDSS 1.44  (± SD 1.18) | Menstruati-on | Prospective | Case Control | No |
| *Haas*[*^137^*](#_ENREF_137)  *(2000)* | Europe: *Germany* | Primary data collection | 43 | MS subtype unspecified (43) | Not reported | Not reported | Mean 31  (Range 18-41) | Pregnant (n=43) | Mean EDDS 1.9 | Pregnancy  [MS relapses, DMT exposure] | Prospective | Cohort | No |
| *Hakkarainen*[*^138^*](#_ENREF_138)  *(2020)* | Europe: *Sweden, Finland* | Clinical registry | 1983 | MS subtype unspecified (1983) | Not reported | Not reported | Mean  Group 1: 31.2 (± SD 4.7)  Group 2: 30.6  (± SD 4.5)  Group 3: 31.8  (± SD 4.7)  Group 4: 31.6  (± SD 4.8)  (Overall Range 17-57) | Pregnant (n=2831) | Not reported | Pregnancy  [Pregnancy complicati-ons, DMT exposure] | Retrospective | Case Control | No |
| *Harazim*[*^139^*](#_ENREF_139)  *(2018)* | Europe | Primary data collection | 65 | RRMS (65) | McDonald 2001, Poser,  Polman 2011 | Not reported | Mean  Group 1: 28.5 (± SD 4.1)  Group 2: 30.6 (± SD 4.4) | Pregnant (n=70) | Not reported | Pregnancy  [Pregnancy complicati-ons, MS relapses, DMT exposure, Anesthesia], Fetal/  Neonatal Outcomes | Retrospective | Cohort | No |
| *Harrison*[*^140^*](#_ENREF_140)  *(2002)* | Not reported | Primary data collection | 201 | RRMS (97), SPMS/ PPMS (64), MS other subtype (35) | Not reported | White, African, Black, or African-American, Hispanic, Latino, Central or South American | Mean 43  (Range 22-74) | Not pregnant | Incapacity Status Scale Mean 15.57  (Range 1-41) | Motherhood/parenthood | Retrospective | Cross-section-al | Yes |
| *Hellwig*[*^141^*](#_ENREF_141)  *(2009)* | Europe: *Germany* | Clinical registry & Primary data collection | 124 | RRMS (118), SPMS (6) | Not reported | Not reported | Mean  Group 1: 31.4  (± SD 4.35)  Group 2: 30.3  (± SD 4.33)  Group 3: 29.9  (± SD 2.78) | Pregnant (n=124) | EDSS >4  Group 1 n=7/51  Group 2 n=4/51  Group 3 n=1/22 | Pregnancy  [MS relapses] | Prospective & Retrospective | Case Control | No |
| *Hellwig*[*^142^*](#_ENREF_142)  *(2020)* | North America: *United States, Canada* South America: *Brazil* Europe: *Spain, Russia, Poland, Greece, United Kingdom, Germany, France, Belgium* Asia: *Turkey, Israel, Korea* Africa: *South Africa* | Clinical registry | Not reported | MS subtype unspecified | Not reported | Not reported | Not reported | Pregnant (n=1348) | Not reported | Pregnancy  [Pregnancy complicati-ons, DMT exposure], Fetal/  Neonatal Outcomes | Prospective | Cohort | No |
| *Hellwig*[*^143^*](#_ENREF_143)  *(2020)* | Europe | Clinical registry | Not reported | MS subtype unspecified | Confirmed by health care provider | Not reported | Not reported | Pregnant (n=948) | Not reported | Pregnancy  [DMT exposure], Fetal/  Neonatal Outcomes | Prospective | Cohort | No |
| *Hellwig*[*^144^*](#_ENREF_144)  *(2012)* | Europe: *Germany* | Clinical registry & Primary data collection | Not reported | RRMS | Not reported | Not reported | Mean  Group 1: 31.01  Group 2: 31.03  Group 3: 31.29 | Pregnant (n=335) | Not reported | Pregnancy  [MS relapses, DMT exposure], Breastfeedi-ng, Fetal/  Neonatal Outcomes | Prospective & Retrospective | Cohort | No |
| *Hellwig*[*^145^*](#_ENREF_145)  *(2015)* | Europe: *Germany* | Clinical registry | 201 | RRMS (201) | McDonald 2005 | Not reported | Mean  Group 1: 31.9  Group 2: 30.4 | Pregnant (n=201) | Not reported | Breastfeedi-ng [MS relapses] | Prospective | Cohort | No |
| *Herbstritt*[*^146^*](#_ENREF_146)  *(2016)* | Europe: *Germany* | Clinical registry | 246 | RRMS (246) | McDonald 2005 | Not reported | Mean  Group 1: 31.91  Group 2: 32.2 | Pregnant (n=246) | Not reported | Pregnancy  [DMT exposure], Fetal/  Neonatal Outcomes | Prospective | Cohort | No |
| *Hocaloski*[*^147^*](#_ENREF_147)  *(2016)* | North America *Canada* | Primary data collection | 6 | MS subtype unspecified (6) | Not reported | White | Mean 47.9  (±SD 11.2)  *Combined for patients with MS and spinal cord injury | Not pregnant | Descriptive: Range ambulatory- dependent for transfers | Sexual dysfunction | Prospective | Cohort | Yes |
| *Hoffmann*[*^148^*](#_ENREF_148)  *(2018)* | Europe: *Germany* | Medical records review | 23 | RRMS (23) | Not reported | Not reported | Mean 30.5  (± SD 3.9, Range 21-36) | Pregnant (n=23) | EDSS Range 1-8.5 | Pregnancy  [MS relapses , Disability outcomes] | Retrospective | Cohort | No |
| *Horvat*[*^149^*](#_ENREF_149)  *(2020)* | Europe: *Slovenia, Croatia* | Medical records review | 112 | RRMS (112) | Not reported | Not reported | Mean 31.7 | Pregnant  (n=132) | EDSS Range 0-6.5 | Pregnancy  [MS relapses, DMT exposure] | Retrospective | Cohort | No |
| *Hosl*[*^150^*](#_ENREF_150)  *(2018)* | Europe: *Germany* | Primary data collection | 83 | RRMS (76), SPMS (6), PPMS (1) | McDonald 2010 | Not reported | Median 36.2  (Range 19-65) | Not pregnant | EDSS Range 1-6 | Sexual dysfunction | Prospective | Cross-section-al | Yes |
| *Houtchens*[*^151^*](#_ENREF_151)  *(2020)* | North America: *United States* | Primary data collection  & Medical records review | 248 | CIS (9), RRMS (111), SPMS (3) | McDonald 2005 | Not reported | Mean  Group 1: 32.8  Group 2: 32.2 (Overall range 18-44) | Pregnant (n=123) & Not pregnant | EDSS Range 0-6.5 | Pregnancy  [MS relapses, Disability outcomes, MRI outcomes] | Retrospective | Cohort | No |
| *Houtchens*[*^152^*](#_ENREF_152)  *(2020)* | North America: *United States* | Administr-ative data | 96,937 | MS subtype unspecified (96,937) | Not reported | Not reported | Mean 42  (± SD 9) | Pregnant (n=4843 live births) | Not reported | Pregnancy  [Pregnancy complicati-ons], Fertility, Assisted Reproducti-on | Retrospective | Cohort | No |
| *Hughes*[*^153^*](#_ENREF_153)  *(2014)* | North America, South America, Europe, Australia/New Zealand | Clinical registry | 674 | CIS (18),  RRMS (780), SPMS (75), PPMS (12), unspecified (12) | Poser & McDonald (year not reported) | Not reported | Median 24.4 (IQR 20.9-27.9) | Pregnant (n=893) | Median 1.5 (IQR 0.5-2.0) | Pregnancy  [MS relapses, Disability outcomes] | Prospective | Case Control | No |
| *Hulter*[*^154^*](#_ENREF_154)  *(1995)* | Europe: *Sweden* | Primary data collection | 47 | MS subtype unspecified (47) | Not reported | Not reported | Age Range  21-55 | Not pregnant | EDSS Range 1-9 | Sexual dysfunction | Retrospective | Cross-section-al | Yes |
| *Jawahar*[*^155^*](#_ENREF_155)  *(2014)* | North America: *United States* | Primary data collection | 251 | MS subtype unspecified (251) | -Self-reported | White, African, Black, or African-American, Hispanic, Latino, Central or South American | Age Range  50-79 | Not pregnant | Not reported | Menopause  [Symptoms] | Retrospective | Cohort | Yes |
| *Jesus-Ribeiro*[*^156^*](#_ENREF_156)  *(2017)* | Europe: *Portugal* | Primary data collection & Medical records review | 97 | MS subtype unspecified (97) | McDonald 2010 | Not reported | Mean 31.9  (± SD 4.9) | Pregnant (n=127) | Mean EDSS 1.6 (±SD 0.7) | Pregnancy  [Pregnancy complications, MS relapses, Disability outcomes, DMT exposure, Anesthesia], Breastfeedi-ng [MS relapses], Fetal/  Neonatal Outcomes | Retrospective | Cohort | No |
| *Jolving*[*^157^*](#_ENREF_157)  *(2020)* | Europe: *Denmark* | Administr-ative data | 815 | Not reported (815) | Not reported | Not reported | Median 34 | Pregnant (n=498 live births) | Not reported | Assisted Reproducti-on | Retrospective | Cohort | No |
| *Kamm*[*^158^*](#_ENREF_158)  *(2018)* | Europe: *Switzerla-nd* | Primary data collection | 271 | CIS (7), RRMS (249),  SPMS (6), MS subtype unspecified (9) | McDonald 2010 | Not reported | Age Range  18-55 | Pregnant (n=4) & Not pregnant | Not reported | Pregnancy  [DMT exposure], Services around family planning/  pregnancy/  post-pregnancy | Retrospective | Cross-section-al | Yes |
| *Karlsson*[*^159^*](#_ENREF_159)  *(2014)* | Not reported | Primary data collection | 74 | MS subtype unspecified (74) | Not reported | Not reported | Not reported | Pregnant (n=74) | Not reported | Pregnancy  [Pregnancy complicati-ons, DMT exposure], Fetal/  Neonatal Outcomes | Prospective | Cohort | No |
| *Karp*[*^160^*](#_ENREF_160)  *(2014)* | North America: *Canada* | Medical records review | 588 | RRMS (588) | Not reported | Not reported | Mean  Group 1: 30.7  Group 2: 31.5 | Pregnant (n=254) & Not pregnant | Mean EDSS  Group 1: 0.98  Group 2: 0.72 | Pregnancy  [MS relapses , Disability outcomes] | Retrospective | Cohort | No |
| *Kasatkin*[*^161^*](#_ENREF_161)  *(2018)* | Europe: *Russia* | Primary data collection & Medical records review | 30 | RRMS (30) | McDonald 2005 | Not reported | Not reported | Pregnant (n=30) | Not reported | Pregnancy  [DMT exposure], Fetal/  Neonatal Outcomes, Child health/  Developme-ntal outcomes | Prospective & Retrospective | Case Control | No |
| *Kelly*[*^162^*](#_ENREF_162)  *(2009)* | North America: *United States* | Administ-rative data | 7697 | MS subtype unspecified (7697) | ICD 9 code | White, African, Black, or African-American, Asian, Hispanic, Latino, Central or South American, Other | Mean 31.6 | Pregnant (n=10055) | Not reported | Pregnancy  [Pregnancy complicati-ons], Fetal/  Neonatal Outcomes | Retrospective | Cohort | No |
| *Kempe*[*^163^*](#_ENREF_163)  *(2018)* | Europe: *Sweden* | Primary data collection | 60 | RRMS (60) | McDonald 2010 | Not reported | Mean  Group 1: 36  Group 2: 27  (Overall range 22-40) | Not pregnant | Median EDSS 1.5 (Range 0-3.5) | Sex hormones & MS | Prospective | Case Control | No |
| *Kempe*[*^164^*](#_ENREF_164)  *(2015)* | Europe: *Sweden* | Primary data collection | 22 | Not reported (22) | Physician *reported* | Not reported | Mean 34  (Range 24-44) | Not pregnant | Mean EDSS 1.75  (Range 0-5) | Birth Control, Sex hormones & MS | Prospective | Cohort | Yes |
| *Kieyhanian*[*^165^*](#_ENREF_165)  *(2011)* | Asia: *Iran* | Primary data collection | 1409 | MS subtype unspecified (1409) | McDonald 2005 | Asian | Range 15-41 *15* | Pregnant (n=102) & Not pregnant | Mean EDSS 1.35  (± SD 0.62) | Pregnancy  [MS relapses, Disability outcomes] | Prospective | Cohort | No |
| *Khalid*[*^166^*](#_ENREF_166)  *(2018)* | North America: *United States* | Primary data collection | 16 | CIS (1), RRMS (15) | McDonald 2005 | Not reported | Mean 33  (± SD 4.1) | Pregnant (n=16) | Mean EDSS 1.0  (± SD 1.0) | Pregnancy  [Disability outcomes, MRI outcomes, DMT exposure] | Retrospective | Cohort | No |
| *Khayambashi*[*^167^*](#_ENREF_167)  *(2020)* | North America: *United States* | Clinical registry | 4436 | Not reported | Not reported | White*,* Not reported | Mean 60.5  *Combined for all genders or sexes | Not pregnant | Patient-Determined Disease Steps: Mild-Severe  *Combined for all genders | Gender Identity, Sexual Orientation | Retrospective | Cohort | Yes |
| *Kieseier*[*^168^*](#_ENREF_168)  *(2014)* | Not reported | Clinical registry | 72 | RRMS (72) | Not reported | Not reported | Not reported | Pregnant (n=83) | Not reported | Pregnancy  [DMT exposure], Fetal/  Neonatal Outcomes | Retrospective | Case Series | No |
| *Kilic*[*^169^*](#_ENREF_169)  *(2012)* | Asia: *Turkey* | Primary data collection | 23 | MS subtype unspecified (23) | Not reported | Not reported | Mean 39.83  (± SD 8.88, Range 26-59) | Not pregnant | Not reported | Sexual dysfunction | Prospective | Cross-section-al | Yes |
| *Kinga*[*^170^*](#_ENREF_170)  *(2015)* | Europe: *Romania* | Primary data collection | 36 | RRMS (36) | Not reported | Not reported | Mean 37  (Range 29-37) | Not pregnant | Mean EDSS 1.3 | Sex hormones & MS,  Birth Control | Prospective | Cohort | No |
| *Kleerekooper*[*^171^*](#_ENREF_171)  *(2017)* | Europe: *Netherla-nds* | Medical records review | 17 | RRMS (17) | Not reported | Not reported | Mean 30.5 (Range 21-39) | Pregnant (n=22) | EDSS Range 1.5-6.5 | Pregnancy  [MS relapses, Disability outcomes, MRI outcomes, DMT exposure] | Retrospective | Cohort | No |
| *Koch*[*^172^*](#_ENREF_172)  *(2009)* | Europe: *Netherla-nds* | Clinical registry | 277 | RRMS (277) | Poser | Not reported | Median 27 (IQR 23-34) | Pregnant (n=80 parous women) & Not pregnant | Not reported | Pregnancy  [Disability outcomes] | Retrospective | Cohort | No |
| *Koch*[*^173^*](#_ENREF_173)  *(1999)* | Australia/New Zealand | Primary data collection | 8 | MS subtype unspecified (8) | Self-reported | Not reported | Age Range  38-75 | Not pregnant | Not reported | Sexual dysfunction | Prospective | Cross-section-al | No |
| *Koch*[*^174^*](#_ENREF_174)  *(2002)* | Australia/New Zealand | Primary data collection | 12 | MS subtype unspecified (12) | Not reported | Not reported | Age Range  30-60 | Not pregnant | Not reported | Sexual dysfunction | Retrospective | Cross-section-al | No |
| *Koltuniuk*[*^175^*](#_ENREF_175)  *(2020)* | Europe: *Poland* | Primary data collection | 101 | RRMS (82),  SPMS (14), PPMS (5) | McDonald 2017 | Not reported | Mean 36.7  (± SD 9.56) | Not pregnant | Not reported | Sexual dysfunction | Prospective | Cross-section-al | Yes |
| *Tzitzika*[*^176^*](#_ENREF_176)  *(2018)* | Europe: *Greece* | Primary data collection | 248 | Not reported (248) | Not reported | Not reported | Mean 45.84 | Not pregnant | EDDSS 0-2.5 n= 9,  EDSS 3-5 n=128,  EDSS ≥5.5 n= 24 | Sexual dysfunction | Prospective | Cross-section-al | Yes |
| *Kosmala-Anderson*[*^177^*](#_ENREF_177)  *(2013)* | Europe: *United Kingdom* | Primary data collection | 9 | RRMS (8), SPMS (1) | Not reported | Not reported | Mean 30.6 (Range 23-37) | Pregnant (n=6) & Not pregnant | Not reported | Pregnancy, Motherhood/parenthood | Prospective | Cross-section-al | Yes |
| *Kralik*[*^178^*](#_ENREF_178)  *(2003)* | Australia/New Zealand | Primary data collection | 12 | MS subtype unspecified (12) | Not reported | Not reported | Age Range  30-60 | Not pregnant | Not reported | Sexual dysfunction | Prospective | Cross-section-al | Yes |
| *De Giglio*[*^179^*](#_ENREF_179)  *(2020)* | Europe: *Italy* | Primary data collection | 157 | MS subtype unspecified (157) | Not reported | Not reported | Mean 33  (± SD 5.8) | Not pregnant | Not reported | Pregnancy  [Pregnancy complicati-ons], Fetal/  Neonatal Outcomes | Retrospective | Cross-section-al | No |
| *Labarrere*[*^180^*](#_ENREF_180)  *(1986)* | South America: *Argentina* | Primary data collection | *15 (1 with MS)* | MS subtype unspecified (1) | *Bartel 1983* | Not reported | -Not reported | Pregnant (n=1) | Not reported | Fetal/  Neonatal Outcomes | Retrospective | Case Control | No |
| *Ladeira*[*^181^*](#_ENREF_181)  *(2018)* | Europe: *Portugal* | Primary data collection & Medical records review | 37 | RRMS (28), SPMS (2), PPMS (7) | Not reported | Not reported | Mean 49.8 | Not pregnant | Mean EDSS 2.0 | Menopause  [MS relapses, Disability outcomes, MRI outcomes] | Retrospective | Cohort | No |
| *Lai^182^*  *(2018)* | Asia: *China* | Clinical registry | 55 | MS subtype unspecified (55) | McDonald 2010 | Not reported | Mean 37.9  (±SD 7.6, Range 23-54) | Pregnant (n=126) & Not pregnant | Mean EDSS 0.77  (± SD 1.35) | Pregnancy  [MS relapses , Disability outcomes] | Prospective | Cohort | No |
| *Landi*[*^183^*](#_ENREF_183)  *(2018)* | Europe | Medical records review | 153 | RRMS (153) | Not reported | Not reported | Mean  Group 1: 34.8  Group 2: 36.4  Group 3: 36.0 | Pregnant (n=188) | Mean EDSS  Group 1: 1.34  Group 2: 1.45  Group 3: 1.50 | Pregnancy  [Pregnancy complicati-ons, MS relapses, Disability outcomes, MRI outcomes] | Retrospective | Case Series | No |
| *Langer-Gould*[*^184^*](#_ENREF_184)  *(2020)* | North America: *United States* | Clinical registry | 375 | CIS (69), RRMS (389),  PRMS (4), other (5) | McDonald 2010 | White, African, Black, or African-American, Asian, Other | Mean 31.89 (± SD 4.73) | Pregnant (n=466) | Descriptive Range  No disability- wheelchair required | Pregnancy  [MS relapses, DMT exposure], Breastfeedi-ng [MS relapses, DMT exposure] | Retrospective | Cohort | No |
| *Langer-Gould*[*^185^*](#_ENREF_185)  *(2010)* | North America: *United States* | Primary data collection | 26 | RRMS (26) | Not reported | Not reported | Age Range  Group 1:  24.5- 41.1  Group 2:  26.5-37.1 | Pregnant  (n=26) | EDSS ≤2  n= 20,  EDSS ≥4 n=2 | Pregnancy  [MS relapses, Mechanism] | Prospective | Case Control | No |
| *Langer-Gould*[*^186^*](#_ENREF_186)  *(2011)* | North America: *United States* | Primary data collection | 32 | MS subtype unspecified (32) | Not reported | White & Not reported | Mean  Group 1: 33.0  (± SD 3.5)  Group 2: 32.2  (± SD 4.5) | Pregnant  (n-28) | Not reported | Pregnancy  [MS relapses ,Mechanism], Breastfee- ding | Prospective | Case Control | No |
| *Langer-Gould*[*^187^*](#_ENREF_187)  *(2009)* | North America: *United States* | Primary data collection | 32 | Not reported (32) | Poser | Not reported | Mean 32.5 | Pregnant (n=32) | EDSS ≤2  n= 27,  EDSS ≥4 n=2 | Breastfeedi-ng [MS relapses] | Prospective | Case Control | No |
| *Laplaud*[*^188^*](#_ENREF_188)  *(2006)* | Europe: *France* | Clinical registry & Medical records review | 4 | RRMS (4) | McDonald 2001 | Not reported | Age Range  19-29 | Pregnant  (n=4) | Not reported | Assisted Reproducti-on | Retrospective | Case Series | No |
| *Lavie*[*^189^*](#_ENREF_189)  *(2019)* | Europe: *France, Italy* | Clinical registry | 389 | RRMS, SPMS, MS subtype unspecified  (breakdown not reported | McDonald 2001 & Poser | Not reported | Median 31.4  (IQR 28.4-34.2) | Pregnant  (n=389) | DSS 3: n=56  DSS unknown:  n=9 | Pregnancy  [Pregnancy complicati-ons, MS relapses, Anesthesia] | Retrospective | Cohort | No |
| *Lavorgna*[*^190^*](#_ENREF_190)  *(2019)* | Europe: *Italy* | Primary data collection | 345 | MS other subtype (345) | Not reported | Not reported | Mean 40.8  (± SD 10.5) | Not pregnant | Ability to walk 500m without rest or assistance: 60% | Services around family planning/  pregnancy/  post-pregnancy | Retrospective | Cross-section-al | No |
| *Lavorgna*[*^191^*](#_ENREF_191)  *(2017)* | Europe: *Italy* | Primary data collection | 192 | MS other subtype (192) | Self-reported | Not reported | Mean  Group 1: 35.2  (± SD 8.7)  Group 2: 39.8  (± SD 9.8) | Not pregnant | Not reported | Sexual Orientation | Prospective | Cross-section-al | Yes |
| *Lebrun*[*^192^*](#_ENREF_192)  *(2012)* | Europe: *France* | Primary data collection | 60 | RIS (60) | Okuda criteria & Barkhof-TintorÃ© criteria | Not reported | Mean 30.2  (Range 22-46) | Pregnant (n=7) & Not pregnant | Not reported | Pregnancy  [MS relapses, MRI outcomes] | Prospective | Cohort | No |
| *Lew-Starowicz*[*^193^*](#_ENREF_193)  *(2013)* | Europe: *Poland* | Primary data collection & Medical records review | 137 | MS subtype unspecified (137) | McDonald (year not reported) | Not reported | Mean 50.7  (± SD 7.0) | Not pregnant | Mean EDSS 5.2  (± SD 0.2) | Sexual dysfunction | Retrospective | Cross-section-al | Yes |
| *Lew-Starowicz*[*^194^*](#_ENREF_194)  *(2014)* | Europe | Primary data collection | 137 | RRMS, SPMS, PPMS  (not stratified by gender) | McDonald 2010 | Not reported | Mean 50.7  (± SD 16.4) | Not pregnant | Mean EDDS 5.2(±SD 2.1) | Sexual dysfunction | Prospective | Cross-section-al | Yes |
| *Linssen*[*^195^*](#_ENREF_195)  *(1987)* | Europe: *Netherla-nds* | Primary data collection | 38 | MS subtype unspecified (38) | Not reported | Not reported | Mean 32 | Pregnant (n=19) & Not pregnant | Not reported | Menstruati-on,  Pregnancy  [Disability outcomes] | Retrospective | Cross-section-al | Yes |
| *Lombardi*[*^196^*](#_ENREF_196)  *(2016)* | Europe: *Italy* | Primary data collection | 55 | MS subtype unspecified (55) | Diagnosed by a neurologi-st | Not reported | Mean 34.7  (Range 26-44) | Not pregnant | Mean EDSS 2.9  (Range 1.5-6) | Sexual dysfunction, Sex hormones & MS | Prospective & Retrospective | Cohort | Yes |
| *Lopez*[*^197^*](#_ENREF_197)  *(2006)* | Europe: *Spain* | Primary data collection | 7 | RRMS (7) | Poser | Not reported | Mean 31.9  (± SD 5.1) | Pregnant (n=7) | Median EDSS 1.0 | Pregnancy  [Mechanism] | Prospective | Cohort | No |
| *Lu*[*^198^*](#_ENREF_198)  *(2012)* | North America: *Canada* | Clinical registry | 311 | RRMS (311) | McDonald 2001 & Poser | Not reported | Mean  Group 1: 34.2  (Range 27-42)  Group 2: 33.0  (Range 26-40)  Group 3: 33.3  (Range 26-42)  Group 4: 32.2  (Range 21-44) | Pregnant (n=418) | EDSS Range  Group 1:  0-6  Group 2:  0-6.5  Group 3:  0-6.5  Group 4:  0-7 | Pregnancy  [DMT exposure], Fetal/  Neonatal Outcomes | Retrospective | Cohort | No |
| *Lu*[*^199^*](#_ENREF_199)  *(2013)* | North America: *Canada* | Clinical registry | 550 | MS subtype unspecified (55) | McDonald 2001 & 2005, Poser | Not reported | Mean 32  (Range 19-44) | Pregnant (n=762) | EDSS Range  0 -≥3 | Pregnancy  [Pregnancy complicati-ons], Fetal/  Neonatal Outcomes | Retrospective | Cohort | No |
| Lu[^200^](#_ENREF_200)  (2013) | North America: *Canada* | Administ-rative data, Clinical registry | 290 | RRMS (282),  SPMS (4) unknown (4) | McDonald 2005, Poser | Not reported | Median 32  (Range 19-44) | Pregnant  (n=381) | Median EDSS 1.5 (Range 0- 7.5) | Pregnancy  [Pregnancy complicati-ons],  Fetal/  Neonatal Outcomes | Retrospective | Cohort | No |
| *Lu*[*^201^*](#_ENREF_201)  *(2013)* | North America: *Canada* | Clinical registry, Administ-rative data | 321 | RRMS (303), SPMS (18), PPMS (9) | McDonald 2005 & Poser | Not reported | Age Range  19-44 | Parrous  (n=302) & pregnant | Median EDSS 1.5 (Range 0- 7.5) | Pregnancy  [Pregnancy complicati-ons, Disability outcomes, Anesthesia], Fetal/  Neonatal Outcomes | Retrospective | Cohort | No |
| *MacDonald*[*^202^*](#_ENREF_202)  *(2018)* | North America: *United States* | Administ-rative data | Not reported | MS subtype unspecified | Not reported | Not reported | Age Range  12-55 | Pregnant (n=974) | Not reported | Pregnancy  [Pregnancy complicati-ons] | Retrospective | Cohort | No |
| *MacDonald*[*^203^*](#_ENREF_203)  *(2019)* | North America: *United States* | Administ-rative data | 1432 | Not reported (4132) | ICD-9 code | Not reported | Mean  Group 1: 33.1  Group 2: 33.2 | Pregnant (n=1649) | Not reported | Pregnancy  [Pregnancy complicati-ons, DMT exposure] | Retrospective | Cohort | No |
| *MacDonald*[*^204^*](#_ENREF_204)  *(2019)* | North America: *United States* | Administ-rative data | 3875 | MS subtype unspecified (3875) | Not reported | White, African, Black, or African-American, Asian, Hispanic, Latino, Central or South American, Indigen-ous, Other | Mean  Group 1: 32.6  Group 2: 31.5 | Pregnant (not reported) | Not reported | Pregnancy  [Pregnancy complicati-ons, MS relapses] | Retrospective | Cohort | No |
| *Maciejewska*[*^205^*](#_ENREF_205)  *(2016)* | Not reported | Primary data collection | 20 | MS subtype unspecified (20) | Patients self-reported diagnosis | Not reported | Mean 40  (Range 20-56) | Not pregnant | Not reported | Fertility, Menstruati-on | Retrospective | Cross-section-al | Yes |
| *MacKenzie-Graham*[*^206^*](#_ENREF_206)  *(2018)* | North America: *United States* | Primary data collection | 111 | RRMS (111) | McDonald 2005 | White, African, Black, or African-American, Hispanic, Latino, Central or South American*,* Other | Mean 37.3  (Range 20-37.3) | Not pregnant | EDSS Range 0- 5.5 | Sex hormones & MS | Prospective | Clinical trial | No |
| *MacLurg*[*^207^*](#_ENREF_207)  *(2004)* | Europe: *United Kingdom* | Medical records review | 112 | MS subtype unspecified (112) | Poser | Not reported | Not reported | Not pregnant | Not reported | Cancer/  Cancer screening | Retrospective | Cohort | No |
| *Mahlanza*[*^208^*](#_ENREF_208)  *(2020)* | North America: *United States* | Primary data collection | 92 | MS subtype unspecified (92) | Not reported | White, African, Black, or African-American ,Hispanic, Latino, Central or South American | Mean 33.6  (± SD 1.8) | Pregnant  (n=65) | Mean EDSS  1.0 (±SD 1.3) | Fetal/  Neonatal Outcomes, Child health/  Developme-ntal outcomes, Pregnancy  [Pregnancy complicati-ons, MS relapses, Disability outcomes, DMT exposure] | Prospective | Cohort | No |
| *Mallucci*[*^209^*](#_ENREF_209)  *(2020)* | Europe: *Italy* | Primary data collection | 9 | RRMS (9) | McDonald 2017 | Not reported | Mean 32.33  (± SD 4.9) | Pregnant (n=9) | EDSS Range  0-3.5 | Pregnancy  [Pregnancy complicati-ons, Mechanism] | Prospective | Case Control | No |
| *Marck*[*^210^*](#_ENREF_210)  *(2016)* | Australia/New Zealand, North America, Europe | Primary data collection | 1663 | RRMS, SPMS, PPMS  (not stratified by gender) | Self-reported | Not reported | Age Range  18-≥49 | Not pregnant | Patient Determined Disease Steps Range None-Severe | Sexual dysfunction | Prospective | Cross-section-al | Yes |
| *Masera*[*^211^*](#_ENREF_211)  *(2015)* | Europe: *Italy* | Primary data collection | 445 | RRMS/ SPMS (445) | McDonald 2005 | Not reported | Mean 25.0  (±SD 7.1) | Pregnant (n=184 parous women) & Not pregnant | Not reported | Pregnancy  [Disability outcomes] | Retrospective | Case Control | No |
| *May*[*^212^*](#_ENREF_212)  *(2008)* | Europe: *United Kingdom* | Clinical registry | 10 | MS subtype unspecified (10) | Not reported | Not reported | Not reported | Pregnant (n=10) | Not reported | Pregnancy  [Anesthesia], Fetal/  Neonatal Outcomes | Retrospective | Cohort | No |
| *Mehrpour*[*^213^*](#_ENREF_213)  *(2018)* | Asia: *Iran* | Primary data collection | 50 | RRMS (50) | Not reported | Not reported | Mean 28.86  (±SD 5.5) | Not pregnant | Mean EDSS 1.80  (±SD 0.95,  Range 0-4.5) | Fertility, Sex hormones & MS | Prospective | Case Control | No |
| *Mehta*[*^214^*](#_ENREF_214)  *(2019)* | Australia/New Zealand | Primary data collection | 47 | Not reported (47) | Not reported | White | Mean 47.1 | Not pregnant | Mean EDSS 2.0 | Pregnancy | Prospective | Cross-section-al | No |
| *Merghati-Khoei*[*^215^*](#_ENREF_215)  *(2013)* | Asia: *Iran* | Primary data collection | 132 | MS subtype unspecified (132) | Not reported | Not reported | Mean 36.9  (Range 20-58) | Not pregnant | Not reported | Sexual dysfunction | Prospective | Cross-section-al | Yes |
| *Michel*[*^216^*](#_ENREF_216)  *(2012)* | Europe: *France* | Clinical registry | 32 | RRMS (27), SPMS (4), PPMS (1) | McDonald 2005 | Not reported | Mean 32.9  (±SD 9.5) | Pregnant (n=21) & Not pregnant | Not reported | Assisted Reproducti-on | Retrospective | Case Series | No |
| *Mirmosayyeb*[*^217^*](#_ENREF_217)  *(2018)* | Asia: *Iran* | Primary data collection | 181 | RRMS (181) | McDonald 2010 | Not reported | Mean 36.04  (±SD 9.86) | Not pregnant | EDSS <3.5  n=130,  EDSS ≥3.5  n=51 | Menarche/  Puberty, Menstruati-on | Retrospective | Cross-section-al | No |
| *Moberg*[*^218^*](#_ENREF_218)  *(2016)* | Europe: *Denmark* | Clinical registry, Administ-rative data | 4177 | MS subtype unspecified (4177) | McDonald 2005,  Poser, Allison and Millar | Not reported | Mean 27.9  (±SD 5.4) | Not pregnant | Not reported | Child health/  Developme-ntal outcomes | Retrospective | Case Control | No |
| *Moberg*[*^219^*](#_ENREF_219)  *(2017)* | Europe: *Denmark* | Clinical registry, Administ-rative data | 1443 | MS subtype unspecified (1443) | McDonald 2005, Poser, Allison and Millar | Not reported | Median 28 (Range 15-54) | Not pregnant | Not reported | Child health/  Developme-ntal outcomes, Motherhood/parenthood | Retrospective | Cohort | No |
| *Mohammadi*[*^220^*](#_ENREF_220)  *(2020)* | Asia: *Iran* | Primary data collection | 117 | RRMS (69), SPMS/ PPMS (48) | McDonald 2005 | Not reported | Mean 5.77 (±SD 8.07) | Not pregnant | Mean EDDS 2.99  (±SD 2.17) | Sexual dysfunction | Prospective | Cross-section-al | Yes |
| *Mueller*[*^221^*](#_ENREF_221)  *(2002)* | North America: *United States* | Administ-rative data | 198 | Not reported (198) | *ICD-9 Code* | White, African, Black, or African-American, Asian, Other | Age  <20 = 2.5% through  ≥40 = 7.6% | Pregnant (n=198) | Not reported | Pregnancy  [Pregnancy complicati-ons],  Fetal/  Neonatal Outcomes | Retrospective | Cohort | No |
| *Nabavi*[*^222^*](#_ENREF_222)  *(2010)* | Asia: *Iran* | Primary data collection | 58 | RRMS (44), SPMS (12), PPMS (2) | McDonald 2005 | Not reported | Mean 31.5  (±SD 7.7) | Not pregnant | EDSS 0- 2 65.5% through  EDSS 4-6  10.3% | Menstruati-on, Sex hormones & MS | Retrospective | Cross-section-al | No |
| *Najafidoulatabad*[*^223^*](#_ENREF_223)  *(2014)* | Asia: *Iran* | Primary data collection | 60 | MS subtype unspecified (60) | Diagnosed with MS by a doctor | Not reported | Mean 31.6  (±SD 8,  Range 18-45) | Not pregnant | Not reported | Sexual dysfunction | Prospective | Clinical trial | Yes |
| *Nasimbera*[*^224^*](#_ENREF_224)  *(2018)* | South America | Primary data collection | 8 | MS subtype unspecified (8) | Not reported | Not reported | Mean 44.7  (±SD 8.55) | Not pregnant | EDSS 0 0%,  EDSS 1-3 8.3%,  EDSS 3.5-5.5 66.7%  EDSS ≥6 25% | Sexual dysfunction | Retrospective | Case Control | Yes |
| *Nelson*[*^225^*](#_ENREF_225)  *(1988)* | North America: *United States* | Primary data collection | 435 | MS subtype unspecified (435) | Schumac-her | Not reported | Not reported | Pregnant (n=215) | Not reported | Pregnancy, Breastfeedi-ng [MS relapses] | Retrospective | Cross-section-al | No |
| *Neuteboom*[*^226^*](#_ENREF_226)  *(2010)* | Europe: *Netherla-nds* | Primary data collection | 15 | RRMS (15) | McDonald 2005 | Not reported | Mean  Group 1: 31.5  Group 2: 30.7 | Pregnant  (n=15) | EDSS Range 0-2.5 | Pregnancy  [Mechanism] | Prospective | Cohort | No |
| *Neuteboom*[*^227^*](#_ENREF_227)  *(2012)* | Europe: *Netherla-nds* | Primary data collection | 35 | RRMS (35) | Not reported | Not reported | Mean 31.2 | Pregnant  (n=35) | EDSS Range  0-4 | Pregnancy  [Pregnancy complicati-ons, MS relapses, Disability outcomes], Fetal/  Neonatal Outcomes | Prospective | Cohort | Yes |
| *Neuteboom*[*^228^*](#_ENREF_228)  *(2009)* | Europe: *Netherla-nds* | Primary data collection | 36 | RRMS (36) | Not reported | White,  Other | Mean 30.9 | Pregnant (n=36) | EDSS Range  0-4 | Pregnancy  [MS relapses, Mechanism] | Prospective | Cohort | No |
| *Neuteboom*  [*^229^*](#_ENREF_229)*(2010)* | Europe: *Netherla-nds* | Primary data collection | 12 | RRMS (12) | Not reported | Not reported | Mean  Group 1: 33.0  Group 2: 32.3 | Pregnant (n=14) | EDSS Range 0-2 | Pregnancy  [Mechanism] | Prospective | Cohort | No |
| *Nguyen*[*^230^*](#_ENREF_230)  *(2019)* | Not reported | Clinical registry | 1178 | RRMS (1156), SPMS (22) | McDonald 2005 & 2010 | Not reported | Median 31.8 | Pregnant (n=1521) | Median EDSS 1.5 | Pregnancy  [Pregnancy complicati-ons, DMT exposure], Fertility | Prospective | Cohort | No |
| *Novo*[*^231^*](#_ENREF_231)  *(2019)* | Europe: *Portugal* | Primary data collection | 97 | RRMS (95), SPMS (1), PPMS (1) | McDonald 2010 | Not reported | Mean 32.5  (±SD 4.3) | Pregnant (n= 90 live births) | Mean EDSS 1 (±SD 2.0, Range 0-6) | Pregnancy  [Pregnancy complicati-ons, MS relapses, Disability outcomes, DMT exposure], Fetal/  Neonatal Outcomes | Retrospective | Cohort | No |
| *Oh*[*^232^*](#_ENREF_232)  *(2020)* | Not reported | Primary data collection | 160 | RRMS (160) | McDonald 2005 | Not reported | Mean 32.6  (Range 21.2-43.6) | Pregnant (n=264) | EDSS Range 0-6.5 | Pregnancy  [Pregnancy complicati-ons, MS relapses, DMT exposure], Fetal/  Neonatal Outcomes | Prospective | Cohort | No |
| *Orasanu*[*^233^*](#_ENREF_233)  *(2013)* | North America: *United States, Canada* | Clinical registry | 5171 | MS subtype unspecified (5171) | Self-reported | White, African, Black, or African-American, Hispanic, Latino, Central or South American, Other | Mean 38.4  (±SD 9.6) | Not pregnant | PDDS median 4.0 | Sexual dysfunction | Retrospective | Cross-section-al | Yes |
| *Orvieto*[*^234^*](#_ENREF_234)  *(1999)* | Asia: *Israel* | Primary data collection | 15 | RRMS (15) | Poser | Not reported | Mean 30.5  (±SD 4.8,  Range 22-37) | Pregnant (n=15) | Not reported | Pregnancy  [MS relapses] | Prospective | Cohort | No |
| *Paavilainen*[*^235^*](#_ENREF_235)  *(2007)* | Europe: *Finland* | Primary data collection | 28 | RRMS (28) | McDonald 2001 | Not reported | Mean 30.2 | Pregnant (n=28) | Mean EDSS 1.2 (±SD 1.1) | Pregnancy  [MS relapses, MRI outcomes] | Prospective | Cohort | No |
| *Paavilainen*[*^236^*](#_ENREF_236)  *(2012)* | Europe: *Finland* | Primary data collection | 19 | RRMS (19) | McDonald 2001 | Not reported | Mean 31.6  (Range 25-41) | Pregnant (n=19) | Mean EDSS 1.24 | Pregnancy  [MS relapses, MRI outcomes] | Prospective | Cohort | No |
| *Paraska*[*^237^*](#_ENREF_237)  *(2012)* | North America: *United States* | Primary data collection | 11 | MS subtype unspecified (11) | Not reported | White, African, Black, or African-American | Age Range  40-70 | Not pregnant | All homebound due to disabiliy | Cancer/  Cancer screening | Retrospective | Cohort | Yes |
| *Parton*[*^238^*](#_ENREF_238)  *(2019)* | Australia/New Zealand | Primary data collection | 20 | RRMS (18), SPMS (2) | Not reported | Indigen-ous,  White, Hispanic, Latino, Central or South American, Asian | Mean 37.5 (Range 26-54) | Not pregnant | Not reported | Motherhood/parenthood | Prospective | Cross-section-al | Yes |
| *Pasic*[*^239^*](#_ENREF_239)  *(2019)* | Europe: *Croatia* | Primary data collection | 75 | RRMS, SPMS, PPMS  (not stratified by gender) | Not reported | Not reported | Mean 42.09  (Range 19-77) | Not pregnant | Mean EDSS 3.1  (Range 0-7) | Sexual dysfunction | Prospective | Cross-section-al | Yes |
| *Pasto*[*^240^*](#_ENREF_240)  *(2012)* | Europe: *Italy* | Primary data collection | 415 | MS subtype unspecified (415) | McDonald 2001 | Not reported | Mean 32.8 | Pregnant (n=349) | Mean EDSS 1.5 | Pregnancy  [MS relapses, Anesthesia] | Prospective | Cohort | No |
| *Patti*[*^241^*](#_ENREF_241)  *(2008)* | Europe: *Italy* | Primary data collection | 38 | RRMS (38) | Not reported | Not reported | Mean 28.9  (±SD 5.4) | Pregnant (n=38) | Mean EDSS 1.5 (±SD 0.6) | Pregnancy  [Pregnancy complicati-ons, DMT exposure], Fetal/  Neonatal Outcomes, Child health/  Developme-ntal outcomes | Retrospective | Cohort | No |
| *Patti*[*^242^*](#_ENREF_242)  *(2014)* | Europe: *Italy* | Medical records review | 11 | RRMS (110 | Evaluated at an MS center | Not reported | Mean 31.6  (±SD 2.7, Range 28-38) | Pregnant (n=11) | Not reported | Pregnancy  [Pregnancy complicati-ons, DMT exposure], Fetal/  Neonatal Outcomes | Retrospective | Cohort | No |
| *Pauliat*[*^243^*](#_ENREF_243)  *(2020)* | Europe: *Switzerla-nd* | Primary data collection | Not reported | RRMS | Not reported | Not reported | Median Group1: 32 (IQR 28-34) Group 2: 32 (29-35) | Pregnant (n=125) | Not reported | Pregnancy  [DMT exposure], Fetal/  Neonatal Outcomes | Prospective | Cohort | No |
| *Payne*[*^244^*](#_ENREF_244)  *(2010)* | Australia/New Zealand | Primary data collection | 9 | RRMS (8), SPMS (1) | Not reported | Indigen-ous, White | Age Range  22-45 | Pregnant (n=16 children) | Independent for all activities n=8, Dependent on another person for activities n=1 | Pregnancy, Breastfeedi-ng, Motherhood/parenthood | Prospective | Cross-sectio-nal | Yes |
| *Pebdani*[*^245^*](#_ENREF_245)  *(2015)* | North America: *United States* | Primary data collection | 391 | RRMS (195), PPMS (11), MS other subtype (104),  Unknown MS subtype (45) | Not reported | White, Not reported | Mean 52  (Range 21-82) | Pregnant (n=302 women who had pregnancies)& Not pregnant | Not reported | Services around family planning/  pregnancy/  post-pregnancy | Prospective | Cross-section-al | No |
| *Pelfrey*[*^246^*](#_ENREF_246)  *(2005)* | North Americ:a *United States* | Primary data collection | 25 | RRMS (25) | McDonald 2001, Poser | Not reported | Mean 37.8  (±SD 8.3) | Not pregnant | Not reported | Sex hormones & MS | Prospective | Cross-section-al | No |
| *Popek*[*^247^*](#_ENREF_247)  *(2018)* | Europe: *Poland* | Primary data collection | 55 | MS subtype unspecified (55) | Not reported | Not reported | Mean 39.47  (Range 20-45) *Combined for all genders or sexes | Not pregnant | EDSS Range (0-10)  *Combined for all genders or sexes | Sexual dysfunction | Prospective | Cross-section-al | Yes |
| *Portaccio*[*^248^*](#_ENREF_248)  *(2011)* | Europe: *Italy* | Primary data collection | 298 | RRMS (298) | McDonald 2001 | Not reported | Mean 31.5  (±SD 4.7) | Pregnant (n=423) | Mean EDSS 1.5 (±SD 1.0) | Breastfeedi-ng [MS relapse] | Prospective | Cohort | No |
| *Portaccio*[*^249^*](#_ENREF_249)*^,^* [*^250^*](#_ENREF_250)  *(2018)* | Europe: *Italy* | Primary data collection | 498 | RRMS (498) | McDonald 2001 & 2010,  Poser | Not reported | Mean  Group 1: 31.2 (±SD 4.9)  Group 2: 31.8 (±SD 4.7) | Pregnant (n=515) | Median EDSS  Group 1:2.4 (IQR 1.5-3.5)  Group 2: 1.5 (IQR 1.0-2.0) | Pregnancy  [MS relapses, Disability outcomes, DMT exposure], Fetal/  Neonatal Outcomes | Prospective | Cohort | No |
| *Portaccio*[*^251^*](#_ENREF_251)  *(2014)* | Europe: *Italy* | Primary data collection | 345 | RRMS (345) | McDonald 2001 | Not reported | Mean 31.8  (±SD 4.7) | Pregnant (n=350) | Median EDSS: 1.5 (IQR 1.0-2.0) | Breastfeedi-ng, Pregnancy [MS relapses, DMT exposure, Disability outcomes] | Prospective | Cohort | No |
| *Poser*[*^252^*](#_ENREF_252)  *(1983)* | Europe: *Germany* | Primary data collection | 512 | MS subtype unspecified (512) | Not reported | Not reported | Age Range  15- ≥60 | Pregnant (n=927) & Not pregnant | DSS divided by duration of disease in years Range 0.2- ≥2 | Pregnancy  [Pregnancy complicati-ons, Disability outcomes], Fetal/  Neonatal Outcomes | Retrospective | Cross-section-al | No |
| *Pozzilli*[*^253^*](#_ENREF_253)  *(2015)* | Europe: *Italy* | Primary data collection | 149 | RRMS (149) | McDonald 2001 | Not reported | Mean  Group 1: 29.8  Group 2: 29.8  Group 3: 29.9 | Not Pregnant | EDSS Range  0-4.5 | Sex hormones & MS | Prospective | Clinical trial | No |
| *Proschmann*[*^254^*](#_ENREF_254)  *(2018)* | Europe: *Germany* | Primary data collection | 11 | RRMS (11) | Not reported | Not reported | Age Range  29-39 | Not pregnant | EDSS Range  1.5-2.5 | Pregnancy, Breastfeedi-ng  [DMT exposure], Fetal/  Neonatal Outcomes | Prospective | Case Series | No |
| *Prunty*[*^255^*](#_ENREF_255)  *(2008)* | Australia/New Zealand | Primary data collection | 20 | RRMS (12), SPMS (2), PPMS (8) | Not reported | Not reported | Mean 32  (Range 20-40) | Not pregnant | Not reported | Motherhood/parenthood | Prospective | Cross-section-al | No |
| *Prunty*[*^256^*](#_ENREF_256)  *(2008)* | Australia/New Zealand: *Australia* | Primary data collection | 194 | RRMS (138), SPMS (2), PPMS (1) | Not reported | Not reported | Mean  Group 1: 31.95 Group 2: 31.10 | Not pregnant | Not reported | Motherhood/parenthood | Prospective | Clinical trial | Yes |
| *Ramagopalan*[*^257^*](#_ENREF_257)  *(2012)* | North America: *Canada* | Clinical registry, Medical records review | 2105 | RRMS (1769), PPMS (119) | McDonald 2001, Poser | Not reported | Mean age *47.7* | Pregnant (not reported)& Not pregnant | Mean EDSS 3.2 | Pregnancy  [Disability outcomes] | Retrospective | Cohort | No |
| *Ramien*[*^258^*](#_ENREF_258)  *(2019)* | Europe: *Germany* | Primary data collection | 11 | MS subtype unspecified (11) | Physician diagnosis | Not reported | Age Range  25-40 | Pregnant (n=11) | Not reported | Pregnancy  [Mechanism] | Prospective | Cohort | No |
| *Rasmussen*[*^259^*](#_ENREF_259)  *(2018)* | Europe: *Denmark* | Primary data collection | 488 | CIS/ RRMS, SPMS/ PPMS  (not stratified by gender) | Self-reported | Not reported | Mean 39 | Not pregnant | Not reported | Services around family planning/  pregnancy/  post-pregnancy | Retrospective | Cross-section-al | Yes |
| *Razaz*[*^260^*](#_ENREF_260)  *(2020)* | Europe: *Sweden* | Clinical registry | 586 | RRMS (546), SPMS (32), PPMS (7), unknown (7) | Not reported | Not reported | Mean  Group 1: 32.7 (±SD 4.2)  Group 2: 31.5 (±SD 4.8) Group 3: 31.1 (±SD 4.8) | Pregnant (n=586) | Mean EDSS Group 1: 1  Group 2: 2  Group 3: 1.5 | Pregnancy  [MS relapses, MRI outcomes] | Retrospective | Cohort | No |
| *Razaz*[*^261^*](#_ENREF_261)  *(2016)* | North America: *Canada* | Administ-rative data | 1028 children of parents with MS | Not reported (1028) | Not reported | Not reported | Mean age of parents 30 | Not pregnant | Not reported | Child health/  Developme-ntal outcomes | Retrospective | Cohort | No |
| *Razaz*[*^262^*](#_ENREF_262)  *(2016)* | North America: *Canada* | Administ-rative data | 543 | Not reported (543) | Not reported | Not reported | Age Range  <25-≥35 | Not pregnant | Not reported | Child health/  Developme-ntal outcomes | Retrospective | Cohort | No |
| *Razaz*[*^263^*](#_ENREF_263)  *(2015)* | North America: *Canada* | Adminis-trative data | 130 | MS subtype unspecified (130) | Not reported | Not reported | Mean 31.3  (±SD 4.9) | Not pregnant | Not reported | Child health/  Developme-ntal outcomes | Retrospective | Cohort | No |
| *Razaz*[*^264^*](#_ENREF_264)  *(2016)* | North America: *Canada* | Administ-rative data | 255 | MS subtype unspecified (255) | ICD codes for MS or disease modifying therapies | Not reported | Mean 37.3 ***Combined for all genders or sexes | Pregnant (n=360) | Not reported | Pregnancy  [Pregnancy complicati-ons], Child health/  Developme-ntal outcomes | Retrospective | Cohort | No |
| *Rezaallah*[*^265^*](#_ENREF_265)  *(2019)* | Not reported | Primary data collection | Not reported | MS subtype unspecified | Not reported | Not reported | Not reported | Pregnant (not reported) &  Not pregnant | Not reported | Pregnancy  [DMT exposure], Breastfeedi-ng  [DMT exposure] | Retrospective | Cross-section-al | No |
| *Rinta*[*^266^*](#_ENREF_266)  *(2010)* | Europe: *Finland* | Primary data collection | 19 | RRMS (19) | Not reported | Not reported | Mean 30.1  (±SD 0.7) | Pregnant (n=19) | Mean EDSS 1.1 (±SD 1.2) | Pregnancy  [MS relapses, Disability outcomes, Mechanism] | Prospective | Cross-section-al | No |
| *Robertson*[*^267^*](#_ENREF_267)  *(2018)* | North America: *United States* | Primary data collection | 11 | RRMS (11) | Not reported | Not reported | Mean 41.13 **Combined for all genders or sexes* | Not pregnant | Not reported | Sexual dysfunction | Prospective | Cohort | Yes |
| *Romero*[*^268^*](#_ENREF_268)  *(2014)* | North America: *United States, Canada,* South America: *Brazil* Europe: *Germany, Belgium, Italy, Poland, Ireland, Spain, United Kingdom, Russia, Hungary, Serbia* Asia: *Turkey* Australia/New Zealand: *Australia* | Clinical registry | Not reported | Not reported | Not reported | Not reported | Not reported | Pregnant (n=423) | Not reported | Pregnancy  [Pregnancy complicati-ons, DMT exposure], Fetal/  Neonatal Outcomes | Prospective | Cohort | No |
| *Rossi*[*^269^*](#_ENREF_269)  *(2018)* | Europe: *Italy* | Primary data collection | 12 | RRMS (12) | McDonald 2010 | White | Age Range  26-37 | Pregnant (n=12) | EDSS Range  1-6 | Pregnancy  [Mechanism], Sex hormones & MS | Prospective | Cohort | No |
| *Roullet*[*^270^*](#_ENREF_270)  *(1993)* | Europe: *France* | Clinical registry | 125 | RRMS (125) | Poser, Schumac-her | Not reported | Mean Group 1: 38.5 Group 2: 43.1 Group 3: 39.4 | Pregnant  (n=32) | Mean EDSS: Group 1: 2.15  Group 2: 3.44  Group 3: 2.51 | Pregnancy  [MS relapses] | Retrospective | Cohort | No |
| *Roux*[*^271^*](#_ENREF_271)  *(2015)* | Europe: *France* | Primary data | 115 | RRMS (65), SPMS (40), PPMS (10) | McDonald 2010 | Not reported | Mean 43.39  (Range 21-78) | Pregnant (n=216) & Not pregnant | Mean EDSS 4.18  (Range 0-7) | Fertility | Retrospective | Cross-section-al | No |
| *Runia*[*^272^*](#_ENREF_272)  *(2015)* | Europe: *Netherla-nds* | Primary data collection | 43 | RRMS (43) | McDonald 2001 | Not reported | Mean 31.5 | Pregnant (n=43) | Median EDSS 1.5 (IQR 1.0-2.0) | Pregnancy  [MS relapses, Mechanism] | Retrospective | Cohort | Yes |
| *Runmarker*[*^273^*](#_ENREF_273)  *(1995)* | Europe: *Sweden* | Primary data collection | 153 | RRMS (108), PPMS (45) | Poser | Not reported | Not reported | Pregnant (not reported) & Not pregnant | Not reported | Pregnancy  [Disability outcomes] | Prospective | Cohort | No |
| *Sadeghi Bahmani*[*^274^*](#_ENREF_274)  *(2020)* | Asia: *Iran* | Primary data collection | 62 | MS subtype unspecified (62) | McDonald 2010 | Not reported | Mean  Group 1: 39.35  Group 2: 40.61  Group 3: 33.77  (Overall range 18-65) | Not pregnant | Highest EDSS 5.5 | Sexual dysfunction | Prospective | Clinical trial | Yes |
| *Sadeghpour*[*^275^*](#_ENREF_275)  *(2020)* | Asia | Primary data collection | 23 | RRMS (23) | McDonald 2017 | Not reported | Mean 29.69  (±SD 5.21) | Not pregnant | Mean EDSS: 0.21  (Range 0-1) | Fertility | Prospective | Cross-section-al | No |
| *Sadovnick*[*^276^*](#_ENREF_276)  *(1994)* | North America: *Canada* | Clinical registry | 117 | MS subtype unspecified (177) | Poser & Schumac-her | Not reported | Not reported | Pregnant (n=53) & Not pregnant | Not reported | Pregnancy  [Pregnancy complicati-ons, MS relapses] | Prospective | Cohort | No |
| *Salemi*[*^277^*](#_ENREF_277)  *(2004)* | Europe: *Italy* | Primary data collection | 70 | RRMS (70), SPMS (0) | Poser | Not reported | Age Range  16-48 | Not pregnant | Not reported | Pregnancy  [MS relapses] | Retrospective | Cross-section-al | No |
| *Salhofer-Polanyi*[*^278^*](#_ENREF_278)  *(2017)* | Europe: *Austria* | Primary data collection | 42 | RRMS (42) | Not reported | Not reported | Mean 37  (±SD 7, Range 18-50) | Not pregnant | Median EDSS 1.75 | Sexual dysfunction | Prospective | Cohort | Yes |
| *Salminen*[*^279^*](#_ENREF_279)  *(2010)* | Europe: *United Kingdom* | Primary data collection | 13 | RRMS (13) | Not reported | Not reported | Mean 31.6  (Range 25-41) | Pregnant (n=14) | Not reported | Pregnancy  [Pregnancy complicati-ons, DMT exposure] Fetal/  Neonatal Outcomes | Prospective | Case Series | No |
| *Sanchez-Ramon*[*^280^*](#_ENREF_280)  *(2005)* | Not reported | Primary data collection | 13 | RRMS (13) | Poser | Not reported | Age Range  26-38 | Pregnant (n=13) | EDSS Range  0-2 | Pregnancy  [Mechanism] | Prospective | Cross-section-al | No |
| *Sandberg-Wollheim*[*^281^*](#_ENREF_281)  *(2005)* | Not reported | Primary data collection | 3361 | CIS (197),  RRMS (2558), SPMS (606) | Poser | Not reported | Mean 39.5  (Range 17-74) | Pregnant (n=69) & Not pregnant | Not reported | Pregnancy  [Pregnancy complicati-ons, DMT exposure] | Prospective | Clinical trial | No |
| *Sandberg-Wollheim*[*^282^*](#_ENREF_282)  *(2011)* | Not reported | Clinical registry | Not reported | MS subtype unspecified | Not reported | Not reported | Not reported | Pregnant (n=1022) | Not reported | Pregnancy  [Pregnancy complicati-ons, DMT exposure], Fetal/  Neonatal Outcomes | Prospective & Retrospective | Cohort | No |
| *Saraste*[*^283^*](#_ENREF_283)  *(2018)* | Europe: *Finland* | Primary data collection | 59 | RRMS (59) | Not reported | Not reported | Mean 30.5  (±SD 4.2) | Pregnant  (49) | Not reported | Pregnancy | Prospective | Cohort | No |
| *Saraste*[*^284^*](#_ENREF_284)  *(2006)* | Europe: *Finland* | Primary data collection | 6 | RRMS (6) | Not reported | Not reported | Mean 26 | Pregnant (n=6) | Not reported | Pregnancy  [Mechanism] | Prospective | Case Series | No |
| *Saraste*[*^285^*](#_ENREF_285)  *(2007)* | Europe: *Finland* | Primary data collection | 60 | RRMS (60) | McDonald 2001 &Poser | Not reported | Mean  Group 1:29.5  (±SD 3.8)  Group 2: 35.7 (±SD 10.4) | Pregnant (n=42) & Not pregnant | Mean EDSS 1.6 (±SD 1.3) | Pregnancy  [MS relapses, Mechanism] | Prospective | Case Control | No |
| *Sargin*[*^286^*](#_ENREF_286)  *(2017)* | Asia | Medical records review | 5 | MS subtype unspecified (5) | Clinician reported | Not reported | Not reported | Pregnant (n=5) | Not reported | Pregnancy  [Pregnancy complicati-ons, Anesthesia] Fetal/  Neonatal Outcomes | Retrospective | Cohort | Yes |
| *Schneider*[*^287^*](#_ENREF_287)  *(2012)* | Europe: *Germany* | Primary data collection | 2 | RRMS (2) | Not reported | Not reported | Not reported | Pregnant (n=2) | EDSS Range  2.5-9.5 | Pregnancy  [DMT exposure], Fetal/  Neonatal Outcomes | Prospective | Case Series | No |
| *Sena*[*^288^*](#_ENREF_288)  *(2012)* | Europe: *Portugal* | Primary data collection | 132 | RRMS (132) | McDonald 2005 | Not reported | Mean  Group 1: 37.3  (±SD 10.2)  Group 2: 38.6  (±SD 6.8)  Group 3: 32.0  (±SD 6.6) | Not pregnant | Mean EDSS  Group 1: 2.55  (±SD 1.30) Group 2: 2.31  (±SD 1.34)  Group 3: 1.60  (±SD 1.03) | Birth Control | Retrospective | Cross-section-al | No |
| *Sepulveda*[*^289^*](#_ENREF_289)  *(2020)* | Europe: *Spain* | Medical records review | 7 | RRMS (7) | McDonald 2010 | Not reported | Age Range  19-33 | Pregnant (n=10) & Not pregnant | EDSS Range 1.5-5.5 | Pregnancy  [MS relapses, Disability outcomes, DMT exposure] | Retrospective | Case Series | No |
| *Sepulveda*[*^290^*](#_ENREF_290)  *(2016)* | Europe: *Spain* | Primary data collection | 25 | RRMS (25) | Not reported | Not reported | Mean 34.8 | Not pregnant | EDSS Range  1-4 | Fertility | Prospective | Case Control& Cross-section-al | No |
| *Shabas*[*^291^*](#_ENREF_291)  *(2000)* | North America: *United States* | Primary data collection | 220 | MS subtype unspecified (22) | Diagnosis per neurologi-st | Not reported | Age Range  23-81 | Not pregnant | Ambulation status: 47% ambulatory, 38% with walking assistive device, 15% non-ambulatory | Menopause  [Symptoms], Cancer/  Cancer screening, Services around family planning/  pregnancy/  post-pregnancy,Menstruati-on | Retrospective | Cross-section-al | Yes |
| *Shimizu*[*^292^*](#_ENREF_292)  *(2015)* | Asia: *Japan* | Primary data collection | 20 | MS subtype unspecified (20) | Not reported | Not reported | Age Range  25-43 | Pregnant  (n=20) | Not reported | Pregnancy  [DMT exposure] | Prospective | Cohort | No |
| *Sicotte*[*^293^*](#_ENREF_293)  *(2002)* | North America: *United States* | Primary data collection | 12 | RRMS (6), SPMS (6) | Not reported | Not reported | Mean 44  (Range 28-50) | Not pregnant | EDSS Range  1-6.5 | Sex hormones & MS | Prospective | Clinical trial | No |
| *Singh*[*^294^*](#_ENREF_294)  *(2015)* | Europe *Netherla-nds* | Primary data collection | 31 | MS subtype unspecified (31) | Not reported | Not reported | Mean 32  (±SD 4) | Pregnant  (n=31) | Not reported | Pregnancy  [MS relapses, Mechanism] | Prospective | Cohort | No |
| *Smeltzer*[*^295^*](#_ENREF_295)  *(2002)* | Not reported | Primary data collection | 15 | RRMS (11), MS other subtype (4) | Not reported | White | Mean 30.3  (Range 24-40) | Pregnant (n=22) *&* Not pregnant | All ambulatory without assistive devices or ambulatory with occasional use of assistive device | Pregnancy, Motherhood/parenthood | Prospective | Cross-section-al | No |
| *Smith*[*^296^*](#_ENREF_296)  *(2019)* | North America: *United States* | Medical records review | 43 | MS subtype unspecified (43) | Not reported | White, African, Black, or African-American, Hispanic, Latino, Central or South American | Mean  Group 1: 29.48  (±SD 5.55)  Group 2: 33.04  (±SD 4.33) | Pregnant  (n=45) | Median EDSS Group 1: 2.25  (IQR 1-2.5) Group 2: 2 (IQR 0.5-2.5) | Pregnancy  [MS relapses, Disability outcomes, MRI outcomes, DMT exposure], Services around family planning/  pregnancy/  post-pregnancy | Retrospective | Cohort | No |
| *Smith*[*^297^*](#_ENREF_297)  *(2020)* | North America: *United States* | Medical records review | 55 | RIS (1), RRMS (54) | McDonald 2017 | White, Hispanic, Latino, Central or South American, African, Black, or African-American, Asian | Age Range  28-34.5 | Pregnant (n=74) | EDSS Range 1-3 | Pregnancy  [Pregnancy complicati-ons, MS relapses, DMT exposure], Fetal/  Neonatal Outcomes | Prospective | Cohort | No |
| *Smith*[*^298^*](#_ENREF_298)  *(1992)* | Europe: *United Kingdom* | Primary data collection | 30 | MS subtype unspecified (30) | Women with MS attending a conferen-ce | Not reported | Mean  Group 1: 56.1  Group 2: 35.8 | Not pregnant | Patient ranked 0-3 (no disability-severe disability) across six areas (strength, speech, sensation, bladder/  bowel, sight, and coordination)  Mean total score of 7.5  (±SD 3.5) | Menopause & Menstruati-on | Retrospective | Cross-section-al | Yes |
| *Snarski*[*^299^*](#_ENREF_299)  *(2015)* | South America: *Brazil,* Europe | Clinical registry | 7 | MS subtype unspecified (7) | Not reported | Not reported | Age Range  31-39 | Pregnant (n=11) | Not reported | Pregnancy  [Pregnancy complicati-ons, DMT exposure], Fertility | Retrospective | Cohort | No |
| *Solmaz*[*^300^*](#_ENREF_300)  *(2018)* | Asia: *Turkey* | Primary data collection | 42 | RRMS (34), SPMS (7), PPMS (1) | McDonald 2010 | Not reported | Mean 41.9 | Not pregnant | EDSS Range  0-7 | Sexual dysfunction | Prospective | Case Control | Yes |
| *Spadaro*[*^301^*](#_ENREF_301)  *(2019)* | Europe: *Italy* | Primary data collection | 21 | RRMS (21) | McDonald 2010 | Not reported | Median  Group 1: 28.5  (IQR 22.5-35.3)  Group 2: 34  (IQR 31-36)  Group 3: 33  (IQR 30.5-35.5)  Group 4: 33.5  (IQR 30.25-36.75)  Group 5: 33.5  (IQR 23.25-36.5) | Pregnant  (n=11) | Median EDSS Group 1: 0.5 (IQR 0-1.75) Group 2: 1 (IQR 0.25-1.37)  Group 3: 1 (IQR 0-1.75) Group 4: 1 (IQR 0-1.25) Group 5: 1 (IQR 0.25-3.62)  Group 6: 1 (IQR 1-1.25) | Pregnancy  [Mechanism] | Prospective | Cohort | No |
| *Steck*[*^302^*](#_ENREF_302)  *(2007)* | Europe: *Germany, Greece, Switzerla-nd* | Primary data collection | 103 | MS subtype unspecified (103) | Not reported | Not reported | Mean 40.8  (±SD 5.7) | Not pregnant | Not reported | Child health/  Developme-ntal outcomes | Retrospective | Cross-section-al | Yes |
| *Stenager*[*^303^*](#_ENREF_303)  *(1994)* | Europe: *Denmark* | Primary data collection | 29 | RRMS (6), SPMS/ PPMS (17), MS subtype other (6) | Poser | Not reported | Mean 38.4  (±SD 5.4,  Range 25-45) | Pregnant (n=22 parous women) & Not Pregnant | Kurtzke Disability Status Scale  Mean 3.6 (±SD 2.0) | Pregnancy  [Disability outcomes] | Prospective | Cohort | No |
| *Stepleman*[*^304^*](#_ENREF_304)  *(2016)* | North America: *United States* | Primary data collection | 64 | RRMS, SPMS, PPMS  (not stratified by gender) | Documen-ted definitive diagnosis of MS | White, African, Black, or African-American , Hispanic, Latino, Central or South American, Other | Mean 43.53  (±SD 10.24,  Range 20-65) | Not pregnant | PDDS Range normal- confined to a wheelchair | Sexual dysfunction | Retrospective | Cross-section-al | Yes |
| *Stuifbergen*[*^305^*](#_ENREF_305)  *(1999)* | Not reported | Primary data collection | 9 | MS subtype unspecified (9) | Not reported | Not reported | Not reported | Not pregnant | Not reported | Services around family planning/  pregnancy/  post-pregnancy | Prospective | Clinical trial | Yes |
| *Tepavcevic*[*^306^*](#_ENREF_306)  *(2008)* | Europe: *Serbia* | Primary data collection | 78 | RRMS (54), SPMS (22), PPMS (2) | McDonald 2001 | Not reported | Mean 41.7  (±SD 9.3) | Not pregnant | Mean EDSS 4.6 (±SD 1.6) | Sexual dysfunction | Prospective | Cross-section-al | Yes |
| *Teter*[*^307^*](#_ENREF_307)  *(2014)* | North America: *United States* | Clinical registry | 1523 | RRMS (1206), SPMS (216), PPMS/  PRMS (99) | Not reported | White, African, Black, or African-American, Other | Mean 54.3  (Range 45-82) | Pregnant (n=1195 parous women) & Not pregnant | Mean EDSS 3.5 | Pregnancy  [Disability outcomes | Retrospective | Cohort | No |
| *Thiel*[*^308^*](#_ENREF_308)  *(2016)* | Europe: *Germany* | Clinical registry | 445 | RRMS (445) | Not reported | Not reported | Mean  Group 1: 31.54  Group 2: 32.20 | Pregnant (n=445) | Not reported | Pregnancy  [Pregnancy complicati-ons, DMT exposure],  Fetal/  Neonatal Outcomes | Prospective | Cohort | No |
| *Thompson*[*^309^*](#_ENREF_309)  *(1986)* | North America: *United States* | Medical records review | 178 | MS subtype unspecified (178) | Schumacher | Not reported | Not reported | Pregnant (n=130 women with pregnancies)& Not pregnant | Mean EDSS  Group 1: 3.35  Group 2: 4.37  Group 3:  3.90 | Pregnancy  [Disability outcomes] | Retrospective | Cohort | No |
| *Thone*[*^310^*](#_ENREF_310)  *(2016)* | Europe: *Germany* | Primary data collection | 85 | RRMS (85) | McDonald 2010 | White | Mean 28.8  (Range 18-35) | Not pregnant | Mean EDSS 2.8 (±SD 1.6) | Fertility | Prospective | Case Control | No |
| *Thone*[*^311^*](#_ENREF_311)  *(2013)* | Europe *Germany* | Primary data collection | 76 | RRMS (76) | McDonald 2005 | White | Mean 29.1  (±SD 4.4) | Not pregnant | Median EDSS 2.0 (range  1.0-7.0) | Fertility Sex hormones & MS | Prospective & Retrospective | Case Control | No |
| *Tong*[*^312^*](#_ENREF_312)  *(2018)* | Asia: *China* | Medical records review | 170 | MS subtype unspecified (170) | McDonald 2010 | Not reported | Mean 34.8  (Range 18-67) | Pregnant (n=239) & Not pregnant | Not reported | Pregnancy  [MS relapses] | Retrospective | Cohort | No |
| *Trenova*[*^313^*](#_ENREF_313)  *(2013)* | Europe:  *Bulgaria* | Primary data collection | 35 | RRMS (35) | McDonald (year not specified) | Not reported | Mean 34.8  (Range 19-49) | Not pregnant | EDSS Range Group 1:  2-8.5  Group 2:  1-5.5  & Scripps Neurological Rating Scale  Group 1: 37-94  Group 2:  51-99 | Sex hormones & MS | Prospective | Cohort | No |
| *Triantafyllou*[*^314^*](#_ENREF_314)  *(2016)* | Europe | Primary data collection | 81 | RRMS (81) | McDonald 2001 | Not reported | Mean  Group 1: 36.06  (±SD 9.60, range 18-54)  Group 2: 51.93 (±SD 6.15, range 42-64) | Not pregnant | EDSS Range  Group 1:  0-5.0  Group 2:  0-6.0 | Sex hormones & MS | Prospective | Cross-section-al | No |
| *Triplett*[*^315^*](#_ENREF_315)  *(2020)* | Australia/New Zealand | Medical records review | 13 | MS subtype unspecified (13) | Not reported | Not reported | Median 34  (Range 26-40)  *Combined for all genders or sexes | Pregnant (n=15) | Not reported | Pregnancy  [MS relapses , DMT exposure], Fetal/  Neonatal Outcomes | Retrospective | Cohort | No |
| *Tudor*[*^316^*](#_ENREF_316)  *(2018)* | Europe: *United Kingdom* | Primary data collection | 54 | RRMS, SPMS, PPMS, MS other subtype  (npt stratified by gender) | Not reported | Not reported | Mean 42.4  (Range 21-64)  *Combined for all genders or sexes | Not pregnant | Not reported | Sexual dysfunction | Prospective | Cross-section-al | Yes |
| *Tuohy*[*^317^*](#_ENREF_317)  *(2015)* | Europe | Primary data collection | 61 | RRMS (61) | Not reported | Not reported | Mean 33 | Pregnant (n=15) & Not pregnant | Highest EDSS 5.5 | Pregnancy  [Pregnancy complicati-ons, DMT exposure] Fertility, Fetal/  Neonatal Outcomes | Prospective | Cohort | No |
| *Turk*[*^318^*](#_ENREF_318)  *(2018)* | Asia: *Turkey* | Primary data collection | 86 | RRMS (86) | McDonald 2010 | Not reported | Mean  Group 1: 44.8  (±SD 6.4)  Group 2: 45.5  (±SD 6.4) | Not pregnant | Mean EDSS 3.9 (±SD 1.7) | Menopause | Prospective | Cohort | No |
| *Twork*[*^319^*](#_ENREF_319)  *(2007)* | Europe | Primary data collection | 1,089 | RRMS (631), SPMS (382), PPMS (76) | Self-reported | Not reported | Mean 40.8 | Not pregnant | EDSS Range  0-9.5 | Motherhood/parenthood | Prospective | Cross-section-al | Yes |
| *Tzortzis*[*^320^*](#_ENREF_320)  *(2008)* | Europe *Greece* | Primary data collection | 63 | RRMS (58), PPMS (5) | McDonald 2001 | White | Mean 33  (±SD 6.4,  range 19-51) | Not pregnant | Mean EDSS 2.3  (range 0-3.5) | Sexual dysfunction | Prospective | Cohort | Yes |
| *Valiani*[*^321^*](#_ENREF_321)  *(2018)* | Asia: *Iran* | Primary data collection | 110 | RRMS (90), SPMS (7), PPMS (9),  PRMS (4) | Not reported | Not reported | Mean 32.4 | Pregnant (n=110) | Not reported | Pregnancy  [Pregnancy complicati-ons, MS relapses], Fetal/  Neonatal Outcomes | Retrospective | Cross-section-al | No |
| *Valleroy*[*^322^*](#_ENREF_322)  *(1984)* | North America: *United States* | Primary data collection | 149 | MS subtype unspecified (149) | Not reported | Not reported | Mean 34  (range 17-69) | Not pregnant | Not reported | Sexual dysfunction | Prospective | Cross-section-al | Yes |
| *van der Kop*[*^323^*](#_ENREF_323)  *(2011)* | North America: *Canada* | Clinical registry | 321 | RRMS (299), SPMS (18), PPMS (4) | McDonald 2005 & Poser | Not reported | Age Range  19-44 | Pregnant (n=432) | EDSS Range  0-7.5 | Pregnancy  [Pregnancy complicati-ons],  Fetal/  Neonatal Outcomes | Retrospective | Cohort | No |
| *Vanya*[*^324^*](#_ENREF_324)  *(2014)* | Europe: *Hungary* | Medical records review | 130 | RRMS (130) | McDonald 2001 | Not reported | Mean  Group 1:26.37  (±SD 6.34)  Group 2: 27.72  (±SD 3.9) | Pregnant  (n=65) | Mean EDSS  Group 1: 1.36  (±SD 1.2)  Group 2: 1.48  (±SD 4.4) | Pregnancy  [Pregnancy complicati-ons], Fetal/  Neonatal Outcomes | Prospective | Case Control | No |
| *Verdier-Taillefer*[*^325^*](#_ENREF_325)  *(1990)* | Europe *France* | Primary data collection | 5296 | MS subtype unspecified (5296) | Not reported | Not reported | Mean 46.9 | Not pregnant | Not reported | Fertility | Retrospective | Cross-section-al | No |
| *Verdru*[*^326^*](#_ENREF_326)  *(1994)* | Europe: *Belguim* | Medical records review | 200 | MS subtype unspecified (200) | Not reported | Not reported | Not reported | Pregnant (n=146 women who had pregnancies) &  Not pregnant | Wheelchair dependent | Pregnancy  [Disability outcomes] | Retrospective | Case Control | No |
| *Villaverde-Gonzalez*[*^327^*](#_ENREF_327)  *(2020)* | Europe: *Spain* | Clinical registry | 27 | RRMS (27) | McDonald 2005 | Not reported | Mean 31.56  (±SD 4.1, range 23-41) | Pregnant (n=21) | Mean EDSS  Group 1: 1.29  (±SD 1.1)  Group 2: 1.35  (±SD 1.15) | Pregnancy  [Pregnancy complicati-ons, MS relapses, Disability outcomes, MRI outcomes, DMT exposure], Fetal/  Neonatal Outcomes | Retrospective | Cohort | No |
| *Voskuhl*[*^328^*](#_ENREF_328)  *(2016)* | North America: *United States* | Primary data collection | 158 | RRMS (158) | McDonald 2010 | White, African, Black, or African-American , Hispanic, Latino, Central or South American, Other | Mean  Group 1: 37.7 (±SD 7.6) Group 2: 37.1 (±SD 7.3) | Not pregnant | EDSS Range  0-5.5 | Sex hormones & MS | Prospective | Clinical trial | Yes |
| *Vukusic*[*^329^*](#_ENREF_329)  *(2020)* | Not reported | Primary data collection | 222 | RRMS (222) | Not reported | Not reported | Mean  Group 1: 30.4  Group 2: 32.5 | Pregnant (n=62) | Not reported | Pregnancy  [Pregnancy complicati-ons, DMT exposure], Fetal/  Neonatal Outcomes | Prospective & Retrospective | Clinical trial | No |
| *Vukusic*[*^330^*](#_ENREF_330)  *(2004)* | Europe | Primary data collection | 227 | RRMS (277) | Poser | Not reported | Mean 30 | Pregnant (n=227) | Mean DSS 1.3 | Pregnancy  [MS relapses, Disability outcomes, Anesthesia] | Prospective | Cohort | No |
| *Vukusic*[*^331^*](#_ENREF_331)  *(2015)* | Europe: *France* | Clinical registry | 6 | MS subtype unspecified (6) | Not reported | Not reported | Mean 31.4 | Pregnant (n=6) | Mean EDSS 4.3 | Pregnancy  [MS relapses, DMT exposure] | Retrospective | Case Series | No |
| *Webb*[*^332^*](#_ENREF_332)  *(2020)* | Europe: *United Kingdom* | Primary data collection | 60 | RRMS (55), SPMS (1), PPMS (2),  MS subtype not unspecified (2) | MS registry | Not reported | Mean 34.4 | Not pregnant | Not reported | Pregnancy  [MS relapses, DMT exposure], Fertility | Prospective | Cross-section-al | No |
| *Weber-Schoendorfer*[*^333^*](#_ENREF_333)  *(2009)* | Europe: *Germany* | Primary data collection | 100 | MS subtype unspecified (100) | Not reported | Not reported | Median  Group 1: 31  Group 2: 30  Group 3: 31  (Overall range 18-44) | Pregnant  (n=164) | Not reported | Pregnancy  [Pregnancy complicati-ons, DMT exposure] | Prospective | Cohort | No |
| *Wei*[*^334^*](#_ENREF_334)  *(1997)* | Europe: *United Kingdom* | Primary data collection | 26 | RRMS (14), SPMS (9), PPMS (3) | Poser | Not reported | Mean  Group 1: 43  Group 2: 49  Group 3: 49  (Overall range 25-70) | Not pregnant | EDSS Range  1-9 | Sex hormones & MS | Prospective | Cross-section-al | No |
| *Weinshenker*[*^335^*](#_ENREF_335)  *(1989)* | North America: *Canada* | Primary data collection | 231 | Not reported (231) | Poser | Not reported | Mean  Group 1: 27  (±SD 10)  Group 2: 27  (±SD 8)  Group 3: 30  (±SD 9)  Group 4: 34  (±SD 10) | Pregnant (n=195) | Mean EDSS  4.86  (±SD 2.84, range 0-9.5) | Pregnancy  [Disability outcomes] | Retrospective | Cross-section-al | No |
| *Winder*[*^336^*](#_ENREF_336)  *(2016)* | Europe: *Germany* | Primary data collection | 44 | RRMS/ SPMS (44) | McDonald 2010 | Not reported | Mean 36.5  (±SD 9.9) | Not pregnant | Median EDSS 3.5 (IQR 2-6) | Sexual dysfunction | Prospective | Cross-section-al | Yes |
| *Wingerchuk*[*^337^*](#_ENREF_337)  *(2006)* | North America: *United States* | Primary data collection | 3 | RRMS (3) | Not reported | Not reported | Age Range  29-36 | Not pregnant | EDSS Range  1.0-2.5 | Menstruati-on | Prospective | Case Series | Yes |
| *Winkelmann*[*^338^*](#_ENREF_338)  *(2018)* | Europe: *Germany* | Primary data collection & Medical records review | 90 | RRMS (90) | McDonald 2005 | Not reported | Mean  Group 1: 29  (±SD 4.8)  Group 2: 28  (±SD 4.9)  Group 3: 27  (±SD 4.9) | Pregnant (n=70) | Mean EDSS Group 1: 1.3 (±SD 1.3) Group 2: 1.3 (±SD 1.3)  Group 3: 1.2 (±SD 1.0) | Pregnancy  [MS relapses, Disability outcomes, DMT exposure] | Prospective | Case Control | No |
| *Worthington*[*^339^*](#_ENREF_339)  *(1994)* | Europe: *United Kingdom* | Primary data collection | 37 | RRMS (27), SPMS (9), PPMS (1) | Poser | Not reported | Mean  Group 1: 30.0  (range 25-37)  Group 2: 32.5  (range 24-39) | Pregnant (n=15) & Not pregnant | EDSS Range  Group 1:  3.5-6.0  Group 2:  3.5-6.0 | Pregnancy  [MS relapses, Disability outcomes], Fetal/  Neonatal Outcomes | Prospective | Cohort | No |
| *Wu*[*^340^*](#_ENREF_340)  *(2017)* | Asia: *China* | Primary data collection | 7 | CIS (2), MS subtype unspecified (5) | Diagnostic criteria revised by consorti-um of MS centers 2016 | Not reported | Age Range  20-35 | Pregnant (n=7) | EDSS Range  3.0-5.0 | Pregnancy  [MS relapses, Disability outcomes] | Retrospective | Cohort | No |
| *Yalcin*[*^341^*](#_ENREF_341)  *(2017)* | Asia: *Turkey* | Medical records review | 43 | MS subtype unspecified (43) | *ICD-10 code of MS* & McDonald 2010 | Not reported | Mean 30.2 | Pregnant (n=43)* | Mean EDSS 0.7 | Pregnancy  [Pregnancy complicati-ons, MS relapses], Fetal/  Neonatal Outcomes | Retrospective | Case Control | No |
| *Young*[*^342^*](#_ENREF_342)  *(2016)* | Europe: *United Kingdom* | Primary data collection | 519 | RRMS (458), SPMS (161), PPMS (71), MS other subtype (32) | Not reported | Not reported | Mean 48.9  (±SD 11.6,  Range 17-82) | Not prgnant | EDSS Range  0-9 | Sexual dysfunction | Retrospective | Cross-section-al | Yes |
| *Zafarmand*[*^343^*](#_ENREF_343)  *(2019)* | Asia: *Iran* | Primary data collection | 15 | MS subtype unspecified (15) | Not reported | Not reported | Mean 29.2 | Pregnant  (n=15) | Not reported | Pregnancy  [Pregnancy complications], Fetal/  Neonatal Outcomes | Prospective | Cross-section-al | No |
| *Zaherian*[*^344^*](#_ENREF_344)  *(2020)* | Asia: *Iran* | Primary data collection | 45 | MS subtype unspecified (45) | Physician diagnosis | Not reported | Mean 35.7 | Not pregnant | Not reported | Sexual dysfunction | Prospective | Clinical trial | Yes |
| *Zakrzewska-Pniewska*[*^345^*](#_ENREF_345)  *(2011)* | Europe *Poland* | Primary data collection | 46 | RRMS (27),  SPMS (12),  MS other subtype (7) | McDonald 2001 | Not reported | Mean 39.28  (range 19-65) | Not pregnant | EDSS Range  1.0-7.0 | Sex hormones & MS | Prospective | Cohort | No |
| *Zanghi*[*^346^*](#_ENREF_346)  *(2020)* | Europe: *Italy* | Clinical registry | 74 | RRMS (74) | Poser & McDonald 2010 | Not reported | Mean 36.2  (±SD 5.9) | Pregnant (n=81) | Median EDSS 2.0  (IQR 1.0-3.5,  Range 1-3.5) | Pregnancy  [MS relapses, MRI outcomes, DMT exposure] | Prospective | Cross-section-al | No |
| *Zavoreo*[*^347^*](#_ENREF_347)  *(2016)* | Europe: *Croatia* | Primary data collection | 56 | RRMS (56) | McDonald criteria | Not reported | Mean 35  (range 23-47)  *Combined for all genders or sexes | Not pregnant | EDSS Range  2-4  *Combined for all genders or sexes | Sexual dysfunction | Prospective | Cohort | Yes |
| *Zengin*[*^348^*](#_ENREF_348)  *(2020)* | Asia: *Turkey* | Primary data collection | 12 | MS subtype unspecified (12) | Not reported | Not reported | Mean 28.03  (range 23-36) | Pregnant  (n=12) | EDSS Range  1-3.5 | Breastfeedi-ng [non-DMT drug exposure] | Prospective | Cohort | No |
| *Zivadinov*[*^349^*](#_ENREF_349)  *(1999)* | Europe: *Italy* | Primary data collection | 70 | RRMS (50), SPMS (4) PPMS (16) | Poser | Not reported | Not reported | Not pregnant | EDSS (score unspecified) | Sexual dysfunction | Prospective | Cross-section-al | Yes |
| *Zivadinov*[*^350^*](#_ENREF_350)  *(2003)* | Europe: *Italy* | Primary data collection | 15 | RRMS (15) | McDonald 2001 | Not reported | Mean  Group 1: 30.7  Group 2: 47.7  *Combined for all genders or sexes | Not pregnant | EDSS  Group 1:  Mean 1.4  Median 1  Group 2:  Mean: 3.7  Median: 2.8  *Combined for all genders or sexes | Sexual dysfunction | Prospective | Cross-section-al | Yes |
| *Zorgdrager*[*^351^*](#_ENREF_351)  *(1997)* | Europe: *Netherla-nds* | Primary data collection | 85 | RRMS/ SPMS (60), PPMS (12) | Poser | Not reported | Mean  Group 1: 39  Group 2: 36  (overall range 20-50) | Not pregnant | Not reported | Menstruati-on | Retrospective | Cross-section-al | No |
| *Zorgdrager*[*^352^*](#_ENREF_352)  *(2002)* | Europe: *Netherla-nds* | Clinical registry & Primary data collection | 53 | RRMS (32), SPMS (21) | Not reported | Not reported | Mean  Group 1: 35  Group 2: 36 | Not pregnant | Not reported | Menstruati-on | Retrospective | Cohort | No |
| *Zorzon*[*^353^*](#_ENREF_353)  *(1999)* | Europe: *Italy* | Primary data collection | 70 | RRMS (50), SPMS (16), PPMS (4) | Poser | Not reported | Mean 40.2 | Not pregnant | Mean EDSS: 2.6; Median EDSS: 2.0 | Sexual dysfunction | Prospective | Case Control | Yes |
| *Zuluaga*[*^354^*](#_ENREF_354)  *(2019)* | Europe: *Spain* | Primary data collection | 501 | CIS (501) | McDonald 2010 & Poser | Not reported | Mean 31.1  (±SD 7.8) | Pregnant (n=302 parous women) & Not pregnant | EDSS 3.0  N= 67  EDSS 6.0  N= 13 | Pregnancy, Breastfeeding  [MS relapses , disability outcomes, MRI outcomes], Menarche/  puberty | Prospective & Retrospective | Cohort | No |

*Spanish **Turkish

**References**

1. Achiron A, Ben-David A, Gurevich M, et al. Parity and disability progression in relapsing-remitting multiple sclerosis. Journal of Neurology 2020;251:1133-1137.

2. Achiron A, Rotstein Z, Noy S, Mashiach S, Dulitzky M, Achiron R. Intravenous immunoglobulin treatment in the prevention of childbirth-associated acute exacerbations in multiple sclerosis: a pilot study. J Neurol 1996;243:25-28.

3. Achiron A, Kishner I, Dolev M, et al. Effect of intravenous immunoglobulin treatment on pregnancy and postpartum-related relapses in multiple sclerosis. J Neurol 2004;251:1133-1137.

4. Jalkanen A, Alanen A, Airas L. Pregnancy outcome in women with multiple sclerosis: results from a prospective nationwide study in Finland. Multiple Sclerosis Journal 2010;16:950-955.

5. Airas L, Jalkanen A, Alanen A, Pirttila T, Marttila RJ. Breast-feeding, postpartum and prepregnancy disease activity in multiple sclerosis. Neurology 2010;75:474-476.

6. Airas L, Saraste M, Rinta S, et al. Immunoregulatory factors in multiple sclerosis patients during and after pregnancy: relevance of natural killer cells. Clinical and experimental immunology 2007;151:235-243.

7. Airas L, Nikula T, Huang Y-H, Lahesmaa R, Wiendl H. Postpartum-activation of multiple sclerosis is associated with down-regulation of tolerogenic HLA-G. Journal of Neuroimmunology 2007;187:205-211.

8. Al-Shammri S, Rawoot P, Azizieh F, et al. Th1/Th2 cytokine patterns and clinical profiles during and after pregnancy in women with multiple sclerosis. J Neurol Sci 2004;222:21-27.

9. Alanazy MH, Asiri A, Edrees MF, Abuzinadah AR. Impact of neurological diseases on family planning: A single-center experience. Medicine (Baltimore) 2020;99:e22978-e22978.

10. Alehashemi A, Mostafavian Z, Dareini N. Sexual function in iranian female multiple sclerosis patients. Open Access Macedonian Journal of Medical Sciences 2019;7:1303-1308.

11. Alexander M, Bashir K, Alexander C, Marson L, Rosen R. Randomized Trial of Clitoral Vacuum Suction Versus Vibratory Stimulation in Neurogenic Female Orgasmic Dysfunction. Archives of Physical Medicine & Rehabilitation 2018;99:299-305.

12. Alping P, Askling J, Burman J, et al. Cancer Risk for Fingolimod, Natalizumab, and Rituximab in Multiple Sclerosis Patients. Ann Neurol 2020;87:688-699.

13. Alroughani R, Akhtar S, Zeineddine M, et al. Risk of relapses during pregnancy among multiple sclerosis patients. Mult Scler Relat Disord 2019;34:9-13.

14. Alroughani R, Alowayesh MS, Ahmed SF, Behbehani R, Al-Hashel J. Relapse occurrence in women with multiple sclerosis during pregnancy in the new treatment era. Neurology 2018.

15. Altintas A, Najar B, Gozubatik-Celik G, Menku Sukriye F. Pregnancy Data in a Turkish Multiple Sclerosis Population. European neurology 2015;74:296-302.

16. Alwan S, Yee I, Dybalski M, et al. Reproductive decision making after the diagnosis of multiple sclerosis (MS). Multiple Sclerosis Journal 2013;19:351-358.

17. Alwan S, Dybalski M, Yee IM, et al. Multiple sclerosis and pregnancy: a comparison study. The Canadian journal of neurological sciences Le journal canadien des sciences neurologiques 2013;40:590-596.

18. Amato MP, Portaccio E, Ghezzi A, et al. Pregnancy and fetal outcomes after interferon-beta exposure in multiple sclerosis. Neurology 2010;75:1794-1802.

19. Andersen JB, Moberg JY, Niclasen J, Laursen B, Magyari M. Mental health among children of mothers with multiple sclerosis: A Danish cohort and register-based study. Brain Behav 2018;8:e01098.

20. Andersen JB, Moberg JY, Spelman T, Magyari M. Pregnancy Outcomes in Men and Women Treated With Teriflunomide. A Population-Based Nationwide Danish Register Study. Frontiers in Immunology 2018;9:2706-2706.

21. Araz Altay M, Gorker I, Guler S, Demirci Sipka B, Atas T. Evaluation of psychiatric characteristics in children of parents with multiple sclerosis. Anadolu Psikiyatri Dergisi 2020;21:515-522.

22. Azari-Barzandig R, Sattarzadeh-Jahdi N, Mehrabi E, Nourizadeh R, Najmi L. Sexual dysfunction in Iranian Azeri women with multiple sclerosis: Levels and correlates. Crescent Journal of Medical and Biological Sciences 2019;6:381-387.

23. Bader AM, Hunt CO, Datta S, Naulty JS, Ostheimer GW. Anesthesia for the obstetric patient with multiple sclerosis. Journal of clinical anesthesia 1988;1:21-24.

24. Bansil S, Lee HJ, Jindal S, Holtz CR, Cook SD. Correlation between sex hormones and magnetic resonance imaging lesions in multiple sclerosis. Acta Neurologica Scandinavica 1999;99:91-94.

25. Baroncini D, Annovazzi PO, De Rossi N, et al. Impact of natural menopause on multiple sclerosis: a multicentre study. Journal of Neurology, Neurosurgery & Psychiatry 2019;90:1201-1206.

26. Bartnik P, Wielgos A, Kacperczyk J, et al. Sexual dysfunction in female patients with relapsing-remitting multiple sclerosis. Brain Behav 2017;7:e00699.

27. Bartnik P, Wielgos A, Kacperczyk-Bartnik J, et al. Evaluation of reproductive health in female patients with multiple sclerosis in Polish population. Journal of Clinical Neuroscience 2018;53:117-121.

28. Becker H, Stuifbergen A, Tinkle M. Reproductive health care experiences of women with physical disabilities: a qualitative study. Archives of Physical Medicine and Rehabilitation 1997;78:S26-33.

29. Benoit A, Durand-Dubief F, Amato M-P, et al. History of multiple sclerosis in 2 successive pregnancies: A French and Italian cohort. Neurology 2016;87:1360-1367.

30. Berenguer-Ruiz L, Gimenez-Martinez J, Palazon-Bru A, Sempere AP. Relapses and obstetric outcomes in women with multiple sclerosis planning pregnancy. J Neurol 2019;266:2512-2517.

31. Bernardi S, Grasso MG, Bertollini R, Orzi F, Fieschi C. The influence of pregnancy on relapses in multiple sclerosis: a cohort study. Acta Neurologica Scandinavica 1991;84:403-406.

32. Birk K, Ford C, Smeltzer S, Ryan D, Miller R, Rudick RA. The clinical course of multiple sclerosis during pregnancy and the puerperium. Arch Neurol 1990;47:738-742.

33. Blackmore DE, Hart SL, Albiani JJ, Mohr DC. Improvements in Partner Support Predict Sexual Satisfaction Among Individuals With Multiple Sclerosis. Rehabilitation Psychology 2011;56:117-122.

34. Borello-France D, Leng W, O'Leary M, et al. Bladder and sexual function among women with multiple sclerosis. Mult Scler 2004;10:455-461.

35. Boskovic R, Wide R, Wolpin J, Bauer DJ, Koren G. The reproductive effects of beta interferon therapy in pregnancy: A longitudinal cohort. Neurology 2005;65:807-811.

36. Bove R, Healy BC, Secor E, et al. Patients report worse MS symptoms after menopause: findings from an online cohort. Mult Scler Relat Disord 2015;4:18-24.

37. Bove R, Rankin K, Chua AS, et al. Oral contraceptives and MS disease activity in a contemporary real-world cohort. Mult Scler 2018;24:227-230.

38. Bove R, Rankin K, Lin C, et al. Effect of assisted reproductive technology on multiple sclerosis relapses: Case series and meta-analysis. Multiple Sclerosis Journal 2020;26:1410-1419.

39. Bove R, Vaughan T, Chitnis T, Wicks P, De Jager PL. Women's experiences of menopause in an online MS cohort: A case series. Mult Scler Relat Disord 2016;9:56-59.

40. Bove R, Healy Brian C, Musallam A, Glanz Bonnie I, De Jager Philip L, Chitnis T. Exploration of changes in disability after menopause in a longitudinal multiple sclerosis cohort. Multiple sclerosis (Houndmills, Basingstoke, England) 2016;22:935-943.

41. Bove R, White Charles C, Fitzgerald Kathryn C, et al. Hormone therapy use and physical quality of life in postmenopausal women with multiple sclerosis. Neurology 2016;87:1457-1463.

42. Boz C, Terzi M, Zengin Karahan S, Sen S, Sarac Y, Emrah Mavis M. Safety of IV pulse methylprednisolone therapy during breastfeeding in patients with multiple sclerosis. Multiple sclerosis (Houndmills, Basingstoke, England) 2018;24:1205-1211.

43. Brandt-Wouters E, Gerlach Oliver HH, Hupperts Raymond MM. The effect of postpartum intravenous immunoglobulins on the relapse rate among patients with multiple sclerosis. International journal of gynaecology and obstetrics: the official organ of the International Federation of Gynaecology and Obstetrics 2016;134:194-196.

44. Bsteh G, Algrang L, Hegen H, et al. Pregnancy and multiple sclerosis in the DMT era: Insights from a cohort study in Western Austria. Multiple Sclerosis Journal 2018;24:1011-1012.

45. Burkill S, Vattulainen P, Geissbuehler Y, et al. The association between exposure to interferon-beta during pregnancy and birth measurements in offspring of women with multiple sclerosis. PLoS One 2019;14:e0227120.

46. Cardenas-Robledo S, Lopez L, Acosta-Camargo L. Contraceptive use frequency among women with multiple sclerosis. Neurologia Argentina 2019;11:130-135.

47. Carnero C, Pettinicchi JP, Caride A, Lopez PA. Sexual Dysfunction in Patients with Multiple Sclerosis from Argentina: What are the Differences Between Women and Men? Sexuality and Disability 2019;37:521-539.

48. Carvalho AT, Veiga A, Morgado J, et al. Multiple sclerosis and motherhood choice: an observational study in Portuguese women patients. Rev Neurol 2014;59:537-542.

49. Celik DB, Poyraz EC, Bingol A, Idiman E, Ozakbas S, Kaya D. Sexual dysfunction in multiple sclerosis: gender differences. J Neurol Sci 2013;324.

50. Chakravarty EF, Murray ER, Kelman A, Farmer P. Pregnancy outcomes after maternal exposure to rituximab. Blood 2011;117:1499-1506.

51. Chen CS, Krishnakumar T, Rowles W, et al. Comparison of MS inflammatory activity in women using continuous versus cyclic combined oral contraceptives. Mult Scler Relat Disord 2020;41:101970-101970.

52. Chen YH, Lin HL, Lin HC. Does multiple sclerosis increase risk of adverse pregnancy outcomes? A population-based study. Multiple sclerosis (Houndmills, Basingstoke, England) 2009;15:606-612.

53. Christopherson Jeannine M, Moore K, Foley Frederick W, Warren Kenneth G. A comparison of written materials vs. materials and counselling for women with sexual dysfunction and multiple sclerosis. Journal of Clinical Nursing 2006;15:742-750.

54. Cil AP, Leventoglu A, Sonmezer M, Soylukoc R, Oktay K. Assessment of ovarian reserve and Doppler characteristics in patients with multiple sclerosis using immunomodulating drugs. Journal of the Turkish German Gynecology Association 2009;10:213-219.

55. Ciplea AI, Langer-Gould A, de Vries A, et al. Monoclonal antibody treatment during pregnancy and/or lactation in women with MS or neuromyelitis optica spectrum disorder. Neurology neuroimmunology & neuroinflammation 2020;7:2-2.

56. Ciplea AI, Langer-Gould A, Stahl A, et al. Safety of potential breast milk exposure to IFN-beta or glatiramer acetate: One-year infant outcomes. Neurology neuroimmunology & neuroinflammation 2020;7:7-7.

57. Cocco E, Sardu C, Gallo P, et al. Frequency and risk factors of mitoxantrone-induced amenorrhea in multiple sclerosis: the FEMIMS study. Multiple sclerosis (Houndmills, Basingstoke, England) 2008;14:1225-1233.

58. Confavreux C, Hutchinson M, Hours MM, Cortinovis-Tourniaire P, Moreau T, Pregnancy in Multiple Sclerosis Group. Rate of pregnancy-related relapse in multiple sclerosis. New England Journal of Medicine 1998;339:285-291.

59. Correale J, Farez Mauricio F, Ysrraelit Maria C. Increase in multiple sclerosis activity after assisted reproduction technology. Ann Neurol 2012;72:682-694.

60. Coyle PK, Sinclair SM, Scheuerle AE, et al. Final results from the Betaseron (interferon beta-1b) Pregnancy Registry: a prospective observational study of birth defects and pregnancy-related adverse events. BMJ Open 2014;4:e004536-e004536.

61. Cuello JP, Delgado FR, Dominguez JMG, et al. Time to pregnancy in multiple sclerosis: A case control comparative study. European Journal of Neurology 2017;24:199-199.

62. Cuello JP, Martinez G, M L, et al. Neurofilament light chain levels in pregnant multiple sclerosis patients: a prospective cohort study. European Journal of Neurology 2019;26:1200-1204.

63. Cuello JP, Martinez G, M L, Martin B, de Andres C. Multiple sclerosis and pregnancy: a single-centre prospective comparative study. Esclerosis multiple y embarazo: estudio unicentrico prospectivo y comparativo 2017;32:92-98.

64. Cuello JP, Martinez G, M L, et al. Cytokine profile during pregnancy predicts relapses during pregnancy and postpartum in multiple sclerosis. J Neurol Sci 2020;414 (no pa.

65. Cuello JP, Salgado C, Garcia D, et al. Pregnancy exposure to disease-modifying drugs in multiple sclerosis: a prospective study. Medicina Clinica 2020;154:214-217.

66. D'Hooghe M, Haentjens P, Nagels G, D'Hooghe T, De Keyser J. Menarche, oral contraceptives, pregnancy and progression of disability in relapsing onset and progressive onset multiple sclerosis. J Neurol 2012;259:855-861.

67. D'hooghe MB, Nagels G, Uitdehaag BMJ. Long-term effects of childbirth in MS. Journal of Neurology, Neurosurgery & Psychiatry 2010;81:38-41.

68. Dahl J, Myhr KM, Daltveit AK, Gilhus NE. Pregnancy, delivery and birth outcome in different stages of maternal multiple sclerosis. J Neurol 2008;255:623-627.

69. Dahl J, Myhr KM, Daltveit AK, Gilhus NE. Planned vaginal births in women with multiple sclerosis: delivery and birth outcome. Acta neurologica Scandinavica Supplementum 2006;183:51-54.

70. Dahl J, Myhr KM, Daltveit AK, Hoff JM, Gilhus NE. Pregnancy, delivery and birth outcome in women with multiple sclerosis. Neurology 2005;65:1961-1963.

71. Dahl J, Myhr KM, Daltveit AK, Skjaerven R, Gilhus NE. Is smoking an extra hazard in pregnant MS women? Findings from a population-based registry in Norway. European Journal of Neurology 2007;14:1113-1117.

72. Darija KT, Tatjana P, Goran T. Sexual dysfunction in multiple sclerosis: A 6-year follow-up study. J Neurol Sci 2015;358.

73. de Andres C, Fernandez-Paredes L, Tejera-Alhambra M, Alonso B, Ramos-Medina R, Sanchez-Ramon S. Activation of Blood CD3+CD56+CD8+ T Cells during Pregnancy and Multiple Sclerosis. Frontiers in Immunology 2017;8:196-196.

74. De Giglio L, Gasperini C, Tortorella C, Trojano M, Pozzilli C. Natalizumab discontinuation and disease restart in pregnancy: a case series. Acta Neurologica Scandinavica 2015;131:336-340.

75. De Giglio L, Marinelli F, Barletta V, et al. Relationship between prolactin plasma levels and white matter volume in women with multiple sclerosis. Mult Scler 2015;1):133-133.

76. De Giglio L, Marinelli F, Barletta Valeria T, et al. Effect on Cognition of Estroprogestins Combined with Interferon Beta in Multiple Sclerosis: Analysis of Secondary Outcomes from a Randomised Controlled Trial. CNS Drugs 2017;31:161-168.

77. De las Heras V, De Andres C, Tellez N, et al. Pregnancy in multiple sclerosis patients treated with immunomodulators prior to or during part of the pregnancy: A descriptive study in the Spanish population. Mult Scler 2007;13:981-984.

78. de Seze J, Chapelotte M, Delalande S, Ferriby D, Stojkovic T, Vermersch P. Intravenous corticosteroids in the postpartum period for reduction of acute exacerbations in multiple sclerosis. Multiple sclerosis (Houndmills, Basingstoke, England) 2004;10:596-597.

79. Deatrick JA, Brennan D, Cameron ME. Mothers with multiple sclerosis and their children: effects of fatigue and exacerbations on maternal support. Nurs Res 1998;47:205-210.

80. Dehghan-Nayeri N, Khakbazan Z, Ghafoori F, Nabavi SM. Sexual dysfunction levels in iranian women suffering from multiple sclerosis. Mult Scler Relat Disord 2017;12:49-53.

81. Demirkiran M, Sarica Y, Uguz S, Yerdelen D, Aslan K. Multiple sclerosis patients with and without sexual dysfunction: are there any differences? Mult Scler 2006;12.

82. Demortiere S, Rico A, Maarouf A, Boutiere C, Pelletier J, Audoin B. Maintenance of natalizumab during the first trimester of pregnancy in active multiple sclerosis. Multiple Sclerosis Journal 2020.

83. Diareme S, Tsiantis J, Kolaitis G, et al. Emotional and behavioural difficulties in children of parents with multiple sclerosis: a controlled study in Greece. European child & adolescent psychiatry 2006;15:309-318.

84. Dobos K, Healy B, Houtchens M. Access to Preventive Health Care in Severely Disabled Women with Multiple Sclerosis. International Journal of MS Care 2015;17:200-205.

85. Doosti R, Togha M, Moghadasi AN, et al. Evaluation of the risk of cervical cancer in patients with Multiple Sclerosis treated with cytotoxic agents: A cohort study. Iranian Journal of Neurology 2018;17:64-70.

86. Dugue PA, Rebolj M, Hallas J, Garred P, Lynge E. Risk of cervical cancer in women with autoimmune diseases, in relation with their use of immunosuppressants and screening: Population-based cohort study. International Journal of Cancer 2014;136:E711-E719.

87. Durufle A, Nicolas B, Petrilli S, et al. Effects of pregnancy and childbirth on the incidence of urinary disorders in multiple sclerosis. Clinical & Experimental Obstetrics & Gynecology 2006;33:215-218.

88. Durufle A, Petrilli S, Nicolas B, et al. Effects of pregnancy and child birth on urinary symptoms and urodynamics in women with multiple sclerosis. International Urogynecology Journal 2006;17:352-355.

89. Ebrahimi N, Herbstritt S, Gold R, Amezcua L, Koren G, Hellwig K. Pregnancy and fetal outcomes following natalizumab exposure in pregnancy. A prospective, controlled observational study. Multiple Sclerosis Journal 2015;21:198-205.

90. Eftekhari E, Etemadifar M, Mostahfezian M, Zafari A. Effects of resistance training and vibration on hormonal changes in female patients with multiple sclerosis. Neurology Asia 2014;19:63-67.

91. Egerod I, Wulff K, Petersen MC. Experiences and informational needs on sexual health in people with epilepsy or multiple sclerosis: A focus group investigation. Journal of Clinical Nursing 2018;27:2868-2876.

92. Ehrlich S, Haas J, Zipp F, Infante-Duarte C. Serum levels of soluble CD95 are not associated with amelioration of multiple sclerosis during pregnancy. J Neurol Sci 2007;252:83-87.

93. Etemadifar M, Janghorbani M. Efficacy of high-dose vitamin D3 supplementation in vitamin D deficient pregnant women with multiple sclerosis: Preliminary findings of a randomized-controlled trial. Iranian Journal of Neurology 2015;14:67-73.

94. Falaschi P, Martocchia A, Proietti A, D'Urso R, Antonini G. High incidence of hyperandrogenism-related clinical signs in patients with multiple sclerosis. Neuro Endocrinol Lett 2001;22:248-250.

95. Fares J, Nassar AH, Gebeily S, Kobeissy F, Fares Y. Pregnancy outcomes in Lebanese women with multiple sclerosis (the LeMS study): A prospective multicentre study. BMJ Open 2016;6:e011210.

96. Fernandez L, Klajn D, Acion L, et al. Epidemiological characteristics of pregnancy, delivery, and birth outcome in women with multiple sclerosis in Argentina (EMEMAR study). Multiple sclerosis (Houndmills, Basingstoke, England) 2009;15:555-562.

97. Ferraro D, Simone AM, Adani G, et al. Definitive childlessness in women with multiple sclerosis: a multicenter study. Neurological Sciences 2017;38:1453-1459.

98. Finkelsztejn A, Fragoso YD, Ferreira MLB, et al. The Brazilian database on pregnancy in multiple sclerosis. Clinical Neurology and Neurosurgery 2011;113:277-280.

99. Firdolas F, Ozan T, Onur R, Bulut S, Orhan I. Evaluation of sexual function in women at two stages of multiple sclerosis. World journal of urology 2013;31:929-933.

100. Fong A, Chau CT, Quant C, Duffy J, Pan D, Ogunyemi DA. Multiple sclerosis in pregnancy: prevalence, sociodemographic features, and obstetrical outcomes. Journal of Maternal-Fetal & Neonatal Medicine 2018;31:382-387.

101. Foroughipour A, Norbakhsh V, Najafabadi SH, Meamar R. Evaluating sex hormone levels in reproductive age women with multiple sclerosis and their relationship with disease severity. Journal of Research in Medical Sciences 2012;17:882-885.

102. Fragala E, Privitera S, Giardina R, et al. Determinants of sexual impairment in multiple sclerosis in male and female patients with lower urinary tract dysfunction: results from an Italian cross-sectional study. The journal of sexual medicine 2014;11:2406-2413.

103. Fragoso Yara D, Adoni T, Alves-Leon Soniza V, et al. Long-term effects of exposure to disease-modifying drugs in the offspring of mothers with multiple sclerosis: a retrospective chart review. CNS Drugs 2013;27:955-961.

104. Fragoso Yara D, Finkelsztejn A, Kaimen-Maciel Damacio R, et al. Long-term use of glatiramer acetate by 11 pregnant women with multiple sclerosis: a retrospective, multicentre case series. CNS Drugs 2010;24:969-976.

105. Fragoso Yara D, Adoni T, Alves-Leon Soniza V, et al. Postpartum Treatment With Immunoglobulin Does Not Prevent Relapses of Multiple Sclerosis in the Mother. Health care for women international 2015;36:1072-1080.

106. Fragoso Yara D, Boggild M, Macias-Islas Miguel A, et al. The effects of long-term exposure to disease-modifying drugs during pregnancy in multiple sclerosis. Clinical Neurology and Neurosurgery 2013;115:154-159.

107. Fragoso Yara D, Finkelsztejn A, Comini-Frota Elizabeth R, et al. Pregnancy and multiple sclerosis: the initial results from a Brazilian database. Arquivos de neuro-psiquiatria 2009;67:657-660.

108. Frau J, Coghe G, Casanova P, et al. Pregnancy planning and outcomes in patients with multiple sclerosis after mitoxantrone therapy: a monocentre assessment. European Journal of Neurology 2018;25:1063-1068.

109. Friend S, Richman S, Bloomgren G, Cristiano LM, Wenten M. Evaluation of pregnancy outcomes from the Tysabri (natalizumab) pregnancy exposure registry: A global, observational, follow-up study. BMC Neurology 2016;16:150-150.

110. Frith JA, McLeod JG. Pregnancy and multiple sclerosis. Journal of neurology, neurosurgery, and psychiatry 1988;51:495-498.

111. Frith JA, McLeod JG. Pregnancy and multiple sclerosis. An Australian perspective. Clinical and experimental neurology 1987;24:1-4.

112. Gagliardi BA. The experience of sexuality for individuals living with multiple sclerosis. Journal of Clinical Nursing 2003;12:571-578.

113. Gava G, Salvi F, Bartolomei I, et al. Long-term impact of combined oral contraceptive use on disability progression in relapsing-remitting multiple sclerosis. Endocrine Reviews 2014;34.

114. Gava G, Visconti M, Salvi F, Bartolomei I, Seracchioli R, Meriggiola MC. Prevalence and Psychopathological Determinants of Sexual Dysfunction and Related Distress in Women With and Without Multiple Sclerosis. Journal of Sexual Medicine 2019;16:833-842.

115. Gava G, Bartolomei I, Costantino A, et al. Long-term influence of combined oral contraceptive use on the clinical course of relapsing-remitting multiple sclerosis. Fertility and sterility 2014;102:116-122.

116. Geissbuhler Y, Rezaallah B, Moore A. An alternative to product-specific pregnancy registries? PRIM; PRegnancy outcomes Intensive Monitoring. Reproductive Toxicology 2020;94:13-21.

117. Geissbuhler Y, Vile J, Koren G, et al. Evaluation of pregnancy outcomes in patients with multiple sclerosis after fingolimod exposure. Therapeutic Advances in Neurological Disorders 2018;11.

118. Ghafoori F, Dehghan-Nayeri N, Khakbazan Z, Hedayatnejad M, Nabavi SM. Pregnancy and Motherhood Concerns Surrounding Women with Multiple Sclerosis: A Qualitative Content Analysis. International Journal of Community Based Nursing & Midwifery 2020;8:2-11.

119. Gharesi-Fard B, Zare M, Kamali-Sarvestani E. The Reaction of Placental GRP78 Protein with Sera from Women with Multiple Sclerosis. Iranian Journal Of Immunology: IJI 2017;14:306-315.

120. Ghiasian M, Nouri M, Moghadasi AN, Ghaffari M. Effect of pregnancy and exclusive breastfeeding on multiple sclerosis relapse rate and degree of disability within two years after delivery. Clinical Neurology & Neurosurgery 2020;194:105829-105829.

121. Giannantoni A, Proietti S, Giusti G, et al. OnabotulinumtoxinA intradetrusorial injections improve sexual function in female patients affected by multiple sclerosis: preliminary results. World journal of urology 2015;33:2095-2101.

122. Giannini M, Portaccio E, Ghezzi A, et al. Pregnancy and fetal outcomes after Glatiramer Acetate exposure in patients with multiple sclerosis: a prospective observational multicentric study. BMC Neurology 2012;12:124.

123. Gilli F, Lindberg Raija LP, Valentino P, et al. Learning from nature: pregnancy changes the expression of inflammation-related genes in patients with multiple sclerosis. PLoS One 2010;5:e8962-e8962.

124. Gilmore W, Arias M, Stroud N, Stek A, McCarthy Kathleen A, Correale J. Preliminary studies of cytokine secretion patterns associated with pregnancy in MS patients. J Neurol Sci 2004;224:69-76.

125. Giovannoni G, Galazka A, Schick R, et al. Pregnancy Outcomes During the Clinical Development Program of Cladribine in Multiple Sclerosis: An Integrated Analysis of Safety. Drug Safety 2020;43:635-643.

126. Gold R, Phillips JT, Havrdova E, et al. Delayed-release dimethyl fumarate and pregnancy: Preclinical studies and pregnancy outcomes from clinical trials and postmarketing experience. Mult Scler 2015;20:435-435.

127. Gold R, Stefoski D, Selmaj K, et al. Pregnancy Experience: Nonclinical Studies and Pregnancy Outcomes in the Daclizumab Clinical Study Program. Neurology and Therapy 2016;5:169-182.

128. Goldacre A, Pakpoor J, Goldacre M. Perinatal characteristics and obstetric complications in mothers with multiple sclerosis: Record-linkage study. Mult Scler Relat Disord 2017;12:4-8.

129. Graves JS, Henry RG, Cree BAC, et al. Ovarian aging is associated with gray matter volume and disability in women with MS. Neurology 2018;90:e254-e260.

130. Graziano F, Calandri E, Borghi M, Bonino S. Adjustment to multiple sclerosis and identity satisfaction among newly diagnosed women: what role does motherhood play? Women & Health 2020;60:271-283.

131. Grinsted L, Heltberg A, Hagen C, Djursing H. Serum sex hormone and gonadotropin concentrations in premenopausal women with multiple sclerosis. Journal of Internal Medicine 1989;226:241-244.

132. Gulick Elsie E. Postpartum functioning in mothers with multiple sclerosis. Western journal of nursing research 2007;29:512-589.

133. Gulick Elsie E. Adaptation of the postpartum support questionnaire for mothers with multiple sclerosis. Research in Nursing & Health 2003;26:30-39.

134. Gulick Elsie E, Johnson S. Infant health of mothers with multiple sclerosis. Western journal of nursing research 2004;26:632-649.

135. Gulick EE, Kim S. Postpartum Emotional Distress in Mothers With Multiple Sclerosis. Journal of Obstetric, Gynecologic & Neonatal Nursing 2004;33:729-738.

136. Guven Y, Ozakbas S. Effect of hormonal changes on the neurological status in the menstrual cycle of patient with multiple sclerosis. Clinical Neurology & Neurosurgery 2019;186:105499-105499.

137. Haas J. High dose IVIG in the post partum period for prevention of exacerbations in MS. Multiple Sclerosis Journal 2000;6 Suppl 2:S18-S33.

138. Hakkarainen KM, Juuti R, Burkill S, et al. Pregnancy outcomes after exposure to interferon beta: a register-based cohort study among women with MS in Finland and Sweden. Therapeutic Advances in Neurological Disorders 2020;13.

139. Harazim H, Janku P, Zelinkova H, Frank K, Dufek M, Stourac P. Obstetric anesthesia/analgesia does not affect disease course in multiple sclerosis: 10-year retrospective cohort study. Brain Behav 2018;8:e01082.

140. Harrison T, Stuifbergen A. Disability, social support, and concern for children: depression in mothers with multiple sclerosis. Journal of obstetric, gynecologic, and neonatal nursing : JOGNN / NAACOG 2002;31:444-453.

141. Hellwig K, Beste C, Schimrigk S, Chan A. Immunomodulation and postpartum relapses in patients with multiple sclerosis. Therapeutic Advances in Neurological Disorders 2009;2:7-11.

142. Hellwig K, Duarte C, Wicklein EM, Bhatti A, Adamo A. Pregnancy outcomes from the global pharmacovigilance database on interferon beta-1b exposure. Therapeutic Advances in Neurological Disorders 2020;13.

143. Hellwig K, Geissbuehler Y, Sabidó M, et al. Pregnancy outcomes in interferon-beta-exposed patients with multiple sclerosis: results from the European Interferon-beta Pregnancy Registry. J Neurol 2020:10.1007/s00415-00020-09762-y.

144. Hellwig K, Haghikia A, Rockhoff M, Gold R. Multiple sclerosis and pregnancy: Experience from a nationwide database in Germany. Therapeutic Advances in Neurological Disorders 2012;5:247-253.

145. Hellwig K, Rockhoff M, Herbstritt S, et al. Exclusive Breastfeeding and the Effect on Postpartum Multiple Sclerosis Relapses. JAMA Neurology 2015;72:1132-1138.

146. Herbstritt S, Langer-Gould A, Rockhoff M, et al. Glatiramer acetate during early pregnancy: A prospective cohort study. Multiple sclerosis (Houndmills, Basingstoke, England) 2016;22:810-816.

147. Hocaloski S, Elliott S, Brotto LA, Breckon E, McBride K. A Mindfulness Psychoeducational Group Intervention Targeting Sexual Adjustment for Women with Multiple Sclerosis and Spinal Cord Injury: A Pilot Study. Sexuality and Disability 2016;34:183-198.

148. Hoffmann F, Kraft A, Heigl F, et al. Tryptophan immunoadsorption during pregnancy and breastfeeding in patients with acute relapse of multiple sclerosis and neuromyelitis optica. Therapeutic Advances in Neurological Disorders 2018;11.

149. Horvat L, Brecl J, Jerse J, et al. Intravenous immunoglobulins for the prevention of postpartum relapses in multiple sclerosis. Mult Scler Relat Disord 2020;38 (no pag.

150. Hosl KM, Deutsch M, Wang R, et al. Sexual Dysfunction Seems to Trigger Depression in Female Multiple Sclerosis Patients. European neurology 2018;80:34-41.

151. Houtchens M, Bove R, Healy B, et al. MRI activity in MS and completed pregnancy: Data from a tertiary academic center. Neurology 2020;7.

152. Houtchens MK, Edwards NC, Hayward B, Mahony MC, Phillips AL. Live birth rates, infertility diagnosis, and infertility treatment in women with and without multiple sclerosis: Data from an administrative claims database. Mult Scler Relat Disord 2020;46 (no pag.

153. Hughes SE, Spelman T, Gray OM, et al. Predictors and dynamics of postpartum relapses in women with multiple sclerosis. Multiple Sclerosis Journal 2014;20:739-746.

154. Hulter BM, Lundberg PO. Sexual function in women with advanced multiple sclerosis. Journal of Neurology Neurosurgery and Psychiatry 1995;59:83-86.

155. Jawahar R, Oh U, Eaton C, Wright N, Tindle H, Lapane KL. Association between smoking and health outcomes in postmenopausal women living with multiple sclerosis. Multiple Sclerosis International 2014;2014:686045-686045.

156. Jesus-Ribeiro J, Correia I, Martins AI, et al. Pregnancy in Multiple Sclerosis: A Portuguese cohort study. Mult Scler Relat Disord 2017;17:63-68.

157. Jolving LR, Larsen MD, Fedder J, Norgard BM. Live birth in women with multiple sclerosis receiving assisted reproduction. Reproductive BioMedicine Online 2020;40:711-718.

158. Kamm CP, Muehl S, Mircsof D, et al. Role of family planning in women with multiple sclerosis in Switzerland: Results of the women with multiple sclerosis patient survey. Frontiers in Neurology 2018;9 (OCT) (n.

159. Karlsson G, Francis G, Koren G, et al. Pregnancy outcomes in the clinical development program of fingolimod in multiple sclerosis. Neurology 2014;82:674-680.

160. Karp I, Manganas A, Sylvestre M-P, Ho A, Roger E, Duquette P. Does pregnancy alter the long-term course of multiple sclerosis? Ann Epidemiol 2014;24:504-508.e502.

161. Kasatkin DS, Spirin NN, Vinogradova TV, Shitova AS. Effects of the Treatment of Mothers with Multiple Sclerosis Using Disease-Modifying Drugs on Child Development. Neuroscience and Behavioral Physiology 2018;48:864-869.

162. Kelly Victoria M, Nelson Lorene M, Chakravarty Eliza F. Obstetric outcomes in women with multiple sclerosis and epilepsy. Neurology 2009;73:1831-1836.

163. Kempe P, Eklund D, Hallin A, et al. Immune profile in relation to sex steroid cyclicity in healthy women and women with multiple sclerosis. Journal of Reproductive Immunology 2018;126:53-59.

164. Kempe P, Hammar M, Brynhildsen J. Symptoms of multiple sclerosis during use of combined hormonal contraception. European journal of obstetrics, gynecology, and reproductive biology 2015;193:1-4.

165. Keyhanian K, Davoudi V, Etemadifar M, Amin M. Better prognosis of multiple sclerosis in patients who experienced a full-term pregnancy. European neurology 2011;68:150-155.

166. Khalid F, Healy BC, Dupuy SL, et al. Quantitative MRI analysis of cerebral lesions and atrophy in post-partum patients with multiple sclerosis. J Neurol Sci 2018;392:94-99.

167. Khayambashi S, Salter A, Tyry T, Cutter GR, Fox RJ, Marrie RA. Gender identity and sexual orientation affect health care satisfaction, but not utilization, in persons with Multiple Sclerosis. Mult Scler Relat Disord 2020;37:101440.

168. Kieseier BC, Benamor M. Pregnancy Outcomes Following Maternal and Paternal Exposure to Teriflunomide During Treatment for Relapsing-Remitting Multiple Sclerosis. Neurology and Therapy 2014;3:133-138.

169. Kilic B, Unver V, Bolu A, Demirkaya S. Sexual dysfunction and coping strategies in multiple sclerosis patients. Sexuality and Disability 2012;30:3-13.

170. Kinga M, Balasa R. Effect of serum 25(OH) D level, cigarette smoking and oral contraceptive use on clinical course of relapsing-remitting multiple sclerosis in a group of female patients. Romanian Journal of Neurology/ Revista Romana de Neurologie 2015;14:214-2018.

171. Kleerekooper I, Van Kempen Z, Leurs CE, et al. Disease activity following pregnancy-related discontinuation of natalizumab in multiple sclerosis. Multiple Sclerosis Journal 2017;23:666-667.

172. Koch M, Uyttenboogaart M, Heersema D, Steen C, De Keyser J. Parity and secondary progression in multiple sclerosis. Journal of neurology, neurosurgery, and psychiatry 2009;80:676-678.

173. Koch T, Kelly S. Understanding what is important for women who live with multiple sclerosis. The Australian journal of holistic nursing 1999;6:14-24.

174. Koch T, Kralik D, Eastwood S. Constructions of sexuality for women living with multiple sclerosis. Journal of advanced nursing 2002;39:137-145.

175. Koltuniuk A, Przestrzelska M, Karnas A, Rosinczuk J. The Association Between Sexual Disorders and the Quality of Life of Woman Patients With Multiple Sclerosis: Findings of a Prospective, Observational, and Cross-Sectional Survey. Sexual Medicine 2020;8:297-306.

176. Tzitzika M. Female sexual dysfunction among greek women with multiple sclerosis: Correlations with organic and psychological factors. Multiple Sclerosis Journal 2018;24 (6):854-854.

177. Kosmala-Anderson J, Wallace LM. A qualitative study of the childbearing experience of women living with multiple sclerosis. Disability and Rehabilitation 2013;35:976-981.

178. Kralik D, Koch T, Eastwood S. The salience of the body: transition in sexual self-identity for women living with multiple sclerosis. Journal of advanced nursing 2003;42:11-20.

179. De Giglio L, Federici S, Ruggieri S, et al. Cesarean section in women with MS: A choice or a need? Mult Scler Relat Disord 2020;38:101867.

180. Labarrere CA, Catoggio LJ, Mullen EG, Althabe OH. Placental lesions in maternal autoimmune diseases. American journal of reproductive immunology and microbiology : AJRIM 1986;12:78-86.

181. Ladeira F, Salavisa M, Caetano A, Barbosa R, Sa F, Correia AS. The Influence of Menopause in Multiple Sclerosis Course: A Longitudinal Cohort Study. European neurology 2018;80:223-227.

182. Lai W, Kinoshita M, Peng A, et al. Does pregnancy affect women with multiple sclerosis? A prospective study in Western China. Journal of Neuroimmunology 2018;321:24-28.

183. Landi D, Ragonese P, Prosperini L, et al. Abortion induces reactivation of inflammation in relapsing-remitting multiple sclerosis. Journal of Neurology, Neurosurgery and Psychiatry 2018;89:1272-1278.

184. Langer-Gould A, Smith JB, Albers KB, et al. Pregnancy-related relapses and breastfeeding in a contemporary multiple sclerosis cohort. Neurology 2020;94:e1939-e1949.

185. Langer-Gould A, Gupta R, Huang S, et al. Interferon-gamma-producing T cells, pregnancy, and postpartum relapses of multiple sclerosis. Arch Neurol 2010;67:51-57.

186. Langer-Gould A, Huang S, Van Den Eeden Stephen K, et al. Vitamin D, pregnancy, breastfeeding, and postpartum multiple sclerosis relapses. Arch Neurol 2011;68:310-313.

187. Langer-Gould A, Huang SM, Gupta R, et al. Exclusive breastfeeding and the risk of postpartum relapses in women with multiple sclerosis. Arch Neurol 2009;66:958-963.

188. Laplaud D-A, Leray E, BarriÃ¨re P, Wiertlewski S, Moreau T. Increase in multiple sclerosis relapse rate following in vitro fertilization. Neurology 2006;66:1280-1281.

189. Lavie C, Rollot F, Durand-Dubief F, et al. Neuraxial analgesia is not associated with an increased risk of post-partum relapses in MS. Multiple Sclerosis Journal 2019;25:591-600.

190. Lavorgna L, Esposito S, Lanzillo R, et al. Factors interfering with parenthood decision-making in an Italian sample of people with multiple sclerosis: an exploratory online survey. J Neurol 2019;266:707-716.

191. Lavorgna L, Moccia M, Russo A, et al. Health-care disparities stemming from sexual orientation of Italian patients with Multiple Sclerosis: A cross-sectional web-based study. Mult Scler Relat Disord 2017;13:28-32.

192. Lebrun C, Le Page E, Kantarci O, et al. Impact of pregnancy on conversion to clinically isolated syndrome in a radiologically isolated syndrome cohort. Multiple Sclerosis Journal 2012;18:1297-1302.

193. Lew-Starowicz M, Rola R. Prevalence of sexual dysfunctions among women with multiple sclerosis. Sexuality and Disability 2013;31:141-153.

194. Lew-Starowicz M, Rola R. Correlates of sexual function in male and female patients with multiple sclerosis. J Sex Med 2014;11.

195. Linssen WHJP, Notermans NC, Hommes OR, Rolland R. Amenorrhea after immunosuppressive treatment of multiple sclerosis. Acta Neurologica Scandinavica 1987;76:204-209.

196. Lombardi G, Celso M, Bartelli M, Cilotti A, Del Popolo G. Female sexual dysfunction and hormonal status in multiple sclerosis patients. The journal of sexual medicine 2016;8:1138-1146.

197. Lopez C, Comabella M, Tintore M, Sastre-Garriga J, Montalban X. Variations in chemokine receptor and cytokine expression during pregnancy in multiple sclerosis patients. Multiple Sclerosis Journal 2006;12:421-427.

198. Lu E, Dahlgren L, Sadovnick A, Sayao A, Synnes A, Tremlett H. Perinatal outcomes in women with multiple sclerosis exposed to disease-modifying drugs. Multiple Sclerosis Journal 2012;18:460-467.

199. Lu E, Zhao Y, Zhu F, et al. Birth hospitalization in mothers with multiple sclerosis and their newborns. Neurology 2013;80:447-452.

200. Lu E, Zhu F, van der Kop M, et al. Labor induction and augmentation in women with multiple sclerosis. Multiple sclerosis (Houndmills, Basingstoke, England) 2013;19:1182-1189.

201. Lu E, Zhao Y, Dahlgren L, et al. Obstetrical epidural and spinal anesthesia in multiple sclerosis. J Neurol 2013;260:2620-2628.

202. MacDonald SC, Hernan MA, McElrath TF, Hernandez-Diaz S. Assessment of recording bias in pregnancy studies using health care databases: An application to neurologic conditions. Paediatric and Perinatal Epidemiology 2018;32:281-286.

203. MacDonald SC, McElrath TF, Hernandez-Diaz S. Use and safety of disease-modifying therapy in pregnant women with multiple sclerosis. Pharmacoepidemiology & Drug Safety 2019;28:556-560.

204. MacDonald SC, McElrath TF, Hernandez-Diaz S. Pregnancy Outcomes in Women With Multiple Sclerosis. Am J Epidemiol 2019;188:57-66.

205. Maciejewska M, Snarski E, Wiktor-Jedrzejczak W. A preliminary online study on menstruation recovery in women after autologous hematopoietic stem cell transplant for autoimmune diseases. Experimental and Clinical Transplantation 2016;14:665-669.

206. MacKenzie-Graham A, Brook J, Kurth F, et al. Estriol-mediated neuroprotection in multiple sclerosis visualized by voxel-based morphometry. Multiple Sclerosis Journal 2018;24 (2 Supp:483-484.

207. Maclurg K, Reilly P, Hawkins S. Participation in general practice health screening by people with multiple sclerosis. Br J Gen Pract 2004;54:853-855.

208. Mahlanza TD, Manieri MC, Klawiter EC, et al. Prospective growth and developmental outcomes in infants born to mothers with multiple sclerosis. Multiple Sclerosis Journal 2020.

209. Mallucci G, Beneventi F, Bergamaschi R, et al. Circulating endothelial progenitor cells during pregnancy in multiple sclerosis. Neurological Sciences 2021.

210. Marck CH, Jelinek PL, Weiland TJ, et al. Sexual function in multiple sclerosis and associations with demographic, disease and lifestyle characteristics: an international cross-sectional study. BMC Neurology 2016;16:210.

211. Masera S, Cavalla P, Prosperini L, et al. Parity is associated with a longer time to reach irreversible disability milestones in women with multiple sclerosis. Multiple Sclerosis Journal 2015;21:1291-1297.

212. May AE, Fombon FN, Francis S. UK registry of high-risk obstetric anaesthesia: report on neurological disease. International journal of obstetric anesthesia 2008;17:31-36.

213. Mehrpour M, Taherian F, Arfa-Fatollahkhani P, Moghadasi AN, Mokhtar S, Keivani H. Serum anti-mullerian hormone levels in multiple sclerosis - A multicenter case-control study. Ceska a Slovenska Neurologie a Neurochirurgie 2018;81:199-202.

214. Mehta D, Wani S, Wallace L, Henders AK, Wray NR, McCombe PA. Cumulative influence of parity-related genomic changes in multiple sclerosis. Journal of Neuroimmunology 2019;328:38-49.

215. Merghati-Khoei E, Qaderi K, Amini L, Korte JE. Sexual problems among women with multiple sclerosis. J Neurol Sci 2013;331:81-85.

216. Michel L, Foucher Y, Vukusic S, et al. Increased risk of multiple sclerosis relapse after in vitro fertilisation. Journal of neurology, neurosurgery, and psychiatry 2012;83:796-802.

217. Mirmosayyeb O, Badihian S, Manouchehri N, et al. The interplay of multiple sclerosis and menstrual cycle: Which one affects the other one? Mult Scler Relat Disord 2018;21:46-50.

218. Moberg JY, Magyari M, Koch-Henriksen N, Thygesen LC, Laursen B, Soelberg S. Educational achievements of children of parents with multiple sclerosis: A nationwide register-based cohort study. J Neurol 2016;263:2229-2237.

219. Moberg Julie Y, Laursen B, Koch-Henriksen N, et al. Employment, disability pension and income for children with parental multiple sclerosis. Multiple Sclerosis Journal 2017;23:1148-1156.

220. Mohammadi K, Rahnama P, Rafei Z, Ebrahimi-Aveh SM, Montazeri A. Factors associated with intimacy and sexuality among young women with multiple sclerosis. Reproductive Health 2020;17 (1) (no.

221. Mueller BA, Zhang J, Critchlow CW. Birth outcomes and need for hospitalization after delivery among women with multiple sclerosis. American Journal of Obstetrics and Gynecology 2002;186:446-452.

222. Nabavi SM, Koupai SA, Nejati MR, Garshasbi E, Jalali MR. Menstrual irregularities and related plasma hormone levels in multiple sclerosis patients treated with beta interferone. Acta Medica Iranica 2010;48:36-41.

223. Najafidoulatabad S, Mohebbi Z, Nooryan K. Yoga effects on physical activity and sexual satisfaction among the Iranian women with multiple sclerosis: a randomized controlled trial. African journal of traditional, complementary, and alternative medicines : AJTCAM 2014;11:78-82.

224. Nasimbera A, Rosales J, Silva B, et al. Everything you always wanted to know about sex and neurology: Neurological disability and sexuality. Arquivos de Neuro-Psiquiatria 2018;76:430-435.

225. Nelson LM, Franklin GM, Jones MC, et al. Risk of Multiple Sclerosis Exacerbation During Pregnancy and Breast-feeding. JAMA 1988;259:3441-3443.

226. Neuteboom Rinze F, Verbraak E, Wierenga-Wolf Annet F, et al. Pregnancy-induced fluctuations in functional T-cell subsets in multiple sclerosis patients. Multiple Sclerosis Journal 2010;16:1073-1078.

227. Neuteboom RF, Janssens ACJW, Siepman TAM, et al. Pregnancy in multiple sclerosis: clinical and self-report scales. J Neurol 2012;259:311-317.

228. Neuteboom RF, Verbraak E, Voerman JSA, et al. Serum leptin levels during pregnancy in multiple sclerosis. Multiple Sclerosis Journal 2009;15:907-912.

229. Neuteboom Rinze F, Verbraak E, Wierenga-Wolf Annet F, et al. The monocyte transcriptome during pregnancy in multiple sclerosis: prominent expression of the Fc-receptor CD64. Multiple Sclerosis Journal 2010;17:389-396.

230. Nguyen A-L, Havrdova EK, Horakova D, et al. Incidence of pregnancy and disease-modifying therapy exposure trends in women with multiple sclerosis: A contemporary cohort study. Mult Scler Relat Disord 2019;28:235-243.

231. Novo A, Castelo J, de Sousa A, et al. Pregnancy outcomes in Portuguese women with multiple sclerosis: The PREGNIMS study. Mult Scler Relat Disord 2019;28:172-176.

232. Oh J, Achiron A, Celius EG, et al. Pregnancy outcomes and postpartum relapse rates in women with RRMS treated with alemtuzumab in the phase 2 and 3 clinical development program over 16 years. Mult Scler Relat Disord 2020;43 (no pag.

233. Orasanu B, Frasure H, Wyman A, Mahajan ST. Sexual dysfunction in patients with multiple sclerosis. Mult Scler Relat Disord 2013;2.

234. Orvieto R, Achiron R, Rotstein Z, Noy S, Bar-Hava I, Achiron A. Pregnancy and multiple sclerosis: a 2-year experience. European Journal of Obstetrics & Gynecology and Reproductive Biology 1999;82:191-194.

235. Paavilainen T, Kurki T, Parkkola R, et al. Magnetic resonance imaging of the brain used to detect early post‐partum activation of multiple sclerosis. European Journal of Neurology 2007;14:1216-1221.

236. Paavilainen T, Kurki T, Farkkila M, Salonen O, Parkkola R, Airas L. Lower brain diffusivity in postpartum period compared to late pregnancy: results from a prospective imaging study of multiple sclerosis patients. Neuroradiology 2012;54:823-828.

237. Paraska K. Relationship between expanded health belief model variables and mammography screening adherence in women with multiple sclerosis: a pilot study. International Journal of MS Care 2012;14:142-147.

238. Parton C, Katz T, Ussher JM. 'Normal' and 'failing' mothers: Women's constructions of maternal subjectivity while living with multiple sclerosis. Health: an Interdisciplinary Journal for the Social Study of Health, Illness & Medicine 2019;23:516-532.

239. Pasic H, Vujevic L, Bosnjak P, et al. Sexual Dysfunction and Depression in Patients with Multiple Sclerosis in Croatia. Psychiatria Danubina 2019;31:831-838.

240. Pasto L, Portaccio E, Ghezzi A, et al. Epidural analgesia and cesarean delivery in multiple sclerosis post-partum relapses: The Italian cohort study. BMC Neurology 2012;12:165.

241. Patti F, Cavallaro T, Lo F, et al. Is in utero early-exposure to interferon beta a risk factor for pregnancy outcomes in multiple sclerosis? J Neurol 2008;255:1250-1253.

242. Patti F, Messina S, D'Amico E, Lo F, Zappia M. Pregnancy outcomes in multiple sclerosis patients previously treated with cyclophosphamide. Acta Neurologica Scandinavica 2014;130:e41-44.

243. Pauliat E, Onken M, Weber-Schoendorfer C, et al. Pregnancy outcome following first-trimester exposure to fingolimod: A collaborative ENTIS study. Multiple Sclerosis Journal 2020.

244. Payne D, McPherson KM. Becoming mothers. Multiple sclerosis and motherhood: a qualitative study. Disability and Rehabilitation 2010;32:629-638.

245. Pebdani RN, Johnson KL, Amtmann D, Bamer AM, Wundes A. Experiences and Perspectives of Pregnancy in Women with Multiple Sclerosis. Sexuality and Disability 2015;33:47-52.

246. Pelfrey CM, Moldovan IR, Cotleur AC, Zamor N, Rudick RA. Effects of sex hormones on costimulatory molecule expression in multiple sclerosis. Journal of Neuroimmunology 2005;167:190-203.

247. Popek M, Grabowska-Popek A, Mitosek-Szewczyk K, Stelmasiak Z. Is multiple sclerosis only a neurological problem? Evaluation of sexual dysfunctions in a group of multiple sclerosis patients. Medical Studies/Studia Medyczne 2018;34:127-132.

248. Portaccio E, Ghezzi A, Hakiki B, et al. Breastfeeding is not related to postpartum relapses in multiple sclerosis. Neurology 2011;77:145-150.

249. Portaccio E, Annovazzi P, Ghezzi A, et al. Pregnancy decision-making in multiple sclerosis women treated with natalizumab: 1 fetal risks. Neurology 2018;Epub Feb 7.

250. Portaccio E, Moiola L, Martinelli V, et al. Pregnancy decision-making in multiple sclerosis women treated with natalizumab: 2 maternal risks. Neurology 2018;Epub Feb 7.

251. Portaccio E, Ghezzi A, Hakiki B. Postpartum relapses increase the risk of disability progression in multiple sclerosis: the role of disease-modifying drugs. Journal of Neurology Neurosurgery and Psychiatry 2014;85:845-850.

252. Poser S, Poser W. Multiple sclerosis and gestation. Neurology 1983;33:1422-.

253. Pozzilli C, De Giglio L, Barletta VT, et al. Oral contraceptives combined with interferon b in multiple sclerosis. Neurology: Neuroimmunology and Neuroinflammation 2015;2:e120-e120.

254. Proschmann U, Thomas K, Thiel S, Hellwig K, Ziemssen T. Natalizumab during pregnancy and lactation. Mult Scler 2018;24:1627-1634.

255. Prunty M, Sharpe L, Butow P, Fulcher G. The motherhood choice: Themes arising in the decision-making process for women with multiple sclerosis. Multiple Sclerosis Journal 2008;14:701-704.

256. Prunty MC, Sharpe L, Butow P, Fulcher G. The motherhood choice: A decision aid for women with multiple sclerosis. Patient Education and Counseling 2008;71:108-115.

257. Ramagopalan S, Yee I, Byrnes J, Guimond C, Ebers G, Sadovnick D. Term pregnancies and the clinical characteristics of multiple sclerosis: a population based study. Journal of neurology, neurosurgery, and psychiatry 2012;83:793-795.

258. Ramien C, Yusko EC, Engler JB, et al. T Cell Repertoire Dynamics during Pregnancy in Multiple Sclerosis. Cell Reports 2019;29:810-815.e814.

259. Rasmussen PV, Magyari M, Moberg JY, Bogelund M, Jensen UFA, Madsen KG. Patient awareness about family planning represents a major knowledge gap in multiple sclerosis. Mult Scler Relat Disord 2018;24:129-134.

260. Razaz N, Piehl F, Frisell T, Langer-Gould AM, McKay KA, Fink K. Disease activity in pregnancy and postpartum in women with MS who suspended rituximab and natalizumab. Neurology - Neuroimmunology Neuroinflammation 2020;7:e903.

261. Razaz N, Tremlett H, Boyce T, Guhn M, Marrie RA, Joseph KS. Incidence of Mood or Anxiety Disorders in Children of Parents with Multiple Sclerosis. Paediatric and Perinatal Epidemiology 2016;30:356-366.

262. Razaz N, Joseph KS, Boyce WT, et al. Children of chronically ill parents: Relationship between parental multiple sclerosis and childhood developmental health. Multiple Sclerosis Journal 2016;22:1452-1462.

263. Razaz N, Tremlett H, Boyce WT, Guhn M, Joseph KS, Marrie RA. Impact of parental multiple sclerosis on early childhood development: A retrospective cohort study. Multiple Sclerosis Journal 2015;21:1172-1183.

264. Razaz N, Tremlett H, Marrie RA, Joseph KS. Peripartum depression in parents with multiple sclerosis and psychiatric disorders in children. Multiple Sclerosis Journal 2016;22:1830-1840.

265. Rezaallah B, Lewis DJ, Pierce C, Zeilhofer HF, Berg BI. Social Media Surveillance of Multiple Sclerosis Medications Used During Pregnancy and Breastfeeding: Content Analysis. Journal of Medical Internet Research 2019;21:e13003-e13003.

266. Rinta S, Airas L, Elovaara I. Is the modulatory effect of pregnancy in multiple sclerosis associated with changes in blood apoptotic molecules? Acta Neurologica Scandinavica 2010;122:168-174.

267. Robertson D, Aungst A, Collier R, et al. Patient perceived changes in sexual dysfunction after initiation of natalizumab for multiple sclerosis. Multiple Sclerosis Journal Experimental, Translational and Clinical 2018;4.

268. Romero Rebecca S, Lunzmann C, Bugge J-P. Pregnancy outcomes in patients exposed to interferon beta-1b. Journal of neurology, neurosurgery, and psychiatry 2014;86:587-589.

269. Rossi C, Cicalini I, Zucchelli M, et al. Metabolomic Signature in Sera of Multiple Sclerosis Patients during Pregnancy. International Journal of Molecular Sciences 2018;19:14-14.

270. Roullet E, Verdier-Taillefer MH, Amarenco P, Gharbi G, Alperovitch A, Marteau R. Pregnancy and multiple sclerosis: a longitudinal study of 125 remittent patients. Journal of neurology, neurosurgery, and psychiatry 1993;56:1062-1065.

271. Roux T, Courtillot C, Debs R, Touraine P, Lubetzki C, Papeix C. Fecundity in women with multiple sclerosis: an observational mono-centric study. J Neurol 2015;262:957-960.

272. Runia TF, Neuteboom RF, de Groot CJM, de Rijke YB, Hintzen RQ. The influence of vitamin D on postpartum relapse and quality of life in pregnant multiple sclerosis patients. European Journal of Neurology 2015;22:479-484.

273. Runmarker B, Andersen O. Pregnancy is associated with a lower risk of onset and a better prognosis in multiple sclerosis. Brain 1995;118:253-261.

274. Sadeghi B, Motl RW, Razazian N, Khazaie H, Brand S. Aquatic exercising may improve sexual function in females with multiple sclerosis - an exploratory study. Mult Scler Relat Disord 2020;43 (no pag.

275. Sadeghpour N, Mirmosayyeb O, Bjorklund G, Shaygannejad V. Is Fertility Affected in Women of Childbearing Age with Multiple Sclerosis or Neuromyelitis Optica Spectrum Disorder? Journal of Molecular Neuroscience 2020;70:1829-1835.

276. Sadovnick AD, Eisen K, Hashimoto SA, et al. Pregnancy and multiple sclerosis. A prospective study. Arch Neurol 1994;51:1120-1124.

277. Salemi G, Callari G, Gammino M, et al. The relapse rate of multiple sclerosis changes during pregnancy: a cohort study. Acta Neurologica Scandinavica 2004;110:23-26.

278. Salhofer-Polanyi S, Wober C, Prohazka R, Dal-Bianco A, Bajer-Kornek B, Zebenholzer K. Similar impact of multiple sclerosis and migraine on sexual function in women : Is the multiple sclerosis impact scale questionnaire useful? Wiener klinische Wochenschrift 2017;129:115-120.

279. Salminen Heidi J, Leggett H, Boggild M. Glatiramer acetate exposure in pregnancy: preliminary safety and birth outcomes. J Neurol 2010;257:2020-2023.

280. Sanchez-Ramon S, Navarro A, Joaquin, et al. Pregnancy-induced expansion of regulatory T-lymphocytes may mediate protection to multiple sclerosis activity. Immunology letters 2005;96:195-201.

281. Sandberg-Wollheim M, Frank D, Goodwin TM, et al. Pregnancy outcomes during treatment with interferon beta-1a in patients with multiple sclerosis. Neurology 2005;65:802-806.

282. Sandberg-Wollheim M, Alteri E, Moraga MS, Kornmann G. Pregnancy outcomes in multiple sclerosis following subcutaneous interferon beta-1a therapy. Multiple Sclerosis Journal 2011;17:423-430.

283. Saraste M, Atula S, Hedman K, et al. Humoral response to John Cunningham virus during pregnancy in multiple sclerosis. Mult Scler Relat Disord 2018;21:11-18.

284. Saraste M, Ryynanen J, Alanen A, et al. Cerebrospinal fluid findings in multiple sclerosis patients before, during and after pregnancy [2]. Journal of Neurology, Neurosurgery and Psychiatry 2006;77:1195-1196.

285. Saraste M, Vaisanen S, Alanen A, et al. Clinical and immunologic evaluation of women with multiple sclerosis during and after pregnancy. Gender Medicine 2007;4:45-55.

286. Sargin A, Pestilci C, Ozdemir B, Tanatti O, Karaman S. Retrospective evaluation of anaesthesia methods in pregnant women with neurological and neuromuscular syndromes who underwent caesarean section. Clinical and Experimental Obstetrics and Gynecology 2017;44:700-703.

287. Schneider H, Weber CE, Hellwig K, Schroten H, Tenenbaum T. Natalizumab treatment during pregnancy - effects on the neonatal immune system. Acta Neurologica Scandinavica 2012;127:e1-4.

288. Sena A, Couderc R, Vasconcelos Joana C, Ferret-Sena V, Pedrosa R. Oral contraceptive use and clinical outcomes in patients with multiple sclerosis. J Neurol Sci 2012;317:47-51.

289. Sepulveda M, Montejo C, Llufriu S, et al. Rebound of multiple sclerosis activity after fingolimod withdrawal due to planning pregnancy: Analysis of predisposing factors. Mult Scler Relat Disord 2020;38:101483.

290. Sepulveda M, Ros C, Martinez-Lapiscina Elena H, et al. Pituitary-ovary axis and ovarian reserve in fertile women with multiple sclerosis: A pilot study. Multiple sclerosis (Houndmills, Basingstoke, England) 2016;22:564-568.

291. Shabas D, Weinreb H. Preventive healthcare in women with multiple sclerosis. Journal of Women's Health & Gender-Based Medicine 2000;9:389-395.

292. Shimizu Y, Makioka H, Harada N, Nakabayashi S, Saida T, Kira JI. Outcomes of pregnancy during interferon beta-1a therapy in Japanese patients with multiple sclerosis: Interim results of a postmarketing surveillance study. Clinical and Experimental Neuroimmunology 2015;6:402-408.

293. Sicotte NL, Liva SM, Klutch R, et al. Treatment of multiple sclerosis with the pregnancy hormone estriol. Ann Neurol 2002;52:421-428.

294. Singh V, Stingl C, Stoop Marcel P, et al. Proteomics urine analysis of pregnant women suffering from multiple sclerosis. Journal of proteome research 2015;14:2065-2073.

295. Smeltzer SC. Reproductive decision making in women with multiple sclerosis. The Journal of neuroscience nursing : journal of the American Association of Neuroscience Nurses 2002;34:145-157.

296. Smith AL, Cohen JA, Ontaneda D, Rensel M. Pregnancy and multiple sclerosis: Risk of unplanned pregnancy and drug exposure in utero. Multiple Sclerosis Journal Experimental, Translational and Clinical 2019;5.

297. Smith JB, Hellwig K, Fink K, Lyell DJ, Piehl F, Langer-Gould A. Rituximab, MS, and pregnancy. Neurology 2020;7.

298. Smith R, Studd JW. A pilot study of the effect upon multiple sclerosis of the menopause, hormone replacement therapy and the menstrual cycle. J R Soc Med 1992;85:612-613.

299. Snarski E, Snowden JA, Badoglio M, et al. Outcome of pregnancy after autologous hematopoietic stem cell transplantation (AHSCT) for autoimmune diseases (AD): A retrospective study of the EBMT autoimmune diseases working party (ADWP). Blood 2015;122.

300. Solmaz V, Ozlece HK, Him A, et al. Evaluation of the association between sexual dysfunction and demyelinating plaque location and number in female multiple sclerosis patients. Neurological Research 2018;40:683-688.

301. Spadaro M, Martire S, Marozio L, et al. Immunomodulatory Effect of Pregnancy on Leukocyte Populations in Patients With Multiple Sclerosis: A Comparison of Peripheral Blood and Decidual Placental Tissue. Frontiers in Immunology 2019;10:1935-1935.

302. Steck B, Amsler F, Grether A, et al. Mental health problems in children of somatically ill parents, e.g. multiple sclerosis. European Child and Adolescent Psychiatry 2007;16:199-207.

303. Stenager E, Stenager EN, Jensen K. Effect of pregnancy on the prognosis for multiple sclerosis. A 5-year follow up investigation. Acta Neurologica Scandinavica 1994;90:305-308.

304. Stepleman LM, Stutts L, Hudson H, Rutter MC, Williams M. Physician-patient communication about sexual functioning. Mult Scler 2016;16:S173-S173.

305. Stuifbergen A, Becker H, Rogers S, Timmerman G, Kullberg V. Promoting wellness for women with multiple sclerosis. The Journal of neuroscience nursing : journal of the American Association of Neuroscience Nurses 1999;31:73-79.

306. Tepavcevic DK, Kostic J, Basuroski ID, Stojsavljevic N, Pekmezovic T, Drulovic J. The impact of sexual dysfunction on the quality of life measured by MSQoL-54 in patients with multiple sclerosis. Mult Scler 2008;14.

307. Teter B, Kavak K, Kolb C, Zakalik K, Weinstock-Guttman B. Parity associated with long-term disease progression in women with multiple sclerosis. Neurology 2014;80.

308. Thiel S, Menck S, Elias-Hamp B, Gold R, Hellwig K. Interferon-beta exposure during first trimester in women with multiple sclerosis-a prospective cohort study from the German multiple sclerosis and pregnancy registry. Neurology 2016;86.

309. Thompson DS, Nelson LM, Burns A, Burks JS, Franklin GM. The effects of pregnancy in multiple sclerosis: A retrospective study. Neurology 1986;36:1097-.

310. Thone J, Kleiter I, Stahl A, Ellrichmann G, Gold R, Hellwig K. Relevance of endoglin, IL-1alpha, IL-1beta and anti-ovarian antibodies in females with multiple sclerosis. J Neurol Sci 2016;362:240-243.

311. Thone J, Kollar S, Nousome D, et al. Serum anti-Mullerian hormone levels in reproductive-age women with relapsing-remitting multiple sclerosis. Multiple Sclerosis Journal 2013;21:41-47.

312. Tong Y, Liu J, Yang T, et al. Influences of pregnancy on neuromyelitis optica spectrum disorders and multiple sclerosis. Mult Scler Relat Disord 2018;25:61-65.

313. Trenova Anastasiya G, Slavov Georgy S, Manova Maria G, Kostadinova Ivanka I, Vasileva Tonka V. Female sex hormones and cytokine secretion in women with multiple sclerosis. Neurological Research 2013;35:95-99.

314. Triantafyllou N, Thoda P, Armeni E, et al. Association of sex hormones and glucose metabolism with the severity of multiple sclerosis. The International journal of neuroscience 2016;126:797-804.

315. Triplett JD, Vijayan S, Rajanayagam S, Tuch P, Kermode AG. Pregnancy outcomes amongst multiple sclerosis females with third trimester natalizumab use. Mult Scler Relat Disord 2020;40 (no pag.

316. Tudor KI, Eames S, Haslam C, Chataway J, Liechti MD, Panicker JN. Identifying barriers to help-seeking for sexual dysfunction in multiple sclerosis. J Neurol 2018;265:2789-2802.

317. Tuohy O, Costelloe L, Hill-Cawthorne G, et al. Alemtuzumab treatment of multiple sclerosis: Long-term safety and efficacy. Journal of Neurology, Neurosurgery and Psychiatry 2015;86:208-215.

318. Turk B, Koseoglu T, Boluk C, Bilgic A, Tasdemir M. Effects of multiple sclerosis and medications on menopausal age. Journal of International Medical Research 2018;46:1249-1253.

319. Twork S, Wirtz M, Schipper S, Klewer J, Bergmann A, Kugler J. Chronical illness and maternity: Life conditions, quality of life and coping in women with multiple sclerosis. Qual Life Res 2007;16:1587-1594.

320. Tzortzis V, Skriapas K, Hadjigeorgiou G, et al. Sexual dysfunction in newly diagnosed multiple sclerosis women. Multiple sclerosis (Houndmills, Basingstoke, England) 2008;14:561-563.

321. Valiani M, Ashtari F, Mokhtari F. Reciprocal effects of multiple sclerosis, childbirth, and postpartum. Iranian Journal of Neonatology 2018;9:76-82.

322. Valleroy ML, Kraft GH. Sexual dysfunction in multiple sclerosis. Arch Phys Med Rehabil 1984;65:125-128.

323. van der Kop Mia L, Pearce Mark S, Dahlgren L, et al. Neonatal and delivery outcomes in women with multiple sclerosis. Ann Neurol 2011;70:41-50.

324. Vanya M, Nyari T, Bencsik K, Bartfai G. Pregnancy and perinatal outcomes among women with multiple sclerosis: a retrospective case-controlled study in South Hungary. J Matern Fetal Neona 2014;27:577-581.

325. Verdier-Taillefer MH, Alperovitch A. The relationship of patients' sex to reproduction in multiple sclerosis. Journal of Epidemiology and Community Health 1990;44:77-77.

326. Verdru P, Theys P, D'Hooghe MB, Carton H. Pregnancy and multiple sclerosis: the influence on long term disability. Clinical Neurology and Neurosurgery 1994;96:38-41.

327. Villaverde-Gonzalez R, Candeliere-Merlicco A, Alonso-Frias MA, et al. Discontinuation of disease-modifying treatments in multiple sclerosis to plan a pregnancy: A retrospective registry study. Mult Scler Relat Disord 2020;46 (no pag.

328. Voskuhl RR, Wang H, Wu TCJ, et al. Estriol combined with glatiramer acetate for women with relapsing-remitting multiple sclerosis: A randomised, placebo-controlled, phase 2 trial. Lancet Neurol 2016;15:35-46.

329. Vukusic S, Coyle PK, Jurgensen S, et al. Pregnancy outcomes in patients with multiple sclerosis treated with teriflunomide: Clinical study data and 5 years of post-marketing experience. Multiple Sclerosis Journal 2020;26:829-836.

330. Vukusic S, Hutchinson M, Hours M, et al. Pregnancy and multiple sclerosis (the PRIMS study): Clinical predictors of post-partum relapse. Brain 2004;127:1353-1360.

331. Vukusic S, Durand-Dubief F, Benoit A, Marignier R, Frangoulis B, Confavreux C. Natalizumab for the prevention of post-partum relapses in women with multiple sclerosis. Multiple Sclerosis Journal 2015;21:953-955.

332. Webb EJD, Meads D, Eskyte I, et al. The Impact of Reproductive Issues on Preferences of Women with Relapsing Multiple Sclerosis for Disease-Modifying Treatments. Patient 2020;13:583-597.

333. Weber-Schoendorfer C, Schaefer C. Multiple sclerosis, immunomodulators, and pregnancy outcome: a prospective observational study. Multiple Sclerosis Journal 2009;15:1037-1042.

334. Wei T, Lightman SL. The neuroendocrine axis in patients with multiple sclerosis. Brain 1997;120:1067-1076.

335. Weinshenker BG, Hader W, Carriere W, Baskerville J, Ebers GC. The influence of pregnancy on disability from multiple sclerosis: a population-based study in Middlesex County, Ontario. Neurology 2006;39:1438-1440.

336. Winder K, Linker RA, Seifert F, et al. Neuroanatomic Correlates of Female Sexual Dysfunction in Multiple Sclerosis. Ann Neurol 2016;80:490-498.

337. Wingerchuk DM, Rodriguez M. Premenstrual multiple sclerosis pseudoexacerbations. Role of body temperature and prevention with aspirin. Arch Neurol 2006;63:1005-1008.

338. Winkelmann A, Rommer PS, Hecker M, Zettl UK. Intravenous immunoglobulin treatment in multiple sclerosis: A prospective, rater-blinded analysis of relapse rates during pregnancy and the postnatal period. CNS Neuroscience & Therapeutics 2018;25:78-85.

339. Worthington J, Jones R, Crawford M, Forti A. Pregnancy and multiple sclerosis--a 3-year prospective study. J Neurol 1994;241:228-233.

340. Wu Q, Chen B, Liu N, et al. Insights into Initial Demyelinating Episodes of Central Nervous System during Puerperium. Chinese medical journal 2017;130:1791-1795.

341. Yalcin SE, Yalcin Y, Yavuz A, Akkurt MO, Sezik M. Maternal and perinatal outcomes in pregnancies with multiple sclerosis: A case-control study. Journal of Perinatal Medicine 2017;45:455-460.

342. Young CA, Tennant A, Mills RJ, Rog DJ, Ford HL, Orchard K. Sexual functioning in multiple sclerosis: Relationships with depression, fatigue and physical function. Multiple Sclerosis Journal 2016;23:1268-1275.

343. Zafarmand S, Javanmardi H, Ameri M, et al. Evaluation of the neurological complaints during pregnancy and postpartum. Galen Medical Journal 2019;8 (no pagi.

344. Zaherian N, Mousavi P, Majdinasab N, Haghighizadeh MH, Afshari P. The effect of pelvic floor muscle exercises on sexual function in women with multiple sclerosis: A pre-/post-intervention clinical trial. Family Medicine and Primary Care Review 2020;22:179-182.

345. Zakrzewska-Pniewska B, Golebiowski M, Zajda M, Szeszkowski W, Podlecka-Pietowska A, Nojszewska M. Sex hormone patterns in women with multiple sclerosis as related to disease activity--a pilot study. Neurologia i neurochirurgia polska 2011;45:536-542.

346. Zanghi A, D'Amico E, Callari G, et al. Pregnancy and the Postpartum Period in Women With Relapsing-Remitting Multiple Sclerosis Treated With Old and New Disease-Modifying Treatments: A Real-World Multicenter Experience. Frontiers in Neurology 2020;11 (no pag.

347. Zavoreo I, Grzincic T, Preksavec M, Madzar T, Kes VB. Sexual dysfunction and incidence of depression in multiple sclerosis patients. Acta Clinica Croatica 2016;55:402-406.

348. Zengin K, Boz C, Terzi M, et al. Methylprednisolone Concentrations in Breast Milk and Serum of Patients with Multiple Sclerosis Treated with IV Pulse Methylprednisolone. Clinical Neurology and Neurosurgery 2020;197 (no pa.

349. Zivadinov R, Zorzon M, Bosco A. Sexual dysfunction in multiple sclerosis: II. Correlation analysis. Mult Scler 1999;5.

350. Zivadinov R, Zorzon M, Locatelli L. Sexual dysfunction in multiple sclerosis: a MRI, neurophysiological and urodynamic study. J Neurol Sci 2003;210.

351. Zorgdrager A, De Keyser J. Menstrually related worsening of symptoms in multiple sclerosis. J Neurol Sci 1997;149:95-97.

352. Zorgdrager A, De Keyser J. The premenstrual period and exacerbations in multiple sclerosis. European neurology 2002;48:204-206.

353. Zorzon M, Zivadinov R, Bosco A. Sexual dysfunction in multiple sclerosis: a case-control study. I. Frequency and comparison of groups. Mult Scler 1999;5.

354. Zuluaga MI, Otero-Romero S, Rovira A, et al. Menarche, pregnancies, and breastfeeding do not modify long-term prognosis in multiple sclerosis. Neurology 2019;92:e1507-e1516.
